# Specificity, length and luck drive gene rankings in association studies

**DOI:** 10.1038/s41586-025-09703-7

**Published:** 2025-11-05

**Authors:** Jeffrey P. Spence, Hakhamanesh Mostafavi, Mineto Ota, Nikhil Milind, Tamara Gjorgjieva, Courtney J. Smith, Yuval B. Simons, Guy Sella, Jonathan K. Pritchard

**Affiliations:** 1 Department of Genetics, Stanford University, Stanford, CA USA; 2 Institute for Human Genetics, University of California, San Francisco, San Francisco, CA USA; 3 Department of Epidemiology & Biostatistics, University of California, San Francisco, San Francisco, CA USA; 4 Center for Human Genetics and Genomics, New York University School of Medicine, New York, NY USA; 5 Department of Population Health, New York University School of Medicine, New York, NY USA; 6 Department of Allergy and Rheumatology, Graduate School of Medicine, The University of Tokyo, Tokyo, Japan; 7 Section of Genetic Medicine, University of Chicago, Chicago, IL USA; 8 Department of Human Genetics, University of Chicago, Chicago, IL USA; 9 Department of Biological Sciences, Columbia University, New York, NY USA; 10 Program for Mathematical Genomics, Columbia University, New York, NY USA; 11 Department of Biology, Stanford University, Stanford, CA USA

**Keywords:** Genome-wide association studies, Evolutionary biology, Genetics research, Population genetics, Statistical methods

## Abstract

Standard genome-wide association studies (GWAS) and rare variant burden tests are essential tools for identifying trait-relevant genes^1^. Although these methods are conceptually similar, by analysing association studies of 209 quantitative traits in the UK Biobank^2–4^, we show that they systematically prioritize different genes. This raises the question of how genes should ideally be prioritized. We propose two prioritization criteria: (1) trait importance — how much a gene quantitatively affects a trait; and (2) trait specificity — the importance of a gene for the trait under study relative to its importance across all traits. We find that GWAS prioritize genes near trait-specific variants, whereas burden tests prioritize trait-specific genes. Because non-coding variants can be context specific, GWAS can prioritize highly pleiotropic genes, whereas burden tests generally cannot. Both study designs are also affected by distinct trait-irrelevant factors, complicating their interpretation. Our results illustrate that burden tests and GWAS reveal different aspects of trait biology and suggest ways to improve their interpretation and usage.

## Abstract (web summary)

Genetic association tests prioritize candidate genes based on different criteria.

## Main

A central goal of human genetics is to identify which genes affect traits and disease risk and to what extent. This is essential for addressing fundamental questions such as what biological processes underlie trait variation, which genes and pathways are most critical for understanding those processes, and which genes could serve as potential therapeutic targets.

Although many techniques exist to study gene function in model systems or in vitro (for example, see refs. ^5–7^), the study of organism-level traits in humans largely relies on naturally occurring genetic variation, primarily through GWAS^1^.

GWAS have been deeply informative about the genetic basis of complex traits, from uncovering actionable drug targets^8^ to identifying trait-relevant cell types and programs^9–12^. However, it remains unclear how best to extract biological insight from GWAS. First, GWAS do not directly pinpoint relevant genes, as most associated variants are non-coding^13^. Moreover, a surprisingly large fraction of the genome contributes to the heritability of many traits^14–16^, and trait-associated variants often cannot be mapped to genes with clear phenotypic relevance.

Recently, large whole-exome and whole-genome sequencing datasets have enabled the direct study of genes through rare protein-coding variants, which are excluded or underpowered in GWAS^3^. To boost statistical power, these variants are analysed using burden tests^4,17^. Burden tests aggregate variants — typically loss-of-function (LoF) variants — within a gene to create a ‘burden genotype’, which is then tested gene by gene for association with phenotypes. This is similar to common-variant GWAS but focused on rare variants combined at the gene level.

Despite this conceptual similarity, recent work has found anecdotally that for many traits, LoF burden tests and GWAS discover distinct genes, although with some overlap^18,19^. In a systematic analysis, Weiner et al. found that for many traits, burden heritability is explained by fewer genes than are required to explain single-nucleotide polymorphism (SNP) heritability, and burden tests tend to prioritize genes that are seemingly more closely related to trait biology^20^.

To better understand these differences, we analysed the results of GWAS and LoF burden tests for 209 quantitative traits in the UK Biobank^2–4^. We showed that burden tests and GWAS prioritize different genes, and these differences persist even when conservatively addressing power differences and issues linking variants to genes.

The discrepancy between GWAS and LoF burden tests raises thorny questions, such as what criteria each method uses to prioritize genes, and how these criteria relate to the underlying biology; which method is more relevant for understanding trait biology; and which method is better suited for downstream applications, such as drug target discovery.

We analysed association study results and used population genetics models to address these questions. Our results show that burden tests tend to prioritize trait-specific genes — those primarily affecting the studied trait with little effect on other traits — whereas GWAS also capture more pleiotropic genes often missed by burden tests. In addition, we highlight the effect of trait-irrelevant factors on discovery, particularly gene length and random genetic drift. Ultimately, GWAS and LoF burden tests reveal distinct but complementary aspects of trait biology, with important implications for interpreting and using association studies.

## Burden test and GWAS gene ranks differ

**Fig. 1:**
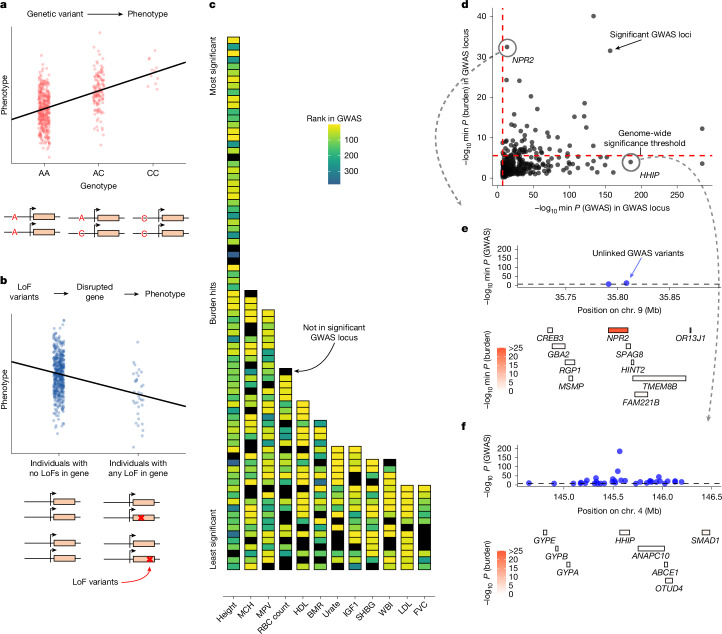
GWAS and LoF burden tests prioritize different loci. **a**,**b**, Schematics of GWAS (**a**) and LoF burden tests (**b**). **c**, Each cell is a genome-wide significant gene according to LoF burden tests, ordered by significance (most significant (top) to least significant (bottom)). Genes are coloured by the rank of the locus that contains the gene according to GWAS or are coloured black if they are not contained in any genome-wide significant locus. Across 151 traits with at least one burden hit and one GWAS hit, 74.6% (1,382 out of 1,852) of genome-wide significant burden test hits fall within a GWAS locus. Trait abbreviations are defined in Supplementary Table 1. **d**, Minimum LoF burden test *P* values for any gene overlapping a genome-wide significant GWAS locus plotted against the minimum GWAS *P* value within that locus (*n* = 382 loci). **e**, The genomic region surrounding *NPR2*. GWAS *P* values of approximately independent genome-wide significant GWAS hits (top), and the location of genes coloured by LoF burden test *P* values (bottom) are shown. **f**, Similar to panel **e** but for the genomic region surrounding *HHIP*.

GWAS and LoF burden tests are conceptually similar (Fig. 1a,b), but previous studies have highlighted key differences in their findings^20^. To more thoroughly quantify how concordantly these methods prioritize genes and genomic loci using *P* values, we systematically compared GWAS and LoF burden test results for 209 quantitative traits from the UK Biobank (Methods)^4^.

In principle, technical artefacts could drive discordance between GWAS and LoF burden test results. The causal genes driving GWAS hits are usually unknown, and errors in linking hits to genes could reduce the overlap between genes prioritized by the two study designs.

To minimize these technical effects, we maximized concordance whenever possible. We conservatively defined GWAS loci by taking a 1-Mb window around each genome-wide significant GWAS hit and merging overlapping windows. We ranked these loci by the minimum GWAS *P* value within each locus and, for each genome-wide significant gene in the burden test, compared its rank based on burden *P* value to the rank of the GWAS locus (if any) that contained it.

Consistent with previous reports^19,21^, we found that the majority of burden hits fall within a GWAS locus (Fig. 1c). Yet, the two association study designs rank genes very differently. Some of the top burden hits fall outside the defined GWAS loci or are within GWAS loci that are ranked lower than hundreds of other loci (Fig. 1c). We quantified this difference in ranking by calculating how many burden hits fall within ‘top’ GWAS loci (Methods), finding that only 26% (480 out of 1,852) of genes with burden support fall in the top GWAS loci (Supplementary Fig. 1).

To gain intuition about the source of these discordant rankings, we considered height as an example trait, for which there are 382 genome-wide significant GWAS loci (Fig. 1d). The rankings from the two study designs are somewhat concordant (Spearman’s $\rho =0.46$), suggesting that they are not uncovering totally disparate axes of biology. Yet, there is little overlap in the top hits, with many significant GWAS loci not containing a single significant burden gene. This pattern is not unique to height (Supplementary Figs. 2 and 3), and these results are robust to the details of the analysis choices (Supplementary Appendix A and Supplementary Figs. 4–31).

We can illustrate these differences with two examples of discordantly ranked loci. Figure 1e shows the *NPR2* locus. *NPR2* is the second most significant gene in the LoF burden tests, but it is contained in the 243rd most significant GWAS locus. It is unsurprising that this locus is significant in both association tests: mutations in *NPR2* have been linked to short stature in humans and mice^22–27^. Yet, hundreds of loci are more strongly prioritized by GWAS, including the *HHIP* locus (Fig. 1f). The *HHIP* locus is the third most significant locus and has numerous uncorrelated GWAS hits (*r*^2^ < 0.1) with *P* values as small as 10^−185^. *HHIP* is a biologically sensible hit for height^28^ as HHIP has been implicated in osteogenesis^29^ and interacts with three different Hedgehog proteins^30,31^, which are involved in body patterning and limb formation^32^. Nonetheless, there is essentially no burden signal for *HHIP* or any of the other genes in the locus. These differences motivated us to explore why GWAS and LoF burden tests might rank loci so differently.

## How should genes be prioritized?

Given the extensive differences in how GWAS and LoF burden tests rank genes, we are faced with an underexplored question: if we could precisely measure any quantity of interest for each gene, what properties would make us want to rank one gene higher than another for a given trait. That is, how should genes ideally be prioritized?

**Fig. 2:**
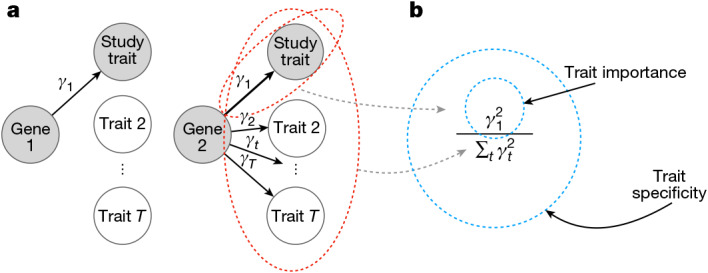
How should genes be prioritized? **a**, A cartoon of two genes that affect a trait under study. The widths of the arrows represent the relative effect sizes. Gene 1 is more trait specific, but gene 2 is more trait important. **b**, Formal definitions of trait importance and trait specificity for genes in the context of LoF burden tests. The effect of an LoF in the gene on trait $t$ is ${\gamma }_{t}$, with trait 1 being the study trait. We have defined trait importance as ${\gamma }_{1}^{2}$ and trait specificity as ${\gamma }_{1}^{2}\,/\,{\sum }_{t}{\gamma }_{t}^{2}$.

We propose two distinct ranking criteria: trait importance and trait specificity. Imagine a gene that is only expressed in developing bones and whose disruption results in shorter stature but has minimal effects on other traits (Fig. 2a). In some sense, this is a quintessential ‘height gene’, and we might want this gene to be highly ranked in association studies. Conversely, consider a broadly expressed transcription factor whose disruption results in an even greater reduction of height, but also disrupts the normal functioning of numerous organ systems. This is less obviously a height gene, but it has a larger effect on height than the first gene. We have defined trait specificity and trait importance such that the first gene has higher trait specificity, but the second gene has higher trait importance.

Formally, we have defined the trait importance of a variant as its squared effect on the trait of interest, considering high-impact variants important regardless of their direction of effect. We have defined the trait importance of a gene as the trait importance of LoF variants in that gene. Throughout, we used ${\alpha }_{t}$ to refer to the effect size of a variant on trait $t$, and ${\gamma }_{t}$ to refer to the LoF burden effect size of a gene; that is, trait importance for trait 1 is denoted as ${\alpha }_{1}^{2}$ and ${\gamma }_{1}^{2}$ for variants and genes, respectively. Throughout, we took the trait under study to be trait 1.

We have defined trait specificity as the importance for the trait of interest relative to the importance across all fitness-relevant traits measured in appropriate units (Fig. 2b). We have denoted trait specificity by ${{\rm{\Psi }}}_{V}:= {\alpha }_{1}^{2}\,/\,{\sum }_{t}{\alpha }_{t}^{2}$ for variants and ${{\rm{\Psi }}}_{G}:= {\gamma }_{1}^{2}\,/\,{\sum }_{t}{\gamma }_{t}^{2}$ for genes. See Supplementary Appendix B for more details. Ideally, association studies would prioritize genes based on trait importance, trait specificity or some combination thereof.

## Burden tests favour trait-specific genes

**Fig. 3:**
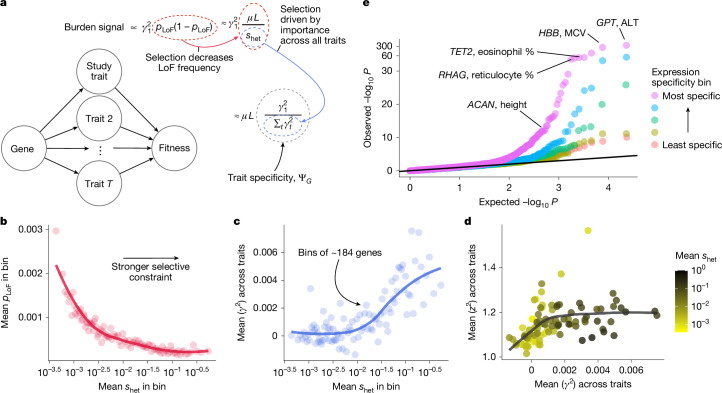
Burden tests prioritize trait-specific genes, not large-effect genes. **a**, Burden tests prioritize genes by trait specificity. $\mu$ is the per-site mutation rate, $L$ is the number of potential LoF positions, and ${s}_{\mathrm{het}}$ is the strength of selection against heterozygous LoF carriers. **b**, Genes were binned by estimated *s*_het_^ (ref. 35^) with approximately 184 genes per bin. Aggregate LoF frequencies were averaged across genes within each bin. The trend line was fit using LOESS. Spearman’s $\rho$ between posterior mean ${s}_{\mathrm{het}}$ and *p*_LOF_ = −0.547; *P* < 10^−15^; *n* = 18,154 genes. **c**, Similar to panel **b** but averaging over an unbiased estimate of the mean of ${\gamma }_{t}^{2}$ across traits. Pearson’s $r$ between posterior mean ${s}_{\mathrm{het}}$ and unbiased estimate of ${\sum }_{t}{\gamma }_{t}^{2}=0.078$; *P* < 10^−15^; *n* = 18,154 genes. **d**, Genes were binned as in panel **b**, and the mean of squared *z*-scores, ${z}^{2}$, across traits was plotted against the average of an unbiased estimate of the mean of ${\gamma }_{t}^{2}$ across traits. Points are coloured by the mean ${s}_{\mathrm{het}}$ within the bin and the trend line was fit using LOESS. Pearson’s $r$ between mean importance and mean ${z}^{2}$ across the 25 highest ${s}_{\mathrm{het}}$ bins = 0.188, *P* = 0.368. Note that this correlation is probably overestimated as the noise in estimated importance and ${z}^{2}$ is correlated. **e**, Quantile–quantile plot of LoF burden test *P* values across nine trait–tissue pairs. Genes were stratified for each trait–tissue pair based on the specificity of their expression to the trait-relevant tissue. The *y* axis has been non-linearly transformed. One-sided Wilcoxon test *P* < 10^−5^ for all comparisons of the distribution of *P* values in the most expression-specific bin to each other bin. The five bins contained *n* = 11,596, *n* = 11,441, *n* = 11,185, *n* = 11,477 and *n* = 11,470 *P* values from the least-specific to most-specific bins, respectively.

To determine how LoF burden tests prioritize genes, we analysed population genetics models of complex traits developed by Simons et al.^33^ (Supplementary Appendix B). Our analysis revealed that LoF burden tests prioritize genes in part by their trait specificity, and not by importance (Fig. 3a). We briefly outline the argument here.

In burden tests, the strength of association, ${z}^{2}$, for a gene depends on both its trait importance, ${\gamma }_{1}^{2}$, and the aggregate frequency of LoFs, ${p}_{\mathrm{LoF}}$, with the expected strength of association being proportional to ${\gamma }_{1}^{2}{p}_{\mathrm{LoF}}(1-{p}_{\mathrm{LoF}})$.

Natural selection acts to keep LoFs rare: for sufficiently strong selection, ${p}_{\mathrm{LoF}}(1-{p}_{\mathrm{LoF}})$ is proportional to $\mu L/{s}_{\mathrm{het}}$, where $\mu$ is the per-base mutation rate, $L$ is the number of sites where an LoF could occur, and ${s}_{\mathrm{het}}$ is the strength of selection in heterozygotes^34^. As expected, there is a strong negative relationship between estimates of *s*_het_^35^ and the average of ${p}_{\mathrm{LoF}}$ across genes within ${s}_{\mathrm{het}}$ bins (Fig. 3b and Supplementary Fig. 32).

Furthermore, many complex traits are thought to be under stabilizing selection^36–39^. Crucially, this predicts a connection between ${s}_{\mathrm{het}}$ and total trait effects. Specifically, ${s}_{\mathrm{het}}\approx {\sum }_{t}{\gamma }_{t}^{2}$ where ${\sum }_{t}{\gamma }_{t}^{2}$ is the sum of trait importances across all fitness-relevant traits measured in appropriate units (Supplementary Appendix B). To test this, we computed unbiased estimates of trait importance from LoF burden test results for 27 genetically uncorrelated traits (Methods). The average trait importance across these traits shows a strongly significant positive relationship with ${s}_{\mathrm{het}}$ as predicted by our model (Fig. 3c).

Combining these results, the strength of association in LoF burden tests is proportional to ${\gamma }_{1}^{2}\,/\,{\sum }_{t}{\gamma }_{t}^{2}$ (Fig. 3a), exactly our definition of ${{\rm{\Psi }}}_{G}$, the trait specificity of the gene.

A key implication is that LoF burden tests do not prioritize genes based on trait importance. The most trait-important genes will often be the most constrained and have the smallest frequencies and hence largest standard errors, an effect previously referred to as flattening^33,40^. Indeed, Fig. 3d shows that, for genes with sufficiently large effects, the strength of association (${z}^{2}:= {(\hat{{\gamma }_{1}}/{\rm{SE}}(\hat{{\gamma }_{1}}))}^{2}$) is completely decoupled from trait importance in the UK Biobank LoF burden tests.

Instead, our theory predicts that LoF burden tests prioritize genes by their trait specificity, ${{\rm{\Psi }}}_{G}$. To confirm this prediction, we would ideally compare strength of association to an independent measure of ${{\rm{\Psi }}}_{G}$. It is difficult to directly estimate ${{\rm{\Psi }}}_{G}$ independently of our theory as ${{\rm{\Psi }}}_{G}$ depends on the unknown true trait importances. Instead, we used how specifically expressed a gene is as an imperfect proxy.

We focused on nine traits that we could confidently assign to a single causal tissue or cell type, and binned genes based on their expression in the causal tissue relative to their average expression across considered tissues (Methods).

Using results from the LoF burden tests for these nine trait–tissue pairs, we constructed a quantile–quantile plot (Fig. 3e). Consistent with our prediction, we observed significantly stronger signals in the most specific expression bins. We also observed that many of the top hits are plausibly trait specific. Our theory predicts that this relationship between specificity and power should hold regardless of ${s}_{\mathrm{het}}$, which we confirmed empirically (Supplementary Fig. 34).

We found qualitatively similar results when using burden tests that include both LoFs and probably deleterious missense variants (Supplementary Figs. 33 and 35), although the relationship between ${s}_{\mathrm{het}}$ and ${\gamma }^{2}$ is less pronounced, presumably due to a known artefact from using variants with different effect sizes^20^. We also obtained similar results using a regression model to predict LoF burden ${z}^{2}$ from expression specificity while controlling for effect sizes (Supplementary Fig. 36).

## GWAS prioritize trait-specific variants

We next turned to GWAS. In contrast to LoF burden tests, GWAS are performed at the variant level, and so we considered what drives the rankings of variants. Following the same argument as above reveals that the expected strength of association is proportional to ${\alpha }_{1}^{2}\,/\,{\sum }_{t}{\alpha }_{t}^{2}$, the trait importance of the variant for the trait under study relative to the total trait importance of the variant across all fitness-relevant traits. This is exactly ${{\rm{\Psi }}}_{V}$, the trait specificity of the variant.

**Fig. 4:**
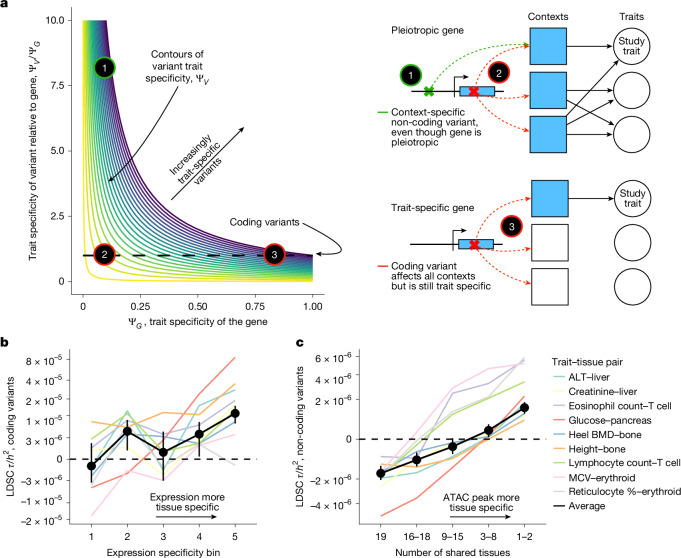
GWAS prioritize trait-specific variants. **a**, Schematic of what determines trait specificity for variants, ${{\rm{\Psi }}}_{V}$. ${{\rm{\Psi }}}_{V}$ is determined by two components: the trait specificity of the gene that the variant acts through, and the trait specificity of the variant relative to that gene. Three representative types of variants are highlighted with gene models. The green variant is non-coding, whereas the red variants are coding. Shaded contexts represent cellular contexts or cell types in which the gene affects traits. **b**, Heritability enrichment for coding variants as measured by S-LDSC $\tau$ as a function of expression specificity for nine trait–tissue pairs. The inverse variance-weighted average of the results for the individual traits is in black. Black dashed line represents no effect on heritability. The *y* axis has been non-linearly transformed. One-sided *Z*-test *P* < 0.009 for all comparisons between $\tau$ in the most specifically expressed bin and each other bin. **c**, Heritability enrichment for non-coding variants in ATAC peaks as measured by S-LDSC $\tau$ as a function of ATAC peak tissue specificity for nine trait–tissue pairs. The inverse variance-weighted average of the results for the individual traits is in black. Black dashed line represents no effect on heritability. The *y* axis has been non-linearly transformed. One-sided *Z*-test *P* < 0.004 for all comparisons between $\tau$ in the most specifically expressed bin and each other bin.

The fact that GWAS prioritizes trait-specific variants rather than genes has profound implications for understanding the differences between GWAS and LoF burden tests. In particular, variants can be trait specific in two ways (Fig. 4a): they can either affect a trait-specific gene (variant 3 in Fig. 4a) or have context-specific effects on a pleiotropic gene (variant 1 in Fig. 4a). For example, context-specific variants might regulate expression only in trait-relevant cell types or developmental time points, resulting in trait-specific effects even when acting on pleiotropic genes. In Supplementary Appendix C, we developed a model formalizing the relationship between ${{\rm{\Psi }}}_{V}$, ${{\rm{\Psi }}}_{G}$ and context-specific expression.

To test our predictions, we considered these two ways that variants can be trait specific and used stratified linkage disequilibrium (LD) score regression (S-LDSC)^9,41^ to quantify how heritability changes along these axes. The average heritability contributed by a set of variants is a proxy for how highly those variants would be prioritized by GWAS on average. We quantified effects on heritability by $\tau$ as reported by S-LDSC normalized by heritability, which can be interpreted as how much a given annotation increases the per-variant proportion of heritability explained conditioned on all other annotations (Methods).

First, we looked into whether the trait specificity of the gene on which a variant acts affects GWAS prioritization for variants with a given context specificity (moving along the horizontal axis of Fig. 4a). To this end, we restricted our analyses to coding variants and used our measure of expression specificity as a proxy for ${{\rm{\Psi }}}_{G}$. Overall, variants acting on specifically expressed genes are prioritized higher by GWAS (Fig. 4b and Supplementary Figs. 37 and 38).

We next examined the effect of context specificity (moving along the vertical axis of Fig. 4a). We used non-coding variants, and used the tissue specificity of assay for transposase-accessible chromatin using sequencing (ATAC-seq) peaks as an imperfect proxy for trait specificity. We estimated the effect of ATAC specificity on heritability using S-LDSC while controlling for the strength of the ATAC peaks (Methods).

Across all nine traits, we saw a significant trend of increasing contribution to heritability in more tissue-specific ATAC peaks (Fig. 4c and Supplementary Figs. 39 and 40), even when conditioning on ${s}_{\mathrm{het}}$ (Supplementary Figs. 41 and 42; total contributions to heritability instead of enrichment in Supplementary Fig. 43).

Overall, our results show that LoF burden tests and GWAS both prioritize trait specificity, but prioritize different loci because LoF burden tests rank genes, whereas GWAS rank variants. Variants can be specific either by acting through trait-specific genes or by being context specific, and both of these axes contribute to GWAS prioritization. The difference between LoF burden tests and GWAS is not driven by differences in the frequencies of the variants they consider, but rather by GWAS including non-coding variants, which can be context specific, and LoF burden tests including only coding variants. This explains why LoF burden tests and GWAS prioritize different loci even when restricting to lower-frequency variants in GWAS (Supplementary Figs. 26–28).

## LoF burden tests prioritize long genes

Our modelling also revealed factors beyond trait specificity that affect how GWAS and LoF burden tests prioritize genes. These factors have nothing to do with any aspect of trait biology: LoF burden tests prioritize genes in part by the length of their coding sequence, and GWAS prioritize variants in part due to randomness in their frequencies caused by genetic drift.

LoF burden tests aggregate all LoF variants within a gene (Fig. 1b). As we derived above, this results in an expected strength of association that increases with $\mu L$, the average mutation rate multiplied by the number of potential LoF positions within a gene. Intuitively, if a gene has more potential LoFs, then the proportion of individuals that are LoF carriers will be larger, resulting in greater power, all else being equal.

**Extended Data Fig. 1:**
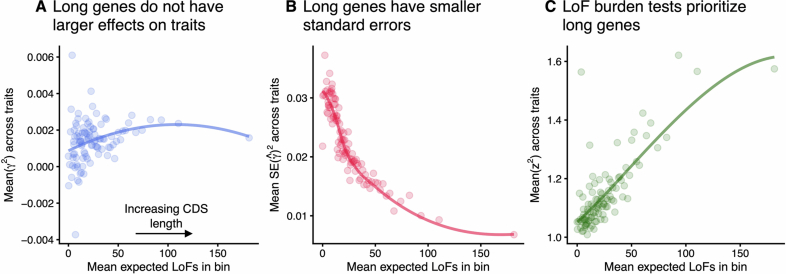
Coding sequence length drives prioritization in LoF burden tests. **A**) Average of an unbiased estimate of the squared trait importance, $${\gamma }^{2}$$, across 27 genetically uncorrelated traits, averaged within bins of approximately 184 genes binned by expected number of unique LoFs (Methods). The trend line was fit using LOESS. Pearson’s $$r$$ between expected number of unique LoFs and an unbiased estimate of mean squared effect size across traits $$=\,0.017$$, p-value $$=\,0.023$$, $$N=\mathrm{18,344}$$ genes. This analysis was repeated for **B**) the average of the squared LoF burden test standard errors within each bin (Spearman’s $$\rho $$ between expected number of unique LoFs and mean standard error across traits $$=-\,0.255$$, p-value $$ < {10}^{-15}$$, $$N=\mathrm{18,344}$$ genes), and **C**) the average LoF burden test $${z}^{2}$$ across traits within each bin (Pearson’s $$r$$ between expected number of unique LoFs and mean $${z}^{2}$$ across traits $$=\,0.112$$, p-value $$ < {10}^{-16}$$, $$N=\mathrm{18,344}$$ genes).

We confirmed these predictions using the UK Biobank LoF burden tests. In particular, longer genes generally do not have substantially larger effect sizes (Extended Data Fig. 1a), but do have considerably smaller standard errors (Extended Data Fig. 1b), resulting in a significant effect of gene length on burden signal, ${z}^{2}$ (Extended Data Fig. 1c). This also causes longer genes to be hits for more traits, causing them to appear more pleiotropic, despite not having larger effects across more traits. See Supplementary Appendix D for details.

## Random genetic drift affects GWAS

**Extended Data Fig. 2:**
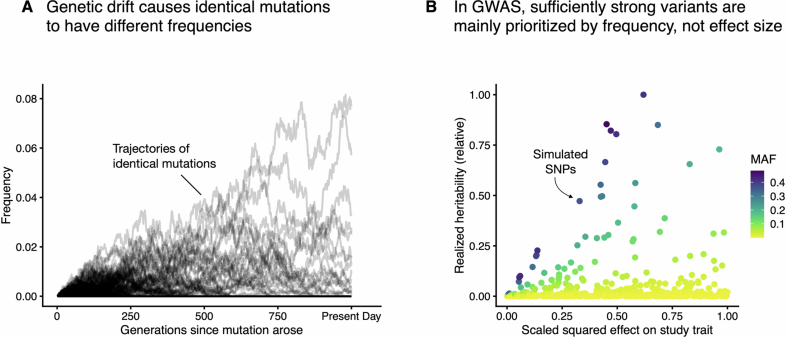
GWAS variant rankings are driven largely by genetic drift. **A**) $\mathrm{10,000}$ frequency trajectories of identical mutations simulated under the Discrete-Time Wright-Fisher model. Trajectories were simulated assuming no recurrent mutation, an ${s}_{\mathrm{het}}$ of ${10}^{-3}$, no fitness consequences in homozygotes, and a population size of ${N}_{e}=\mathrm{10,000}$. All mutations were assumed to arise $\mathrm{1,000}$ generations before present. **B**) Simulations of realized heritability for individual variants with varying trait importances, scaled by the maximum simulated realized heritability. Spearman’s $\rho =0.052$, p-value $=\,0.348$ for $N=330$ variants with a scaled squared effect >0.25.

We showed above that the expected strength of association in GWAS is proportional to the trait specificity of a variant, ${{\rm{\Psi }}}_{V}$. This is true on average, but there is considerable variation around this expectation. In well-powered GWAS, variants are ranked by $2{\alpha }_{1}^{2}p(1-p)$, where $p$ is the variant allele frequency (Supplementary Appendix B). We refer to $2{\alpha }_{1}^{2}p(1-p)$ as the realized heritability of a variant. Under our modelling assumptions, the expected value of $p(1-p)$ is proportional to $1/\,{\sum }_{t}{\alpha }_{t}^{2}$ (Supplementary Appendix G), resulting in trait-specific variants being ranked more highly on average. Yet, random genetic drift causes variant frequencies to be spread widely around their expected values (Extended Data Fig. 2a).

In LoF burden tests, this effect is largely ameliorated by the aggregation of variants, which averages out the stochasticity in the frequencies of individual LoFs (Supplementary Appendix B). However, GWAS consider variants one at a time, causing this stochasticity to have a large role in gene prioritization.

Indeed, in simulated GWAS, the ranking of variants in terms of realized heritability is largely random with respect to trait importance for sufficiently trait-important variants (Extended Data Fig. 2b), driven by differences in minor allele frequency (MAF) due to genetic drift.

**Extended Data Fig. 3:**
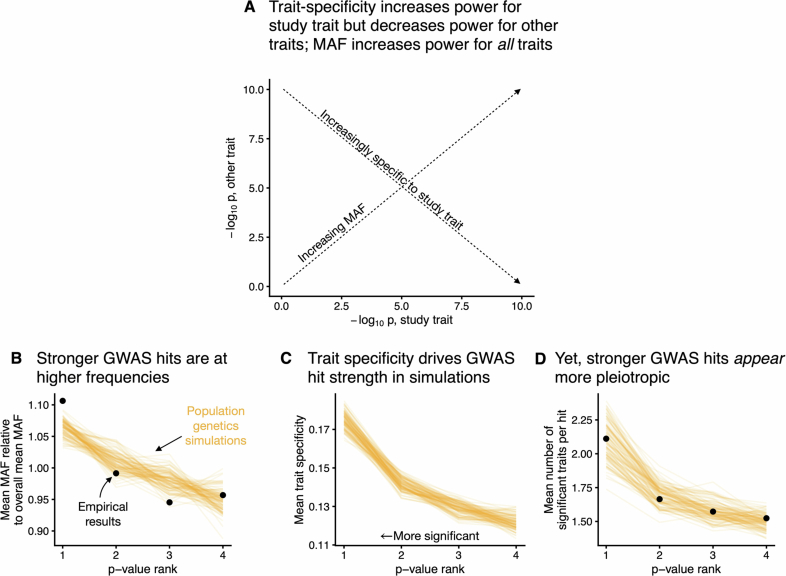
Genetic drift makes GWAS hits appear more pleiotropic. **A**) Schematic of the effects of minor allele frequency (MAF) and trait specificity on GWAS p-values. **B**) The relationship between MAF and p-value rank for $N=100$ simulations and real data from $N=\mathrm{18,879}$ uncorrelated genome-wide significant GWAS hits across genetically uncorrelated traits (Methods). Genome-wide significant hits were binned by p-value, and the mean MAF within each bin was compared to the overall mean MAF across all hits. Black points are results from UKB GWAS, and orange lines are simulations. This analysis was repeated for **C**) the mean trait specificity within each bin and **D**) the mean number of traits for which each hit was genome-wide significant. Panel **C** contains only simulations as the trait specificities for the UKB GWAS results are unknown.

This randomness in MAF drives in a counterintuitive result: variants that are the strongest hits for one trait are more likely to be hits for other traits, even though they are, on average, more trait specific (Extended Data Fig. 3; see Methods and Supplementary Appendix E for details). This reconciles our findings on the importance of trait specificity with previous studies that report GWAS hits appearing to be surprisingly pleiotropic^42–44^: the seemingly high pleiotropy at GWAS hits is a statistical artefact of the increased power at high-frequency variants.

## Estimating trait importance

We began by proposing that it could be desirable to prioritize genes either by trait importance or trait specificity. Yet, we have shown that when ranking by *P* value, neither LoF burden tests nor GWAS rank genes by importance. We wanted to see whether there was some way to use GWAS or LoF burden test results to prioritize genes in a way that is correlated with trait importance.

Throughout, we have focused on prioritizing genes based on *P* value or strength of association. It is natural to ask whether ranking genes on some other summary of the association tests, such as unbiased estimates of trait importance, would better prioritize important genes. In Supplementary Appendix H, we discuss why these simple approaches fail at ranking by trait importance.

To see whether more-complex uses of association test results might result in ranking genes by importance, we considered a simplified model where a variant has an effect $\beta$ on a gene that has effect $\gamma$ on the trait. This results in the overall effect of the variant on the trait being $\alpha =\beta \gamma$ (similar to the models in ref. ^45^). Extensions of our results to the case where $\alpha$ depends non-linearly on $\beta$ (as in ref. ^46^) and other caveats are discussed in Supplementary Appendix I.

**Fig. 5:**
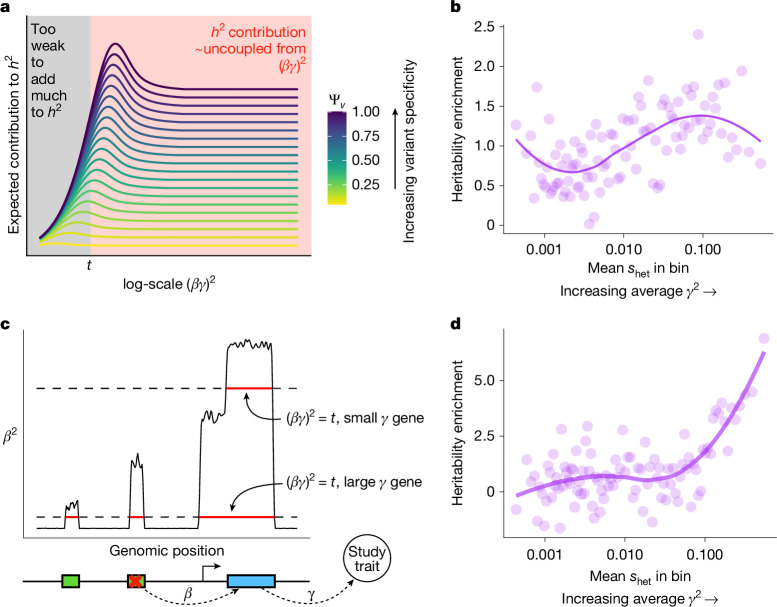
Estimating trait importance by combining different variant types. **a**, Theoretical expected contributions to heritability, ${h}^{2}$, as a function of the total effect of a variant on a trait ${\alpha }^{2}={(\beta \gamma )}^{2}$. The coloured lines are variants with different trait specificities. These functions can be approximately divided into a regime where variants contribute very little to heritability (black) or their contribution depends very little on ${(\beta \gamma )}^{2}$ (red). We denote the dividing line between these regimes by $t$. **b**, Enrichment of LoF burden test heritability for genes binned by ${s}_{\mathrm{het}}$. The plotted heritability enrichment is a normalized inverse variance-weighted average of heritability enrichments across 27 genetically uncorrelated traits (Methods). Each bin contains approximately 184 genes. The trend line was fit using LOESS. Pearson’s $r$ between heritability enrichment and ${s}_{\mathrm{het}}$ across the 25 highest *s*_het_ bins = −0.337, *P* = 0.099. **c**, Schematic of how the contribution of a variant to heritability depends on the ${\gamma }^{2}$ of the gene through which it acts. The green boxes represent *cis*-regulatory regions. **d**, Similar to panel **b** but estimated using GWAS results instead of LoF burden test results. Per-trait estimates were obtained using AMM^47^, and we plotted a normalized inverse variance-weighted average across the same traits as in panel **b** (Methods). Pearson’s $r$ between heritability enrichment and ${s}_{\mathrm{het}}$ across the 25 highest ${s}_{\mathrm{het}}$ bins = 0.832, *P* = 2.57 × 10^−7^.

As discussed above, estimating trait importance is most difficult for the most important genes due to flattening^20,33,40^. Flattening refers to the expected strength of association (equivalently, the expected contribution to heritability) first increasing as ${(\beta \gamma )}^{2}$ increases, but then becoming uncoupled from ${(\beta \gamma )}^{2}$ for sufficiently large ${(\beta \gamma )}^{2}$ (Fig. 5a and Supplementary Appendix F). This decoupling causes association studies to be incapable of prioritizing by trait importance, which we saw in LoF burden tests (Fig. 5b), in which contributions to heritability are poor predictors of ${s}_{\mathrm{het}}$, which we used there as a proxy for trait importance.

Yet, flattening does not affect all variants equally. For simplicity, imagine that variants either contribute minimally to heritability, or if their effect is greater than some threshold, $t$, then they contribute an amount independent of their importance (Fig. 5a). Now, imagine two genes: one has a large effect on the trait (large $\gamma$), and one has a small effect (small $\gamma$). Even variants that weakly perturb the large $\gamma$ gene will contribute to heritability, whereas for the small $\gamma$ gene, only variants with very large $\beta$ will contribute to heritability (Fig. 5c). Each individual variant experiences flattening, but collectively there will be more variants that contribute to heritability for more trait-important genes, all else being equal. As a result, the total heritability contributed by variants acting on a given gene should correlate with its trait imporance.

To test this, we used AMM^47^, which estimates the total heritability of variants acting via a given set of genes using GWAS data (Methods). We found that compared with LoF burden heritability, this measure of total heritability better tracks ${s}_{\mathrm{het}}$ and hence trait importance (Fig. 5d). The results of this analysis do not rely on using AMM: similar results hold whenever aggregating signals across variants with different *β* (for example, Supplementary Figs. 42, 49 and 50; Methods; see ref. ^48^ for an example using missense variants). Furthermore, this argument does not depend on the exact details of flattening. Our argument only requires that contributions to heritability generally increase with effect size but eventually plateau, which is sensible under any model where selection acts more strongly on larger effect variants^49^.

## Discussion

It is often stated that GWAS and burden tests converge on similar gene sets^19,21,50,51^. Indeed, some genes are implicated by both approaches, such as *LDLR* for low-density lipoprotein levels^20,52^. Generally, GWAS loci are enriched near burden genes and, conversely, burden genes — as well as genes identified in familial studies of Mendelian counterparts of the same traits — are enriched within GWAS loci^4,53^.

Here, we found that, despite this overall concordance, LoF burden tests and GWAS rank genes differently. Our analysis shows that LoF burden tests prioritize long, trait-specific genes, whereas GWAS prioritize genes near trait-specific variants that have drifted to unexpectedly high frequencies. Because context-specific variants can be trait specific even if they act on pleiotropic genes, GWAS can prioritize trait-relevant, pleiotropic genes, unlike LoF burden tests. This explains why burden tests often appear less polygenic than GWAS and tend to prioritize genes that are seemingly more directly related to trait biology^20^.

We found numerous GWAS loci with essentially no LoF burden signal, suggesting that context-specific variants acting on highly pleiotropic genes are major drivers of complex traits. We hypothesize that some of these genes have developmental roles, and these context-specific variants perturb developmental trajectories in a trait-specific manner^54^.

Although both study designs can only discover sufficiently trait-important genes, neither directly ranks genes by trait importance. LoF burden tests estimate trait importance, but selection causes estimation noise to increase with gene effect size, making rankings by significance nearly independent of trait importance. Gene length is also a major confounder. Although larger sample sizes will help to reduce noise, we anticipate that Bayesian frameworks using priors based on gene features^35,55^ could be particularly effective for improving the accuracy of burden tests.

In GWAS, genetic drift makes the *P* values of individual variants essentially arbitrary as long as the variants are sufficiently trait specific and important. This makes variant-level ranking of GWAS loci inefficient for identifying top genes. Instead, genes can be prioritized by trait importance using non-standard GWAS approaches that aggregate signals across multiple variants^47,48,56,57^, motivating further development of such methods.

Our findings also explain why GWAS results are highly effective for identifying trait-relevant tissues and cell types using approaches such as S-LDSC^9^. Variants that are only active in trait-relevant cell types are much more likely to be trait specific, and thus contribute more to heritability. This is not necessarily because such variants have larger effect sizes, but rather because all else being equal they are less constrained.

The question of how genes should ideally be prioritized is surprisingly understudied. Here we propose ranking genes based on either trait importance or trait specificity. Both concepts capture different aspects of what it means for a gene to be ‘relevant’ for a trait.

Which criterion should be used depends on the situation. For example, trait-specific genes may be better drug targets due to reduced side effects, perhaps explaining why LoF burden evidence is more predictive of drug trial success than GWAS evidence^58^. Yet, if pleiotropic genes can be targeted in a context-specific way, targeting the most trait-important genes may be more clinically impactful. In addition, the effects of pleiotropic genes in knockout experimental systems may differ fundamentally from the phenotypic consequences of regulatory variants identified in GWAS.

The fact that LoF burden tests and GWAS prioritize different genes is a blessing: both are useful, and both reveal different aspects of trait biology. However, it is important to understand what genes they prioritize and why. Our results make clear that both association study designs will be important in future efforts to map the genetic underpinnings of complex traits.

## Methods

### GWAS summary statistics

GWAS summary statistics for 305 continuous traits were downloaded from the Neale Lab (http://www.nealelab.is/uk-biobank/; v3). These regressions were run on inverse rank normal-transformed phenotypes in a subset of the UK Biobank consisting of approximately 360,000 individuals and included age, age^2^, inferred sex, age × inferred sex, age^2^ × inferred sex and principal components 1–20 as covariates. We used 5 × 10^−8^ as the threshold for genome-wide significance unless otherwise stated.

### LoF burden test summary statistics

Summary statistics for 292 LoF burden tests were downloaded from Backman et al.^4^. Two-hundred and nine traits overlapped with traits for which we had GWAS summary data (Supplementary Table 1). Burden genotypes were calculated by calling individuals homozygous for the non-LoF variant at all sites as being homozygous non-LoF, calling individuals homozygous for the LoF allele at any site as being homozygous LoF, and calling all other individuals heterozygotes. Burden tests were run using REGENIE^59^ on inverse rank normal-transformed phenotypes. For our primary analyses, we used the result of the burden test with mask M1, which only includes variants that are predicted as being LoFs using the most stringent filtering criteria and an allele frequency upper bound of 1%. For analyses including missense variants, we used mask M3, which also includes ‘likely damaging’ missense variants, again upper bounding the frequency of included variants at 1% (see ref. ^4^ for more details). We used a per-trait genome-wide significance threshold of 2.7 × 10^−6^, derived by applying a Bonferroni correction to a significance threshold of 0.05 for testing approximately 18,000 genes per trait.

### A subset of genetically uncorrelated traits

The set of 209 quantitative traits included some that were highly correlated, such as sitting height and standing height. For certain analyses, we selected a subset of 27 traits that were not highly correlated by intersecting the 209 traits with those analysed by Mostafavi et al.^45^ (Supplementary Table 1). In brief, the trait list was pruned to ensure that all pairwise genetic correlations, as reported by the Neale laboratory, were below 0.5, prioritizing traits with higher heritability. Biomarkers were excluded from this subset because their genetic correlations with other traits were not provided by the Neale laboratory. Genetic and phenotypic correlations between these 27 traits as reported by the Neale laboratory are listed in Supplementary Table 2. Genetic correlations ranged between −0.3096 and 0.2742. Phenotypic correlations for eight trait pairs were missing from the Neale laboratory (all including the trait ‘heel quantitative ultrasound index, direct entry’). The remaining phenotypic correlations ranged between −0.2117 and 0.1972.

We used this subset of traits to ensure that our results (Figs. 3b–d and 4b,c and Extended Data Figs. 1a–c and 3a–c) were not driven by many correlated phenotypes all sharing the same underlying biology. As such, slight correlations between these phenotypes should not substantively affect our results or interpretations.

### Defining GWAS loci

For a systematic comparison of discoveries between GWAS and burden tests (shown in Fig. 1c,d), we grouped GWAS variants into large, non-overlapping genomic loci. This approach avoids multiple counting of the same GWAS genes, as nearby hits within a locus may map to the same gene, and it provides a conservative estimate of the overlap between GWAS and burden test results as described below.

We focused on 151 quantitative traits with at least one burden test hit and one GWAS hit. For each trait, we analysed the set of LD-clumped hits ($P < 5\times {10}^{-8}$, clumping ${r}^{2} < 0.1$) from 8,136,100 filtered SNPs provided by Mostafavi et al.^45^. A secondary analysis (Supplementary Figs. 11–13) used the same LD-clumping pipeline but with a stricter threshold of ${r}^{2} < 0.01$.

For each trait, we began the grouping procedure with the most significant hit and iteratively processed all hits until they were assigned to a locus. For each hit, we included all independent hits with larger *P* values (lower significance) within 1 Mb to form a locus. The locus size was then expanded to ensure that no other hit was within 1 Mb of any variant already included in the locus. After completing one locus, we moved on to the next most significant hit that had not yet been assigned to any locus. Finally, we assigned overlapping genes to each locus, focusing on the 18,524 protein-coding genes analysed in the LoF burden test.

In Fig. 1c, we show the ranking of genome-wide significant genes from the burden test and the ranking of their corresponding GWAS loci, based on the *P* value of the most significant GWAS variant within each locus. In Fig. 1d, we plot the *P* value of the most significant GWAS variant within each locus on the *x* axis and the *P* value of the most significant gene from the burden test within the same locus on the *y* axis.

In a subset of analyses, we included only the top GWAS loci to match the statistical power of the burden test for gene discovery. We illustrate our procedure with the example of standing height. The LoF burden test for standing height identified 82 significant genes ($P < 2.7\times {10}^{-6}$, to account for the 18,524 genes tested). The GWAS analysis identified 3,374 nearly independent hits. Following the grouping procedure outlined above, these hits were consolidated into 382 loci (median size of 3.2 Mb). We ranked these loci by the minimum *P* value within each locus. Starting with the top-ranked locus, we iteratively added GWAS loci until we selected 82 genes. From each locus, we selected all genes that were significant in the LoF burden test. If no such genes existed, we selected the gene with the smallest burden test *P* value.

This procedure ensures that our analysis of the overlap between burden test and GWAS discoveries is conservative. The overestimation arises first from prioritizing genes based on burden test *P* values and second from using large GWAS loci, which may contain more than one causal gene, thereby increasing the likelihood of overlap with burden test results.

### Comparing GWAS and LoF burden tests in LD blocks

To avoid exacerbating dissimilarities between LoF burden tests and GWAS caused by mislocalization of GWAS signals, we also performed analyses at the LD block level. We downloaded bed files containing the coordinates of approximately independent LD blocks from ref. ^60^. For each trait, we computed the minimum GWAS *P* value of variants within each block and compared that with the minimum LoF burden test *P* value for all genes that overlapped any part of that block. In a small number of cases, the smallest LoF burden test *P* value in two adjacent blocks would be the same because a single highly significant gene overlapped both blocks. This generally reduced the correlation between the minimum *P* values of GWAS and LoF burden tests, and so we dropped all such blocks to be conservative.

### Ranking loci with conditionally independent hits

To evaluate the robustness of GWAS locus ranking, we also performed SNP selection using COJO^61^ instead of LD clumping. Starting from the set of 8,136,100 filtered SNPs (described above), we ran COJO for each trait using the --cojo-p 5e-8 and --cojo-slct options. The output is a set of conditionally independent SNPs along with their co-estimated effect sizes, which we used to define GWAS loci as described above for LD-clumped SNPs. Results based on this alternative approach are presented in Supplementary Figs. 8–10.

### Ranking genes in GWAS by MAGMA *P* value

We used MAGMA^57^ to obtain gene-level *P* values from GWAS data. We generally followed the suggestions from ref. ^62^. In brief, as recommended by PoPS^62^, we assigned SNPs to genes using magma --annotate with the default settings, which only uses SNPs inside of gene bodies. These were then combined using the 1000 Genomes EUR^63^ LD panel distributed with MAGMA to obtain gene-level *P* values using the --gene-model snp-wise=mean option.

### Ranking genes in GWAS by PoPS score

We also obtained gene-level scores using PoPS^62^. PoPS uses gene features to predict the gene-level scores produced by MAGMA. We used the MAGMA results as described above, and then downloaded approximately 40,000 features derived from gene expression datasets from GitHub (https://github.com/FinucaneLab/gene_features) as listed as the source of data in ref. ^62^. Additional features from protein–protein interaction networks and pathway membership are described in ref. ^62^, but only the expression features were available in the GitHub repository. Furthermore, there was a bug where features from different datasets had the same name causing PoPS to crash. We used a custom script to provide a unique name to each feature provided with PoPS. We then used PoPS with the default setting and used the output PoPS_Score from the resulting *.pred files to rank genes.

### Simulating GWAS in smaller samples

To simulate the results of performing smaller GWAS, we sampled summary statistics in a subsample of size $n$, ${\hat{\alpha }}^{\mathrm{sub}},$ conditioned on summary statistics in the full sample of size $N$, ${\hat{\alpha }}^{{\rm{full}}}$, as

1 $${\hat{\alpha }}^{\mathrm{sub}}{\rm{| }}{\hat{\alpha }}^{\mathrm{full}}\sim \mathrm{Normal}\,\left({\hat{\alpha }}^{\mathrm{full}},\frac{N-n}{n}{SE}{({\hat{\alpha }}^{\mathrm{full}})}^{2}\right)$$

and inflated the standard errors by a factor of $\sqrt{N/n}$. For a derivation see ref. ^64^, supplementary material section 3.2. We performed the sampling independently across 8,136,100 filtered SNPs (described above) that passed our filtering. This independent sampling alters the LD structure between linked SNPs, but as we use these statistics in analyses that depend only on the most significant SNP within a locus, this effect on LD should be inconsequential for our qualitative conclusions.

### Ranking GWAS loci using MAF thresholds

For Supplementary Figs. 26–28, we considered all 8,136,100 filtered SNPs (described above). We then filtered to only those SNPs equal to or below a given MAF threshold (0.01, 0.1 or 0.5, which includes all SNPs). We then constructed GWAS loci as described above and ranked loci by the minimum *P* value in that locus.

### Ranking loci by largest significant effect size

For Supplementary Figs. 29–31, we again considered all 8,136,100 filtered SNPs (described above). We then constructed GWAS loci as described above but ranked loci by taking the largest absolute effect size among the genome-wide significant SNPs in each locus. We ranked loci for burden tests by taking the largest absolute effect size among all genome-wide significant genes that overlapped the locus.

### Association study model

We combined population genetics and statistical genetics models to understand how natural selection affects variants based on their trait specificity and trait importance. Our model assumes that traits are under stabilizing selection based on prevailing hypotheses^36,38,39^ and uses standard population genetics theory^33,65–68^. The details of our model are outlined in the Supplementary Appendices.

### Unbiased estimates of trait importance

In several analyses we require estimates of trait importance, either ${\alpha }^{2}$ from GWAS or ${\gamma }^{2}$ from LoF burden tests. The details in both cases are identical, so here we describe ${\gamma }^{2}$. The naive estimator of squaring the LoF burden test estimated effect size, ${(\hat{\gamma })}^{2}$, is biased. Worse, this bias is anticorrelated with the frequency of the variant, which results in spurious correlations between the biased estimates and various gene properties such as ${s}_{\mathrm{het}}$.

To derive an unbiased estimator, we appealed to standard statistical genetics theory^69^ to assume that LoF burden estimates are approximately normally distributed about their true values with noise dependent on their standard errors. In particular, for a gene with standard error $s$ and effect size estimate $\hat{\gamma }$, we have that $\hat{\gamma }\sim \mathrm{Normal}(\gamma ,{s}^{2})$ approximately. This approximation is widely used for GWAS and was recently confirmed to be accurate for LoF burden tests^46^. It is then a routine calculation to check that ${(\hat{\gamma })}^{2}-{s}^{2}$ is an unbiased estimator of ${\gamma }^{2}$.

Although we focus exclusively on quantitative phenotypes in this study, we note that this approximation may not be valid for effect size estimates from logistic regression applied to case–control data.

### LoF burden summary statistics binned by ${{\boldsymbol{s}}}_{{\bf{het}}}$

When comparing LoF burden summary statistics (standard errors, ${z}^{2}$, and unbiased estimates of ${\gamma }^{2}$) to ${s}_{\mathrm{het}}$, we used ${s}_{\mathrm{het}}$ values inferred in ref. ^35^ and downloaded from ref. ^70^. We binned genes by ${s}_{\mathrm{het}}$ into 100 bins, each with approximately 184 genes. Within each bin, we averaged the respective summary statistics (for example, unbiased estimate of ${\gamma }^{2}$) across traits and genes. To make sure that our results were not driven by redundant traits, we used our 27 genetically uncorrelated traits for these analyses. For heritability enrichment (Fig. 5b), we used the fact that heritability should be proportional to ${z}^{2}-1$ (Supplementary Appendix B). Within each bin of genes, we then computed the average ${z}^{2}-1$ in that bin relative to the average of ${z}^{2}-1$ across all genes for each trait. This produced a trait-level enrichment for each bin, and by using the empirical standard deviation of the relative ${z}^{2}-1$ within the bin, we could also obtain an empirical standard error for the enrichment. We then obtained an overall enrichment for each bin, by taking an inverse-variance weighted average across traits. After this averaging, the mean heritability enrichment across genes need not be one. As such, we renormalized the estimates to average to one.

### ATAC peak specificity

We downloaded all ATAC-seq files from ChIP-Atlas^71^ that contained more than 5,000,000 mapped reads and identified at least 5,000 peaks. Across all files, overlapping peaks were combined using bedtools merge^72^. This yielded a total of 2,131,526 peaks. Samples other than blood samples were grouped into 17 tissues based on their annotations in ChIP-Atlas: adipocyte (146 samples), bone (190 samples), breast (815 samples), cardiovascular (559 samples), digestive (417 samples), epidermis (661 samples), gonad (138 samples), kidney (375 samples), liver (191 samples), lung (1,679 samples), muscle (118 samples), neural (1,349 samples), pancreas (322 samples), placenta (48 samples), pluripotent (1,895 samples), prostate (312 samples) and uterus (255 samples). In addition, samples with any of the following annotations were categorized as T cell (1,356 samples): CD4^+^ T lymphocytes, CD4^+^ T cells, CD8^+^ T lymphocytes, CD8^+^ T cells, fetal naive T cells, γδ T cells, naive T cells, T cells, chimeric antigen receptor T cells, follicular helper T cells, helper 0 T (T_H_0) cells, T_H_17 cells, T_H_1 cells, T_H_2 cells, T_H_9 cells or T lymphocytes. Samples with any of the following annotations were categorized as erythroid (102 samples): erythroid progenitors, erythroid cells or erythroblasts. Ultimately, this resulted in 19 tissues or cell-type categories.

A peak was considered to be present in a tissue if more than 5% of samples contained the peak. In downstream analyses, we used both the ‘number of shared tissues’ and ‘peak intensity’. We calculated the number of shared tissues by considering all peaks in the relevant tissue for a given trait (for example, bone for height) and then counting the number of tissues in which that peak was present. In particular, we only considered peaks that are present in the relevant tissue. We calculated peak intensity as the fraction of samples within the focal tissue that contain the peak.

### Gene expression specificity

We compiled estimates of gene expression in 17 tissue or cell types, which were intended to overlap with the categorization of ATAC-seq peaks when possible. All tissues that were ultimately matched to traits (see below) were included in both our ATAC-seq tissues and our expression tissues, but there are some differences between the remaining tissues. Average gene expression transcripts per million (TPM) of the following tissues were downloaded and extracted from the Human Protein Atlas^73^ tissue gene data (rna_tissue_hpa.tsv.zip): adipose tissue, breast, heart muscle, colon, skin, ovary, kidney, liver, lung, skeletal muscle, amygdala, pancreas, placenta and prostate. Average gene expression TPM of the following cell types were downloaded and extracted from the Human Protein Atlas single-cell type data (rna_single_cell_type.tsv.zip): erythroid cells and T cells. Average gene expression TPM of human bone samples was downloaded from the Gene Expression Omnibus^74^ accession GSE106292 (refs. ^75,76^).

In each tissue, genes with more than 10 TPM were considered to be ‘expressed’. We then restricted our analyses to genes expressed in the trait-relevant tissue. We computed an expression specificity score by taking the expression level in TPM in the trait-relevant tissue divided by the sum of expression levels across all 17 tissues. This provided an expression specificity score for every gene expressed in the trait-relevant cell type. For analyses involving expression specificity bins, we took all of these expression specificity scores across all nine trait–tissue pairs, computed quintiles and then assigned each gene for a given trait–tissue pair to its quintile.

### Linking traits to tissues

To identify which tissue (or cell type) is predominantly associated with a given trait, we ran S-LDSC^9,41^ to partition the heritability of all of our traits that had an estimated heritability of more than 0.04. We used annotations for 19 tissues and cell types constructed from our ATAC-seq analysis described above, along with the LDSC baseline v1.1 covariates. Our aim was to identify trait–tissue pairs in which heritability could clearly be explained by one tissue as opposed to multiple tissues. As such, we only retained traits that had a tissue with an LDSC *τ* with a *z*-score of more than 4.5 and had more than 40% of their heritability explained by variants in ATAC-seq peaks of the corresponding tissue. If more than one trait was assigned to the same tissue, we only kept genetically uncorrelated traits (${r}^{2} < 0.04$). This resulted in nine trait–tissue pairs (Supplementary Table 1): mean corpuscular volume (30040_irnt) → erythroid, reticulocyte percentage (30240_irnt) → erythroid, eosinophil percentage (30210_irnt) → T cell, lymphocyte count (30120_irnt) → T cell, standing height (50_irnt) → bone, heel bone mineral density (3148_irnt) → bone, glucose (30740_irnt) → pancreas, creatinine (30700_irnt) → liver, and alanine aminotransferase (30620_irnt) → liver.

### Regression of burden *z*^2^ on expression specificity

For each of the nine trait–tissue pairs described above, we performed a linear regression of the burden ${z}^{2}$ for all genes expressed in the top tissue on the expression specificity of genes, binned into quintiles as described earlier. We included the unbiased estimates of the trait importance of genes (defined above) as a covariate. For each specificity bin, we calculated an inverse-variance weighted average of the regression coefficients across all nine traits, with standard errors computed as the square root of the reciprocal of the total weight. The results, shown in Supplementary Fig. 36, demonstrate that the burden test prioritization of specifically expressed genes in Fig. 3e is not driven by differences in the importance of genes across specificity bins.

### S-LDSC analysis using ATAC-seq peaks

For each trait–tissue pair, we ran S-LDSC^9,41^ to estimate the heritability enrichment of tissue-specific ATAC-seq peaks. To this end, we categorized ATAC-seq peaks present in each tissue into five bins based on their presence in other tissues: present in 1–2 tissues, present in 3–8 tissues, present in 9–15 tissues, present in 16–18 tissues and present in all 19 tissues. Also, we categorized ATAC-seq peaks present in each tissue into five bins based on their intensity. The size of these bins were set to match the sizes of the tissue-specificity-based bins. We included the annotations based on ATAC peak tissue specificity and peak intensity bins with the LDSC baseline v1.1 model and used S-LDSC v.1.0.1 on HapMap3 SNPs^77^. In all analyses, we report $\tau /{h}^{2}$, which represents the change in the proportion of heritability explained by a single variant caused by toggling the annotation of that variant from 0 to 1 while keeping all other covariates included in the regression fixed.

### S-LDSC analysis using coding variants

We downloaded the variant annotation file (variants.tsv.bgz) from the Neale laboratory website (http://www.nealelab.is/uk-biobank/). We used the consequence information in the file, which corresponds to Ensembl Variant Effect Predictor (v85)^78^, for annotating variants. Specifically, we classified variants as being coding if their most severe consequence was any of:

- splice_donor_5th_base_variant
- missense_variant
- splice_region_variant
- splice_acceptor_variant
- splice_donor_variant
- splice_donor_region_variant
- stop_gained
- start_lost
- stop_lost
- frameshift_variant
- inframe_insertion
- protein_altering_variant

For each trait–tissue pair, we ran S-LDSC^9,41^ to estimate the heritability enrichment of coding variants as a function of expression specificity. We included the expression specificity bin (as defined above) as an annotation in the S-LDSC model. We also categorized genes into five equally sized bins based on their expression level in the tissue of interest, and all the coding variants were categorized into one of these five bins based on the expression level of the corresponding genes. These annotations were also included in the S-LDSC model. In addition, we used the covariates in the baseline v1.1 model and restricted our analysis to HapMap3 SNPs^77^. All analyses were run with S-LDSC v.1.0.1. As described above, we always report $\tau /{h}^{2}$.

### LoF burden summary statistics binned by ${\boldsymbol{\mu }}{\boldsymbol{L}}$

Analyses comparing LoF burden summary statistics to $\mu L$ were performed analogously to the analyses comparing the summary statistics to ${s}_{\mathrm{het}}$. As a proxy for *μL*, we downloaded the expected number of segregating LoFs for each gene as calculated in gnomAD (v2)^79^ from ref. ^70^. To show that $\mu L$ is essentially driven by coding DNA sequence (CDS) length, we downloaded CDS lengths for MANE select canonical transcripts (genome build GRCh38) from Ensmbl^80^ and correlated them with the expected number of segregating LoFs from gnomAD^79^ (Supplementary Fig. 44).

### Computing frequency spectra given ${{\boldsymbol{s}}}_{{\bf{het}}}$

To simulate under our model, we required the distribution of allele frequencies for a given selection coefficient. We assumed a stabilizing selection model, which is approximately equivalent to homozygotes having a relative fitness of 1 and heterozygotes having a fitness of 1-*s*_het_^33,65–67^. We used fastDTWF^81^ to compute likelihoods under this model. We assumed an equilibrium population of 20,000 diploids, and computed allele frequency distributions along a grid of 50 *s*_het_ values from 10^−7^ to 0.05 evenly spaced on the log scale. We used 1.25 × 10^−8^ as the per-generation mutation rate. We considered a model where the ancestral allele is known by using the no_fix=True option in fastDTWF. In addition, fastDTWF has two parameters that control the accuracy of its approximation. On the basis of the recommendations of ref. ^81^, we set dtwf_tv_sd to 0.1 and dtwf_row_eps to 10^−8^.

### Simulating realized heritability

To generate Extended Data Fig. 2b, we simulated 50,000 unlinked variants from our stabilizing selection model. We considered 1,000 values of ${s}_{\mathrm{het}}$ log-uniformly spaced between 10^−7^ and 2.3 × 10^−4^. For each value of ${s}_{\mathrm{het}}$, we then simulated 50 variants by drawing 50 allele frequencies from the allele frequency distributions that we computed as described above. To model the slight differences between population and GWAS sample allele frequencies, we then drew a GWAS sample allele count for each variant as a $\mathrm{Binomial}(\mathrm{600,000},f)$ random variable, where $f$ was the population frequency, and 600,000 was chosen to match the roughly 300,000 diploids in the UK Biobank. These allele counts were then normalized to obtain GWAS sample allele frequencies, $\widetilde{f}$. For this simulation, we assumed that all variants have the same trait specificity. This makes ${\alpha }^{2}$ on the focal trait proportional to ${s}_{\mathrm{het}}$, so we set the realized heritability to $2{s}_{\mathrm{het}}\,\widetilde{f}\,(1-\widetilde{f})$, and normalized all results relative to the maximum simulated realized heritability. Likewise, effect sizes were reported relative to the maximum simulated effect size.

### Computing pleiotropy of GWAS hits

To investigate the pleiotropy of top versus weak GWAS hits, we considered all of the 27 uncorrelated traits that had at least 100 GWAS hits, leaving 18 traits. For each trait, we grouped the hits into four quartiles based on variant *P* values, with quartile 1 containing the most statistically significant hits and quartile 4 containing the least. For each hit, we calculated the number of traits (out of 18) in which the variant was a hit and computed the mean values within each quartile.

### Simulating pleiotropy of GWAS hits

To simulate the effects of genetic drift on the apparent pleiotropy of GWAS hits, we simulated GWAS summary statistics. To match the real data described above, we considered 18 traits and simulated effect sizes for 10 million not necessarily segregating positions. We simulated the effect sizes independently for each position, and drew the vector of squared effect sizes for variant *j*,${\vec{{\alpha }^{2}}}_{j}\in {{\mathbb{R}}}^{18}$ as

$${\vec{{\alpha }^{2}}}_{j}\sim \frac{{10}^{-7}}{f}\times \exp \{3f\times \mathrm{Normal}({\bf{0}},p{\bf{I}}+(1-p){{\bf{11}}}^{T})\}$$

where the exponentiation is performed element-wise, and $f$ and $p$ are parameters that affect the range of different total effect sizes, ${{||}{\vec{{\alpha }^{2}}}_{j}{||}}_{1}$, and distribution of trait specificities.

We then assumed that the strength of selection against the variant was ${{||}{\vec{{\alpha }^{2}}}_{j}{||}}_{1}$. We obtained the MAF for each variant by drawing from the variant frequency distribution with the closest ${s}_{\mathrm{het}}$, computed as described above.

Finally, we simulated a GWAS by assuming that the observed association statistic for each trait was independently normally distributed about its true value. For example, for trait $k$ and the variant at position $j$ we have:

$${\hat{\alpha }}_{{jk}}\sim \mathrm{Normal}\,\left(\sqrt{{\vec{{\alpha }^{2}}}_{{jk}}},\frac{1}{\sqrt{2{N}_{\mathrm{eff}}\times \mathrm{MA}{{\rm{F}}}_{j}(1-\mathrm{MA}{{\rm{F}}}_{j}))}}\right)$$

where ${N}_{{\rm{eff}}}$ is a scaling factor that captures both the amount of environmental noise contributing to the trait as well as the sample size. We converted these to *P* values by taking $2{N}_{{\rm{eff}}}{{\rm{MAF}}}_{j}(1-{{\rm{MAF}}}_{j}){\hat{\alpha }}_{{jk}}^{2}$ as a squared *z*-score, which is chi-squared distributed with 1 degree of freedom under the null. We considered a variant to be a genome-wide significant hit if its *P* value was smaller than a parameter, $t$.

This simulation approach has four free parameters. In the main text, we used $f=0.33$, $p=0.5$, ${N}_{\mathrm{eff}}=10000000$ and $t={10}^{-5}$. Although these parameters are related to standard GWAS parameters (for example, the GWAS sample size or genome-wide significance threshold), the exact quantitative relationship should not be overanalysed. For example, we assumed that the strength of selection is exactly ${{||}{\vec{{\alpha }^{2}}}_{j}{||}}_{1}$. If instead there was some scaling factor, that could be absorbed into ${N}_{{\rm{eff}}}$. Similarly, there is a qualitative inverse relationship between the effects of $t$ and ${N}_{{\rm{eff}}}$ (for example, lower $t$ has a similar effect to increasing ${N}_{{\rm{eff}}}$), making the exact setting of either parameter somewhat arbitrary. We chose the values that we used here to roughly match the distribution of selection coefficients inferred from real GWAS data^82^, as well as the observed patterns of MAF and pleiotropy in the UK Biobank GWAS results. In Supplementary Figs. 45–48, we vary each of ${N}_{{\rm{eff}}}$, $t$, $p$ and $f$, respectively, while holding the others fixed to show that our qualitative results are not sensitive to the particular simulation parameters that we chose.

### AMM analysis

We ran AMM^47^ to estimate heritability enrichments for gene sets, following the workflow previously described (https://github.com/danjweiner/AMM21; commit 524c620). We binned genes into 100 approximately equally sized bins based on ${s}_{\mathrm{het}}$ as described above and used these bins as our gene sets. AMM requires an estimate of the probability that a SNP is acting via the closest gene, second closest gene, and so on. For more distant genes, there is insufficient power to estimate these probabilities so AMM recommends combining these into bins. We followed the recommended binning, and then used the probabilities estimated in the original AMM paper^47^ (supplementary table 5 of ref. ^47^). AMM recommends using LDSC baseline covariates in all models, for which we used v2.3. We restricted our analysis to HapMap3 variants. The results in Fig. 5d are the inverse-variance weighted average of the heritability enrichment estimates across our 27 genetically uncorrelated traits. These inverse-variance weighted estimates of the average enrichments do not necessarily need to average to one in contrast to the true enrichments. As such, we renormalized the estimated enrichments so that they sum to one.

### Correlation of GWAS hit probability and ${{\boldsymbol{s}}}_{{\bf{het}}}$

We analysed the GWAS hits curated in our previous study^45^, filtered to a set of 6,971,256 SNPs that passed quality control procedures. This set excluded lead GWAS SNPs in LD (${r}^{2} > 0.8$) with variants predicted to have protein-altering consequences, to condition on putatively non-coding trait associations. We focused on 15,591 approximately independent GWAS hits associated with our 27 uncorrelated traits, for which estimates of *s*_het_ for the nearest gene were available. We performed logistic regression to differentiate GWAS hits from 100,000 SNPs randomly sampled from the same 6,971,256 SNP set. The *s*_het_ values of the nearest genes were used as the predictor, categorized into 100 percentile bins. As in our previous work, the regression model included additional covariates: MAF, LD score, gene density and the absolute distance to the nearest transcription start site. We also incorporated dummy variables representing 20 quantiles of each of these covariates (MAF, LD score, gene density and distance to the transcription start site). Results of this analysis are presented in Supplementary Fig. 49. The covariate data were obtained from Mostafavi et al.^45^.

### Correlation of ${\hat{{\boldsymbol{\gamma }}}}^{{\bf{2}}}$ and number of GWAS hits

To avoid double counting GWAS hits due to LD, we restricted our analysis to approximately independent hits. For each trait, we analysed the set of LD-clumped hits ($P < 5\times {10}^{-8}$, clumping ${r}^{2} < 0.1$) from 8,136,100 filtered SNPs provided in ref. ^45^. We then assigned each GWAS hit to the closest gene (using the midpoint of genes as released with AMM^47^). For each trait, we then correlated the number of GWAS hits assigned to each gene with our unbiased estimate of the trait importance of that gene, ${\hat{\gamma }}^{2}$, based on the LoF burden test results. To make certain that our results were not driven by differences between genes with no GWAS signal versus genes with any GWAS signal, we also computed correlations between the number of GWAS hits assigned to each gene and ${\hat{\gamma }}^{2}$ restricting to genes with at least one GWAS hit.

### Reporting summary

Further information on research design is available in the Nature Portfolio Reporting Summary linked to this article.

## Online content

Any methods, additional references, Nature Portfolio reporting summaries, source data, extended data, supplementary information, acknowledgements, peer review information; details of author contributions and competing interests; and statements of data and code availability are available at 10.1038/s41586-025-09703-7.

## Supplementary information

Supplementary Information This file contains all Supplementary Figures, descriptions of the Supplementary Tables, and the Appendices. The document begins with a table of contents providing the location of each figure, table description, and appendix in the document. The Appendices contain mathematical details and secondary analyses that were performed as robustness checks.

Reporting Summary

Supplementary Table 1 **List of traits and abbreviations used in the study**. A table in CSV format of the 209 traits used in this study with the UKB trait IDs, trait names, abbreviations used, tissue to which each trait was linked (if applicable), and an indication of whether or not the trait was included in our subset of 27 genetically uncorrelated traits (Methods)

Supplementary Table 2 **Genetic and phenotypic correlations between 27 “genetically uncorrelated traits”**. A table in CSV format of the genetic correlation ${r}_{g}$ and phenotypic correlation between each pair of traits among the 27 genetically uncorrelated traits used throughout the paper. Phenotype IDs match those listed in Supplementary Table 1. Estimates were obtained from the Neale Lab (Methods), and phenotypic correlations are missing for 8 of 351 trait pairs

## Data Availability

All data for reproducing the figures are provided on GitHub (https://github.com/jeffspence/specificity_length_luck).

## Code availability

The scripts for reproducing the figures and simulations are provided on GitHub (https://github.com/jeffspence/specificity_length_luck).

## References

[1] Claussnitzer, M. et al. A brief history of human disease genetics. *Nature* **577**, 179–189 (2020). 31915397 10.1038/s41586-019-1879-7 PMC7405896

[2] Bycroft, C. et al. The UK Biobank resource with deep phenotyping and genomic data. *Nature* **562**, 203–209 (2018). 30305743 10.1038/s41586-018-0579-z PMC6786975

[3] Szustakowski, J. D. et al. Advancing human genetics research and drug discovery through exome sequencing of the UK Biobank. *Nat. Genet.* **53**, 942–948 (2021). 34183854 10.1038/s41588-021-00885-0

[4] Backman, J. D. et al. Exome sequencing and analysis of 454,787 UK Biobank participants. *Nature* **599**, 628–634 (2021). 34662886 10.1038/s41586-021-04103-z PMC8596853

[5] Dickinson, M. E. et al. High-throughput discovery of novel developmental phenotypes. *Nature* **537**, 508–514 (2016). 27626380 10.1038/nature19356 PMC5295821

[6] Amin, N. D. & Paşca, S. P. Building models of brain disorders with three-dimensional organoids. *Neuron* **100**, 389–405 (2018). 30359604 10.1016/j.neuron.2018.10.007

[7] Replogle, J. M. et al. Mapping information-rich genotype–phenotype landscapes with genome-scale Perturb-seq. *Cell* **185**, 2559–2575 (2022). 35688146 10.1016/j.cell.2022.05.013 PMC9380471

[8] Minikel, E. V. et al. Refining the impact of genetic evidence on clinical success. *Nature*10.1038/s41586-024-07316-0 (2024). 10.1038/s41586-024-07316-0 PMC11096124 38632401

[9] Finucane, H. K. et al. Partitioning heritability by functional annotation using genome-wide association summary statistics. *Nat. Genet.* **47**, 1228–1235 (2015). 26414678 10.1038/ng.3404 PMC4626285

[10] Calderon, D. et al. Inferring relevant cell types for complex traits by using single-cell gene expression. *Am. J. Hum. Genet.* **101**, 686–699 (2017). 29106824 10.1016/j.ajhg.2017.09.009 PMC5673624

[11] Richard, D. et al. Functional genomics of human skeletal development and the patterning of height heritability. *Cell*10.1016/j.cell.2024.10.040 (2024). 10.1016/j.cell.2024.10.040 PMC11724752 39549696

[12] Schnitzler, G. R. et al. Convergence of coronary artery disease genes onto endothelial cell programs. *Nature* **626**, 799–807 (2024). 38326615 10.1038/s41586-024-07022-x PMC10921916

[13] Maurano, M. T. et al. Systematic localization of common disease-associated variation in regulatory DNA. *Science* **337**, 1190–1195 (2012). 22955828 10.1126/science.1222794 PMC3771521

[14] Boyle, E. A., Li, Y. I. & Pritchard, J. K. An expanded view of complex traits: from polygenic to omnigenic. *Cell* **169**, 1177–1186 (2017). 28622505 10.1016/j.cell.2017.05.038 PMC5536862

[15] Sinnott-Armstrong, N. et al. GWAS of three molecular traits highlights core genes and pathways alongside a highly polygenic background. *eLife* **10**, e58615 (2021). 33587031 10.7554/eLife.58615 PMC7884075

[16] Yengo, L. et al. A saturated map of common genetic variants associated with human height. *Nature* **610**, 704–712 (2022). 36224396 10.1038/s41586-022-05275-y PMC9605867

[17] Morgenthaler, S. & Thilly, W. G. A strategy to discover genes that carry multi-allelic or mono-allelic risk for common diseases: a cohort allelic sums test (CAST). *Mut. Res.* **615**, 28–56 (2007). 17101154 10.1016/j.mrfmmm.2006.09.003

[18] Singh, T. et al. Rare coding variants in ten genes confer substantial risk for schizophrenia. *Nature* **604**, 509–516 (2022). 35396579 10.1038/s41586-022-04556-w PMC9805802

[19] Zhou, D. et al. A phenome-wide scan reveals convergence of common and rare variant associations. *Genome Med.* **15**, 101 (2023). 38017547 10.1186/s13073-023-01253-9 PMC10683189

[20] Weiner, D. J. et al. Polygenic architecture of rare coding variation across 394,783 exomes. *Nature* **614**, 492–499 (2023). 36755099 10.1038/s41586-022-05684-z PMC10614218

[21] Hawkes, G. et al. Whole-genome sequencing analysis of anthropometric traits in 672,976 individuals reveals convergence between rare and common genetic associations. Preprint at *bioRxiv*10.1101/2025.02.24.639925 (2025). 10.1038/s41467-026-69208-3 PMC12987921 41651857

[22] Tsuji, T. & Kunieda, T. A loss-of-function mutation in natriuretic peptide receptor 2 (*Npr2*) gene is responsible for disproportionate dwarfism in *cn*/*cn* mouse. *J. Biol. Chem.* **280**, 14288–14292 (2005). 15722353 10.1074/jbc.C500024200

[23] Olney, R. C. et al. Heterozygous mutations in natriuretic peptide receptor-B (*NPR2*) are associated with short stature. *J. Clin. Endocrinol. Metab.* **91**, 1229–1232 (2006). 16384845 10.1210/jc.2005-1949

[24] Vasques, G. A. et al. Heterozygous mutations in natriuretic peptide receptor-B (*NPR2*) gene as a cause of short stature in patients initially classified as idiopathic short stature. *J. Clin. Endocrinol. Metab.* **98**, E1636–E1644 (2013). 24001744 10.1210/jc.2013-2142

[25] Amano, N. et al. Identification and functional characterization of two novel *NPR2* mutations in Japanese patients with short stature. *J. Clin. Endocrinol. Metab.* **99**, E713–E718 (2014). 24471569 10.1210/jc.2013-3525

[26] Wang, S. R. et al. Heterozygous mutations in natriuretic peptide receptor-B (*NPR2*) gene as a cause of short stature. *Hum. Mutat.* **36**, 474–481 (2015). 25703509 10.1002/humu.22773 PMC4382411

[27] Amberger, J. S. et al. OMIM. org: Online Mendelian Inheritance in Man (OMIM®), an online catalog of human genes and genetic disorders. *Nucleic Acids Res.* **43**, D789–D798 (2015). 25428349 10.1093/nar/gku1205 PMC4383985

[28] Conery, M. & Grant, S. F. A. Human height: a model common complex trait. *Ann. Hum. Biol.* **50**, 258–266 (2023). 37343163 10.1080/03014460.2023.2215546 PMC10368389

[29] To, K. et al. A multi-omic atlas of human embryonic skeletal development. *Nature* **635**, 657–667 (2024). 39567793 10.1038/s41586-024-08189-z PMC11578895

[30] Chuang, P.-T. & McMahon, A. P. Vertebrate Hedgehog signalling modulated by induction of a Hedgehog-binding protein. *Nature* **397**, 617–621 (1999). 10050855 10.1038/17611

[31] Bishop, B. et al. Structural insights into Hedgehog ligand sequestration by the human Hedgehog-interacting protein HHIP. *Nat. Struct. Mol. Biol.* **16**, 698–703 (2009). 19561611 10.1038/nsmb.1607 PMC2709225

[32] Briscoe, J. & Thérond, P. P. The mechanisms of Hedgehog signalling and its roles in development and disease. *Nat. Rev. Mol. Cell Biol.* **14**, 416–429 (2013). 23719536 10.1038/nrm3598

[33] Simons, Y. B. et al. A population genetic interpretation of GWAS findings for human quantitative traits. *PLoS Biol.* **16**, e2002985 (2018). 29547617 10.1371/journal.pbio.2002985 PMC5871013

[34] Gillespie, J. H. *Population Genetics: a Concise Guide* (JHU Press, 2004).

[35] Zeng, T. et al. Bayesian estimation of gene constraint from an evolutionary model with gene features. *Nat. Genet.* **56**, 1632–1643 (2024). 38977852 10.1038/s41588-024-01820-9

[36] Sanjak, J. S. et al. Evidence of directional and stabilizing selection in contemporary humans. *Proc. Natl Acad. Sci. USA* **115**, 151–156 (2018). 29255044 10.1073/pnas.1707227114 PMC5776788

[37] Sella, G. & Barton, N. H. Thinking about the evolution of complex traits in the era of genome-wide association studies. *Annu. Rev. Genomics Hum. Genet.* **20**, 461–493 (2019). 31283361 10.1146/annurev-genom-083115-022316

[38] Patel, R. A. et al. Characterizing selection on complex traits through conditional frequency spectra. *Genetics* **229**, iyae210 (2025). 39691067 10.1093/genetics/iyae210 PMC12005249

[39] Koch, E. et al. Genetic association data are broadly consistent with stabilizing selection shaping human common diseases and traits. Preprint at *bioRxiv*10.1101/2024.06.19.599789 (2024).

[40] O’Connor, L. J. et al. Extreme polygenicity of complex traits is explained by negative selection. *Am. J. Hum. Genet.* **105**, 456–476 (2019). 31402091 10.1016/j.ajhg.2019.07.003 PMC6732528

[41] Gazal, S. et al. Linkage disequilibrium-dependent architecture of human complex traits shows action of negative selection. *Nat. Genet.* **49**, 1421–1427 (2017). 28892061 10.1038/ng.3954 PMC6133304

[42] Pickrell, J. K. et al. Detection and interpretation of shared genetic influences on 42 human traits. *Nat. Genet.* **48**, 709–717 (2016). 27182965 10.1038/ng.3570 PMC5207801

[43] Watanabe, K. et al. A global overview of pleiotropy and genetic architecture in complex traits. *Nat. Genet.* **51**, 1339–1348 (2019). 31427789 10.1038/s41588-019-0481-0

[44] Qi, G. et al. Genome-wide large-scale multi-trait analysis characterizes global patterns of pleiotropy and unique trait-specific variants. *Nat. Commun.* **15**, 6985 (2024). 39143063 10.1038/s41467-024-51075-5 PMC11324957

[45] Mostafavi, H. et al. Systematic differences in discovery of genetic effects on gene expression and complex traits. *Nat. Genet.* **55**, 1866–1875 (2023). 37857933 10.1038/s41588-023-01529-1 PMC12270542

[46] Milind, N. et al. Buffering and non-monotonic behavior of gene dosage response curves for human complex traits. Preprint at *medRxiv*10.1101/2024.11.11.24317065 (2024).

[47] Weiner, D. J. et al. Partitioning gene-mediated disease heritability without eQTLs. *Am. J. Hum. Genet.* **109**, 405–416 (2022). 35143757 10.1016/j.ajhg.2022.01.010 PMC8948166

[48] Pathan, N. et al. A method to estimate the contribution of rare coding variants to complex trait heritability. *Nat. Commun.* **15**, 1245 (2024). 38336875 10.1038/s41467-024-45407-8 PMC10858280

[49] Schoech, A. P. et al. Quantification of frequency-dependent genetic architectures in 25 UK Biobank traits reveals action of negative selection. *Nat. Commun.* **10**, 790 (2019). 30770844 10.1038/s41467-019-08424-6 PMC6377669

[50] Lappalainen, T. et al. Genetic and molecular architecture of complex traits. *Cell* **187**, 1059–1075 (2024). 38428388 10.1016/j.cell.2024.01.023 PMC10977002

[51] Akbari, P. et al. Sequencing of 640,000 exomes identifies *GPR75* variants associated with protection from obesity. *Science* **373**, eabf8683 (2021). 34210852 10.1126/science.abf8683 PMC10275396

[52] Gazal, S. et al. Combining SNP-to-gene linking strategies to identify disease genes and assess disease omnigenicity. *Nat. Genet.* **54**, 827–836 (2022). 35668300 10.1038/s41588-022-01087-y PMC9894581

[53] Freund, M. K. et al. Phenotype-specific enrichment of Mendelian disorder genes near GWAS regions across 62 complex traits. *Am. J. Hum. Genet.* **103**, 535–552 (2018). 30290150 10.1016/j.ajhg.2018.08.017 PMC6174356

[54] Umans, B. D., Battle, A. & Gilad, Y. Where are the disease-associated eQTLs? *Trends Genet.* **37**, 109–124 (2021). 32912663 10.1016/j.tig.2020.08.009 PMC8162831

[55] Ota, M. et al. Causal modeling of gene effects from regulators to programs to traits: integration of genetic associations and Perturb-seq. Preprint at *bioRxiv*10.1101/2025.01.22.634424 (2025).

[56] Burch, K. S. et al. Partitioning gene-level contributions to complex-trait heritability by allele frequency identifies disease-relevant genes. *Am. J. Hum. Genet.* **109**, 692–709 (2022). 35271803 10.1016/j.ajhg.2022.02.012 PMC9069080

[57] de Leeuw, C. A. et al. MAGMA: generalized gene-set analysis of GWAS data. *PLoS Comput. Biol.* **11**, e1004219 (2015). 25885710 10.1371/journal.pcbi.1004219 PMC4401657

[58] Duffy, Á et al. Development of a human genetics-guided priority score for 19,365 genes and 399 drug indications. *Nat. Genet.* **56**, 51–59 (2024). 38172303 10.1038/s41588-023-01609-2 PMC11776516

[59] Mbatchou, J. et al. Computationally efficient whole-genome regression for quantitative and binary traits. *Nat. Genet.* **53**, 1097–1103 (2021). 34017140 10.1038/s41588-021-00870-7

[60] Berisa, T. & Pickrell, J. K. Approximately independent linkage disequilibrium blocks in human populations. *Bioinformatics* **32**, 283–285 (2016). 26395773 10.1093/bioinformatics/btv546 PMC4731402

[61] Yang, J. et al. Conditional and joint multiple-SNP analysis of GWAS summary statistics identifies additional variants influencing complex traits. *Nat. Genet.* **44**, 369–375 (2012). 22426310 10.1038/ng.2213 PMC3593158

[62] Weeks, E. M. et al. Leveraging polygenic enrichments of gene features to predict genes underlying complex traits and diseases. *Nat. Genet.* **55**, 1267–1276 (2023). 37443254 10.1038/s41588-023-01443-6 PMC10836580

[63] 1000 Genomes Project Consortium. A global reference for human genetic variation. *Nature* **526**, 68–74 (2015). 26432245 10.1038/nature15393 PMC4750478

[64] Miao, J. et al. Quantifying portable genetic effects and improving cross-ancestry genetic prediction with GWAS summary statistics. *Nat. Commun.* **14**, 832 (2023). 36788230 10.1038/s41467-023-36544-7 PMC9929290

[65] Bulmer, M. G. The genetic variability of polygenic characters under optimizing selection, mutation and drift. *Genet. Res.* **19**, 17–25 (1972). 5024710 10.1017/s0016672300014221

[66] Bulmer, M. G. Linkage disequilibrium and genetic variability. *Genet. Res.* **23**, 281–289 (1974). 4435356 10.1017/s0016672300014920

[67] Keightley, P. D. & Hill, W. G. Quantitative genetic variability maintained by mutation-stabilizing selection balance in finite populations. *Genet. Res.* **52**, 33–43 (1988). 3181758 10.1017/s0016672300027282

[68] Negm, S. & Veller, C. The effect of long-range linkage disequilibrium on allele-frequency dynamics under stabilizing selection. Preprint at *bioRxiv*10.1101/2024.06.27.601075 (2024). 10.1371/journal.pgen.1012035 PMC12991805 41801963

[69] Zhu, X. & Stephens, M. Bayesian large-scale multiple regression with summary statistics from genome-wide association studies. *Ann. Appl. Stat.* **11**, 1561–1592 (2017). 29399241 10.1214/17-aoas1046 PMC5796536

[70] Zeng, T. et al. *s*_het estimates from GeneBayes and other supplementary datasets. *Zenodo*10.5281/zenodo.7939767 (2023).

[71] Zou, Z., Ohta, T. & Oki, S. ChIP-Atlas 3.0: a data-mining suite to explore chromosome architecture together with large-scale regulome data. *Nucleic Acids Res.* **52**, gkae358 (2024). 10.1093/nar/gkae358 PMC11223792 38749504

[72] Quinlan, A. R. & Hall, I. M. BEDTools: a flexible suite of utilities for comparing genomic features. *Bioinformatics* **26**, 841–842 (2010). 20110278 10.1093/bioinformatics/btq033 PMC2832824

[73] Uhlén, M. et al. Tissue-based map of the human proteome. *Science* **347**, 1260419 (2015). 25613900 10.1126/science.1260419

[74] Barrett, T. et al. NCBI GEO: archive for functional genomics data sets — update. *Nucleic Acids Res.* **41**, D991–D995 (2012). 23193258 10.1093/nar/gks1193 PMC3531084

[75] Hicks, M. R. et al. ERBB3 and NGFR mark a distinct skeletal muscle progenitor cell in human development and hPSCs. *Nat. Cell Biol.* **20**, 46–57 (2018). 29255171 10.1038/s41556-017-0010-2 PMC5962356

[76] Ferguson, G. B. et al. Mapping molecular landmarks of human skeletal ontogeny and pluripotent stem cell-derived articular chondrocytes. *Nat. Commun.* **9**, 3634 (2018). 30194383 10.1038/s41467-018-05573-y PMC6128860

[77] International HapMap 3 Consortium. Integrating common and rare genetic variation in diverse human populations. *Nature* **467**, 52–58 (2010). 20811451 10.1038/nature09298 PMC3173859

[78] McLaren, W. et al. The Ensembl variant effect predictor. *Genome Biol.* **17**, 122 (2016). 27268795 10.1186/s13059-016-0974-4 PMC4893825

[79] Karczewski, K. J. et al. The mutational constraint spectrum quantified from variation in 141,456 humans. *Nature* **581**, 434–443 (2020). 32461654 10.1038/s41586-020-2308-7 PMC7334197

[80] Morales, J. et al. A joint NCBI and EMBL-EBI transcript set for clinical genomics and research. *Nature* **604**, 310–315 (2022). 35388217 10.1038/s41586-022-04558-8 PMC9007741

[81] Spence, J. P. et al. Scaling the discrete-time Wright–Fisher model to biobank-scale datasets. *Genetics* **225**, iyad168 (2023). 37724741 10.1093/genetics/iyad168 PMC10627256

[82] Simons, Y. B. et al. Simple scaling laws control the genetic architectures of human complex traits. *PLoS Biol.* **23**, e3003402 (2025). 10.1371/journal.pbio.3003402 PMC12517483 41082512

