# Supplementary material for: Specificity, length and luck drive gene rankings in association studies
Source: Nature. Author manuscript; Available in PMC 2026 Feb 5. (PMC12823407; doi:10.1038/s41586-025-09703-7)
Supplement: Supplementary Information [file NIHMS2128399-supplement-Supplementary_Information.pdf]

nature portfolio

https://doi.org/10.1038/s41586-025-09703-7

Supplementary information

Specificity, length and luck drive gene rankings in association studies

In the format provided by the authors and unedited

Supplementary materials

Contents

Supplementary Figures **4**

S1 Limited GWAS overlap at top LoF burden hits. 4

S2 Strongest GWAS hits are not always strong LoF burden hits. 5

S3 Modest correlation between GWAS and LoF burden test p-value ranks. 6

S4 Limited GWAS overlap at top LoF burden LD blocks. 7

S5 GWAS and LoF burden tests prioritize different LD blocks for height. 8

S6 GWAS and LoF burden tests prioritize different LD blocks across traits. 9

S7 Modest correlation between GWAS and LoF burden test p-value ranks across LD blocks. 10

S8 Top burden genes are ranked very differently by GWAS when using COJO SNPs. . . 11

S9 GWAS and LoF burden tests prioritize different GWAS loci for height when using COJO SNPs. 12

S10 GWAS and LoF burden tests prioritize different GWAS loci across traits when using COJO SNPs. 13

S11 Top burden genes are ranked very differently by GWAS when using more aggressively LD-clumped SNPs. 14

S12 GWAS and LoF burden tests prioritize different GWAS loci for height when using more aggressively LD-clumped SNPs. 15

S13 GWAS and LoF burden tests prioritize different GWAS loci across traits when using more aggressively LD-clumped SNPs. 16

S14 Top burden genes are ranked very differently by GWAS when ranking using MAGMA. 17

S15 GWAS and LoF burden tests prioritize different genes for height when ranking genes in GWAS using MAGMA. 18

S16 GWAS and LoF burden tests prioritize different genes when ranking genes in GWAS using MAGMA. 19

S17 Top burden genes are ranked very differently by GWAS when ranking using PoPS. . 20

S18 GWAS and LoF burden tests prioritize different genes for height when ranking genes in GWAS using PoPS. 21
S19 GWAS and LoF burden tests prioritize different genes when ranking genes in GWAS using PoPS. 22
S20 Top burden genes are ranked very differently by GWAS regardless of GWAS sample size. 23
S21 GWAS and LoF burden tests prioritize different genes for height regardless of GWAS sample size. 24
S22 GWAS and LoF burden tests prioritize different genes regardless of GWAS sample size. 25
S23 Top LoF plus damaging missense burden genes are ranked very differently by GWAS. 26
S24 GWAS and LoF plus damaging missense burden tests prioritize different genes for height. 27
S25 GWAS and LoF plus damaging missense burden tests prioritize different genes. . . . 28
S26 Top LoF burden genes are ranked very differently by GWAS even when upper bounding GWAS MAF. 29
S27 GWAS and LoF burden tests prioritize different genes for height even when upper bounding GWAS MAF. 30
S28 GWAS and LoF burden tests prioritize different genes even when upper bounding GWAS MAF. 31
S29 Top LoF burden genes are ranked very differently by GWAS when ranking by largest significant absolute effect size. 32
S30 GWAS and LoF burden tests prioritize different genes for height when ranking by largest significant absolute effect size. 33
S31 GWAS and LoF burden tests prioritize different genes when ranking by largest significant absolute effect size. 34
S32 p_{LoF} and s_{het} are negatively correlated. 35
S33 LoF and likely deleterious missense burden tests do not prioritize the most important genes. 36
S34 Burden tests prioritize specifically-expressed genes regardless of s_{het}. 37
S35 LoF plus damaging missense burden tests prioritize specifically-expressed genes. . . 38
S36 Expression specificity increases LoF burden test z-scores. 39
S37 Expression levels do not play a large role in GWAS coding heritability. 40
S38 Heritability enrichment increases with expression specificity; heritability enrichment weakly increases with overall expression levels. 41
S39 ATAC peak intensity increases heritability explained. 42
S40 Heritability enrichment increases with ATAC peak tissue specificity and intensity. . 43

S41 Specificity of ATAC peaks increases heritability explained across constraint bins. . . 44
S42 Relationship between constraint and heritability explained across specificity bins. . . 45
S43 Total proportion of heritability contributed by ATAC peaks of differing tissue specificities. . . . 46
S44 CDS length and expected number of unique LoFs are highly correlated. . . . 47
S45 Robustness of apparent pleiotropy to simulation parameter N_{eff}. . . . 48
S46 Robustness of apparent pleiotropy to simulation parameter t. . . . 49
S47 Robustness of apparent pleiotropy to simulation parameter p. . . . 50
S48 Robustness of apparent pleiotropy to simulation parameter f. . . . 51
S49 Probability of a variant being a GWAS hit is correlated with s_{het}. . . . 52
S50 Number of GWAS hits is predictive of γ^2. . . . 53

Description of Supplementary Tables **54**

Appendices **55**

A Sensitivity analyses for comparing the genes prioritized by GWAS and burden tests 55
B A mathematical model of association studies 56
C A model of context specificity to explain variant specificity 61
D LoF burden tests prioritize long genes 66
E Genetic drift makes the strongest GWAS hits appear more pleiotropic 67
F The impact of stabilizing selection on allele frequency in the context of trait specificity and genetic drift 68
G Connection between effect sizes and fitness under stabilizing selection 72
H LoF burden tests prioritize long genes 75
I Violations of model assumptions when estimating trait importance from association studies 76

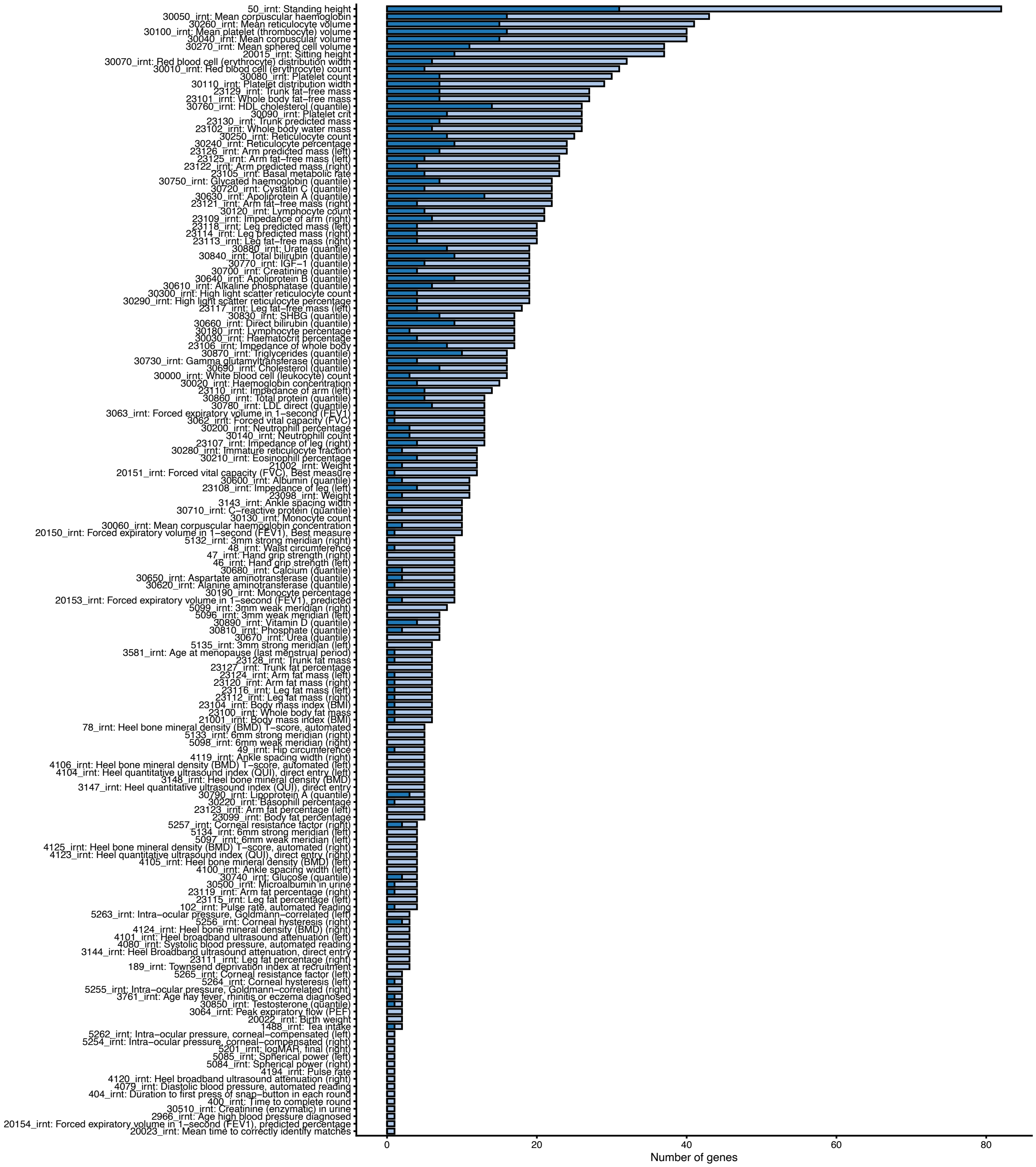

Supplementary Figure S1: **Limited GWAS overlap at top LoF burden hits.**
Bar charts of genome-wide significant LoF burden test genes. Dark blue bars correspond to genome-wide significant LoF burden test genes that also overlap a top GWAS locus (Methods). In particular, if there are K genome-wide significant burden genes for a trait, a gene is colored dark blue if it overlaps one of the top K most significant GWAS loci. Light blue bars are genome-wide significant LoF burden test genes that do not overlap a top GWAS locus.

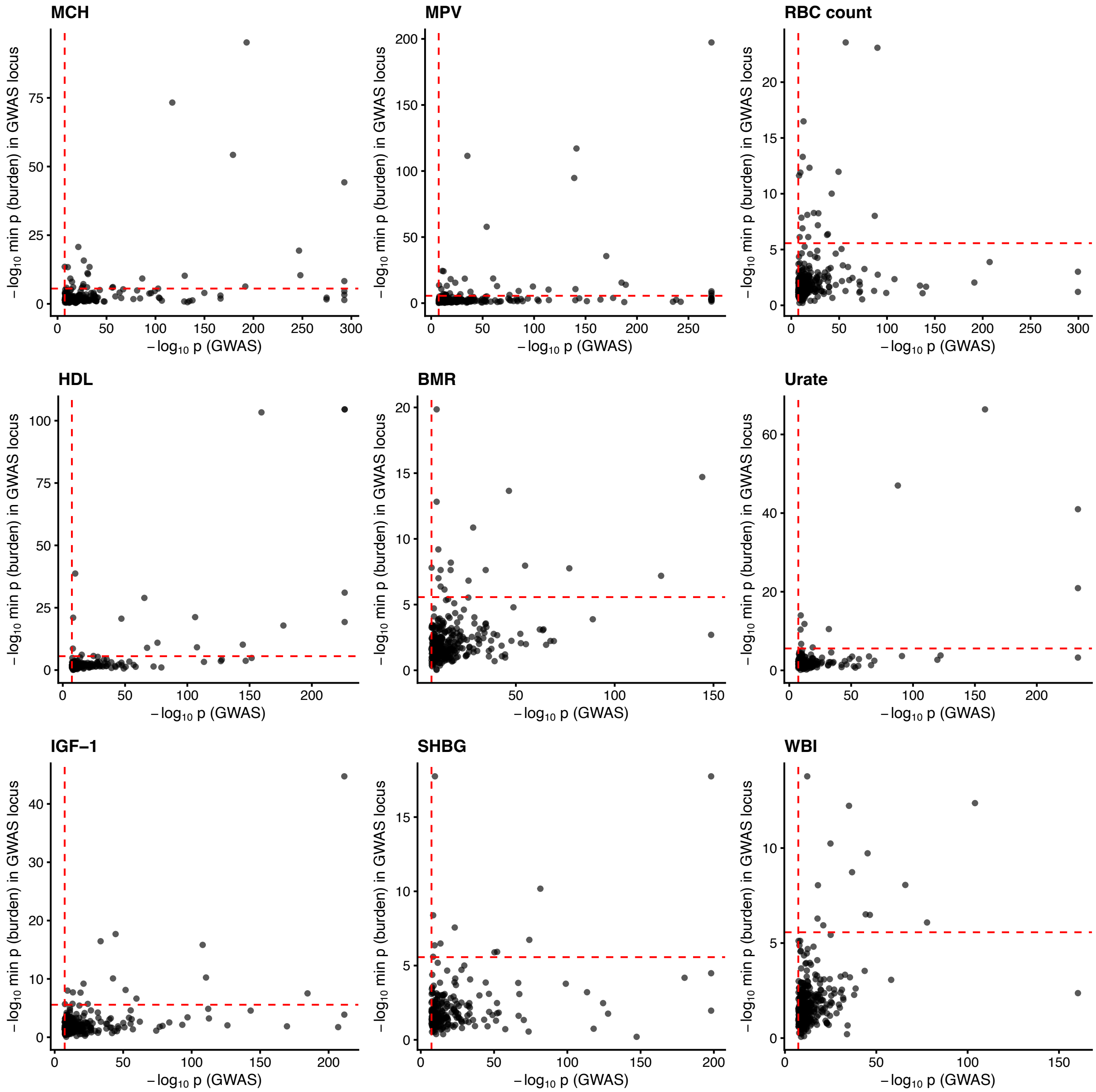

Supplementary Figure S2: **Strongest GWAS hits are not always strong LoF burden hits.**
*Extended version of Figure 1**D**, including 9 additional traits. Each point is a significant GWAS locus (Methods). Dashed red lines are thresholds for genome-wide significance.*

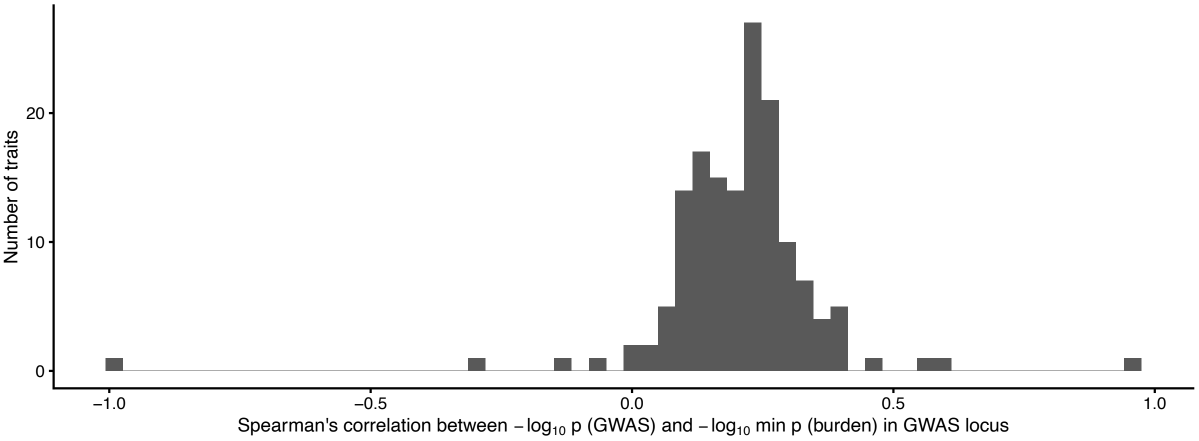

Supplementary Figure S3: **Modest correlation between GWAS and LoF burden test p-value ranks.**

Histogram of Spearman's ρ between the minimum GWAS $-\log_{10}$ *p-value of any variant within a given GWAS locus and the minimum LoF burden* $-\log_{10}$ *p-value of any gene overlapping that locus. By definition all GWAS loci contain at least one genome-wide significant variant (Methods).*

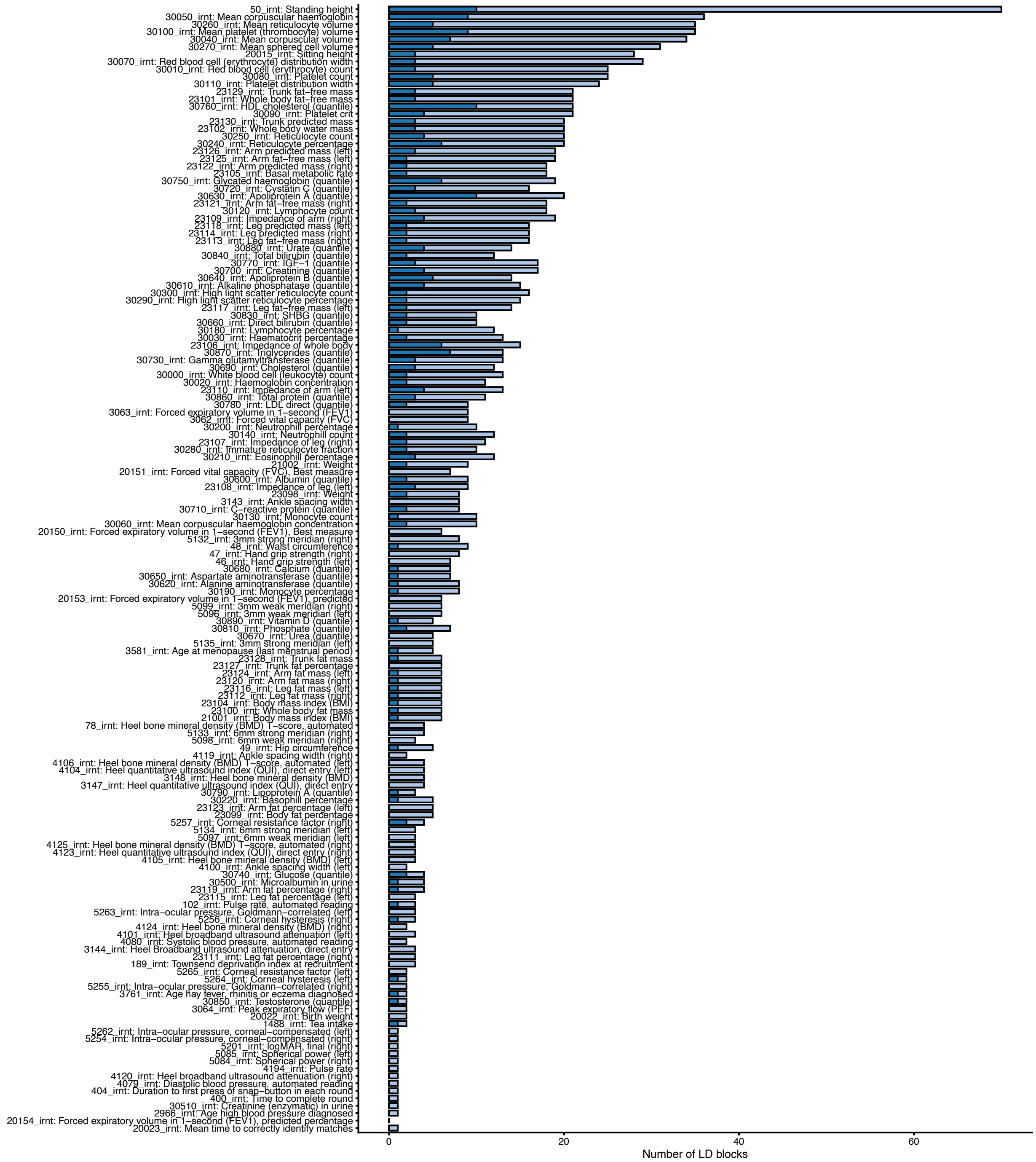

Supplementary Figure S4: **Limited GWAS overlap at top LoF burden LD blocks.**

Alternate version of Supplementary Figure S1 but using LD blocks instead of GWAS loci. Dark blue bars correspond to LD blocks that contain a genome-wide significant LoF burden test gene that are also top LD blocks for GWAS. Light blue bars are LD blocks containing genome-wide significant LoF burden test genes that are not also top GWAS LD blocks.

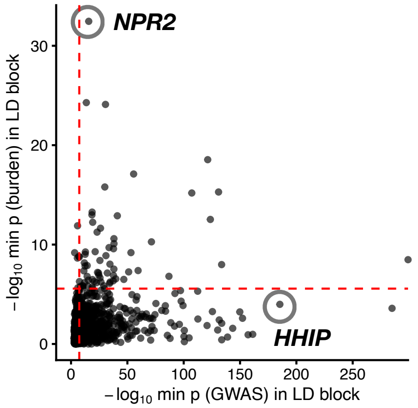

Supplementary Figure S5: **GWAS and LoF burden tests prioritize different LD blocks for height.** *Alternate version of Figure 1***D** *but using LD blocks instead of GWAS loci. Each point is an LD block. Dashed red lines are thresholds for genome-wide significance.*

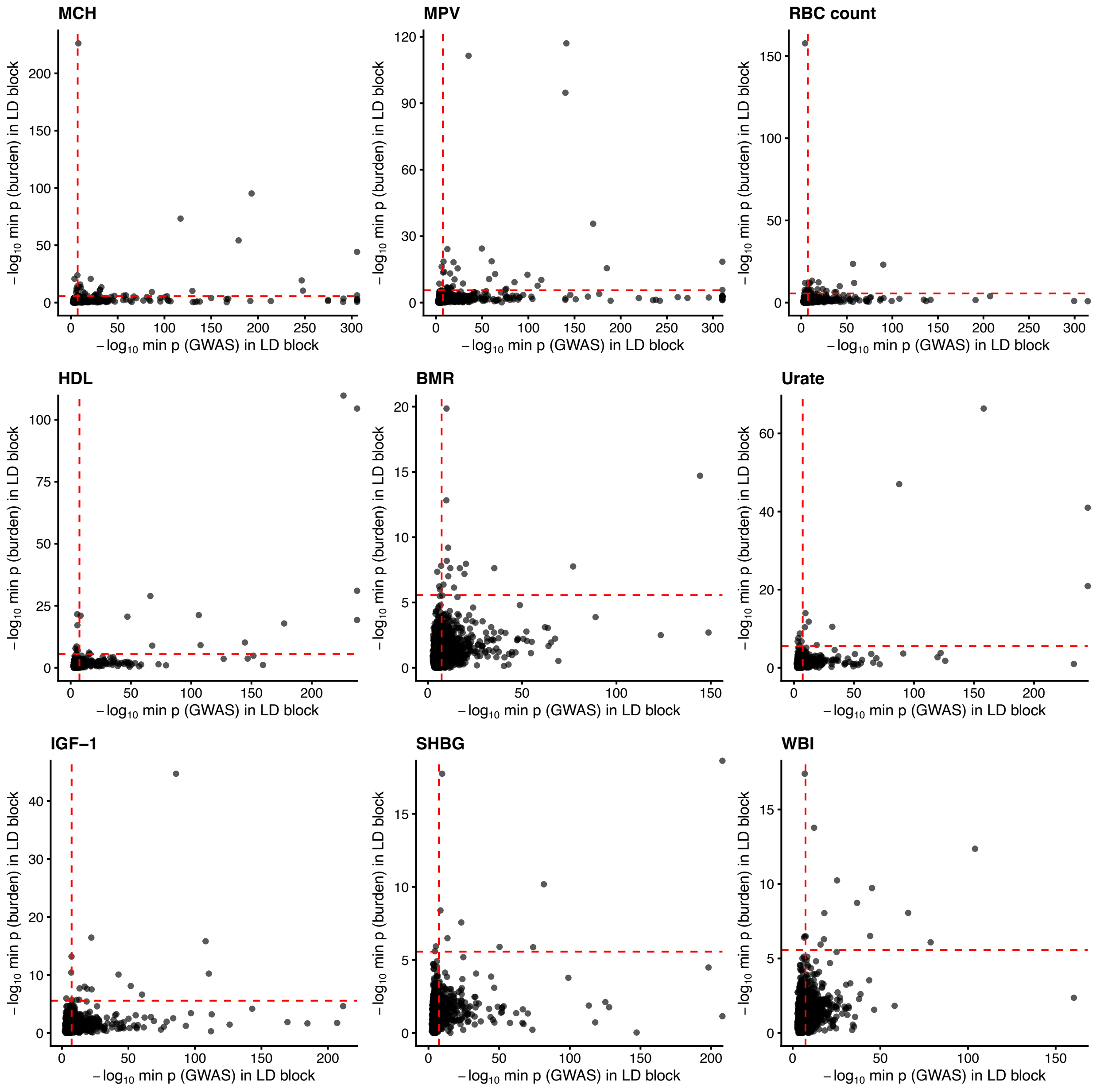

Supplementary Figure S6: **GWAS and LoF burden tests prioritize different LD blocks across traits.**

Alternate version of Supplementary Figure S2 but using LD blocks instead of GWAS loci. Each point is an LD block. Dashed red lines are thresholds for genome-wide significance.

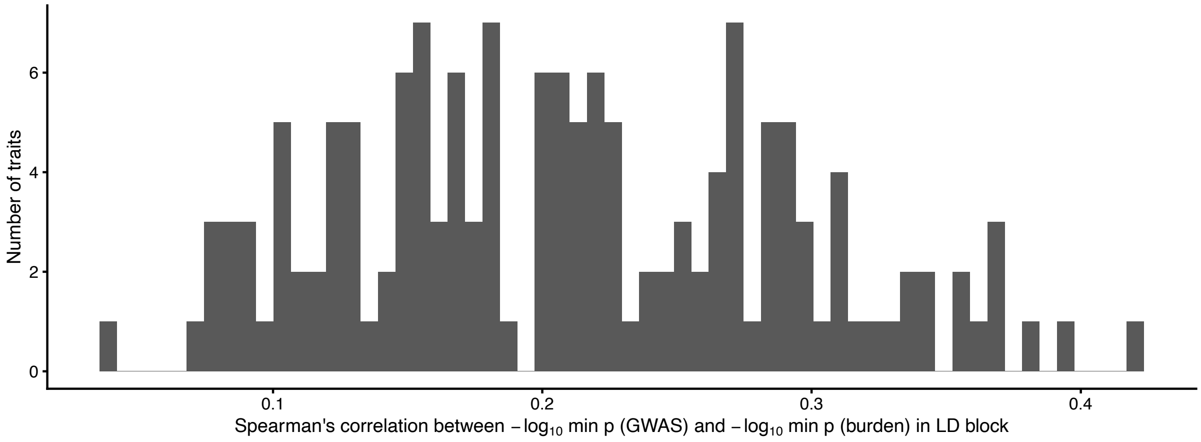

Supplementary Figure S7: **Modest correlation between GWAS and LoF burden test p-value ranks across LD blocks.**

Alternate version of Supplementary Figure S3 but using LD blocks instead of GWAS loci. Histogram of Spearman's ρ *between the minimum GWAS* $-\log_{10}$ *p-value and the minimum LoF burden* $-\log_{10}$ *p-value across LD blocks.*

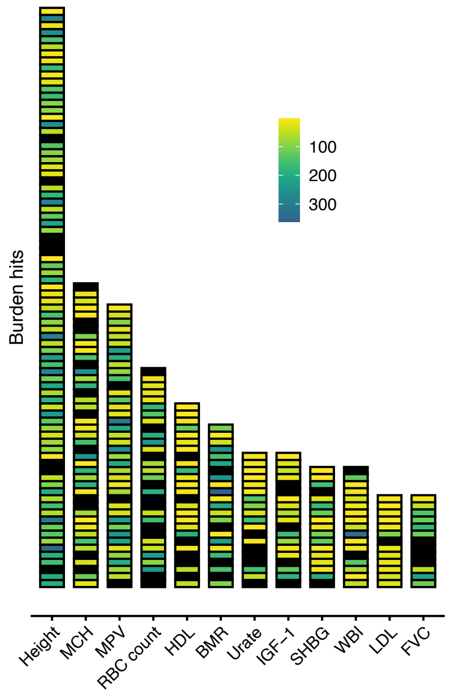

Supplementary Figure S8: **Top burden genes are ranked very differently by GWAS when using COJO SNPs.**

*Alternate version of Figure 1***C** *but ranking genes in GWAS by using the p-values of conditionally independent GWAS hits as obtained using COJO. Vertical order indicates rank in burden tests, color indicates rank in GWAS.*

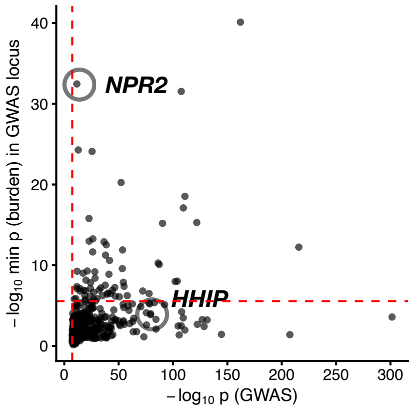

Supplementary Figure S9: **GWAS and LoF burden tests prioritize different GWAS loci for height when using COJO SNPs.**

*Alternate version of Figure 1**D** but ranking loci in GWAS by using the p-values of conditionally independent GWAS hits as obtained using COJO.*

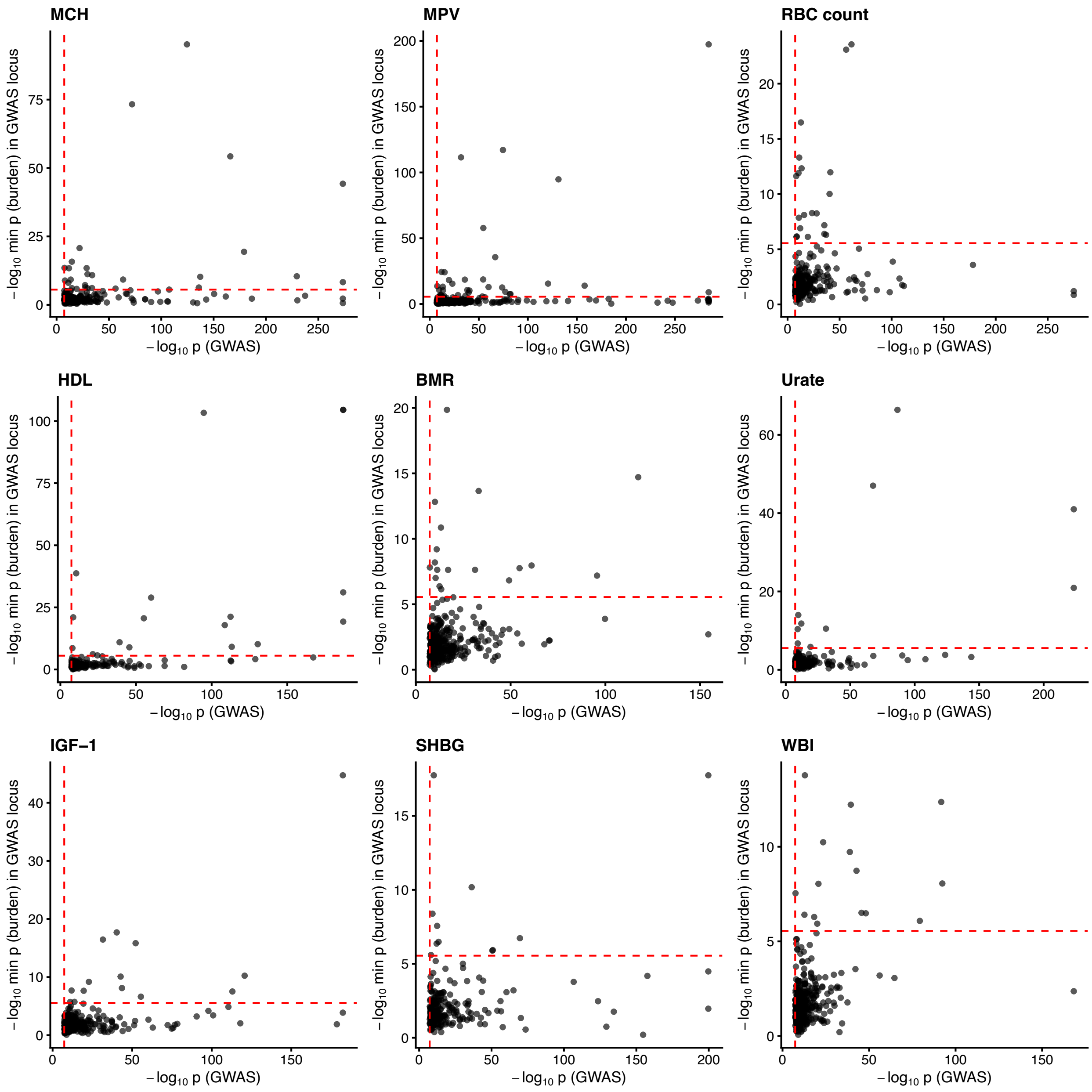

Supplementary Figure S10: **GWAS and LoF burden tests prioritize different GWAS loci across traits when using COJO SNPs.**

Alternate version of Supplementary Figure S2 but ranking loci in GWAS by using the p-values of conditionally independent GWAS hits as obtained using COJO.

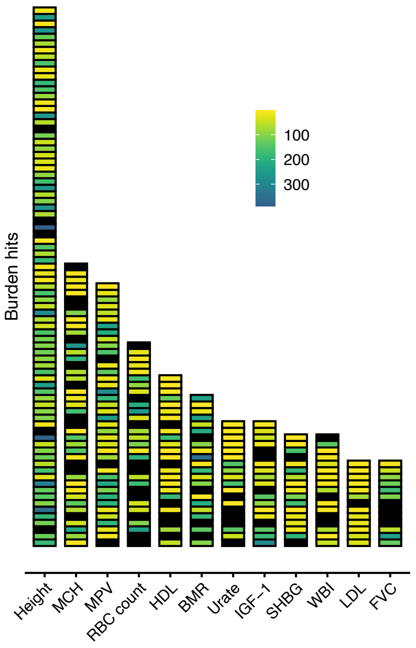

Supplementary Figure S11: **Top burden genes are ranked very differently by GWAS when using more aggressively LD-clumped SNPs.**

*Alternate version of Figure 1**C** but ranking genes in GWAS by using the p-values of more aggressively LD-clumped GWAS hits. Vertical order indicates rank in burden tests, color indicates rank in GWAS.*

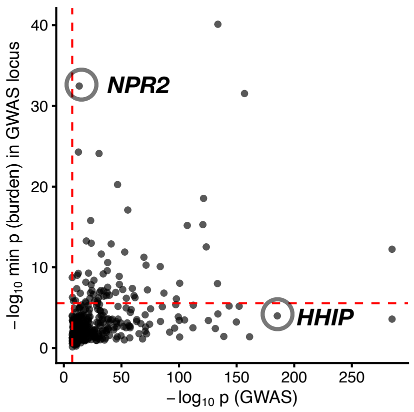

Supplementary Figure S12: **GWAS and LoF burden tests prioritize different GWAS loci for height when using more aggressively LD-clumped SNPs.**

*Alternate version of Figure 1**D** but ranking loci in GWAS by using the p-values of more aggressively LD-clumped GWAS hits.*

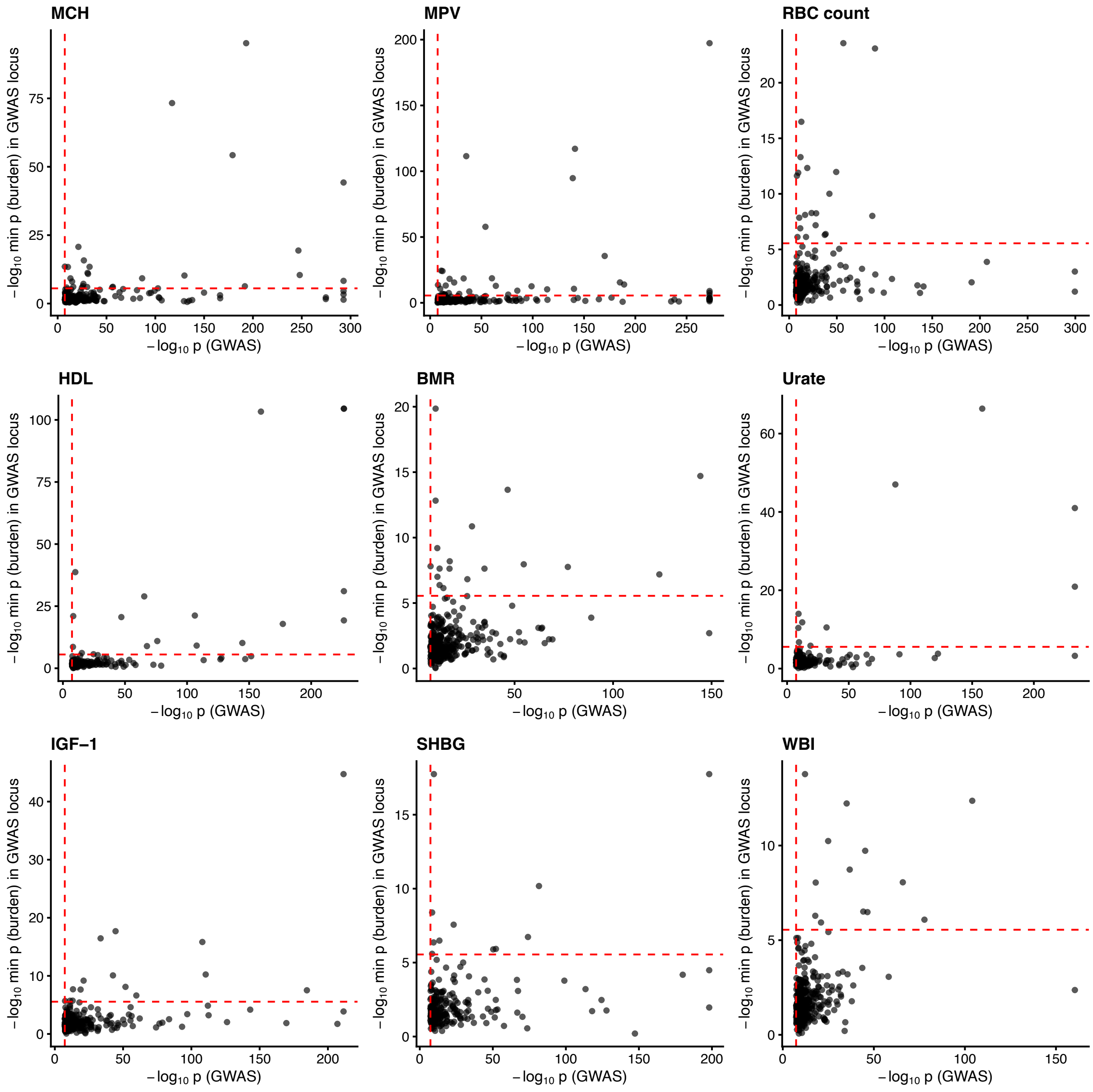

Supplementary Figure S13: **GWAS and LoF burden tests prioritize different GWAS loci across traits when using more aggressively LD-clumped SNPs.**

Alternate version of Supplementary Figure S2 but ranking loci in GWAS by using the p-values of more aggressively LD-clumped SNPs.

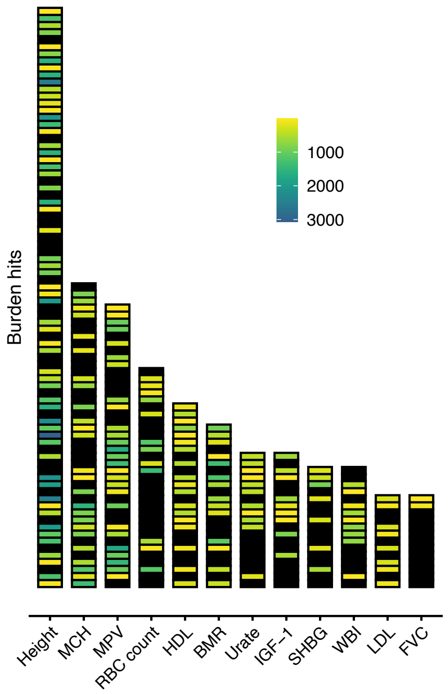

Supplementary Figure S14: **Top burden genes are ranked very differently by GWAS when ranking using MAGMA.**

*Alternate version of Figure 1***C** *but ranking genes in GWAS with MAGMA. Vertical order indicates rank in burden tests, color indicates rank in GWAS.*

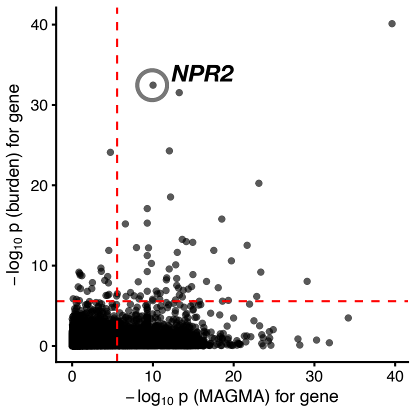

Supplementary Figure S15: **GWAS and LoF burden tests prioritize different genes for height when ranking genes in GWAS using MAGMA.**
*Alternate version of Figure 1**D** but ranking genes in GWAS with MAGMA.*

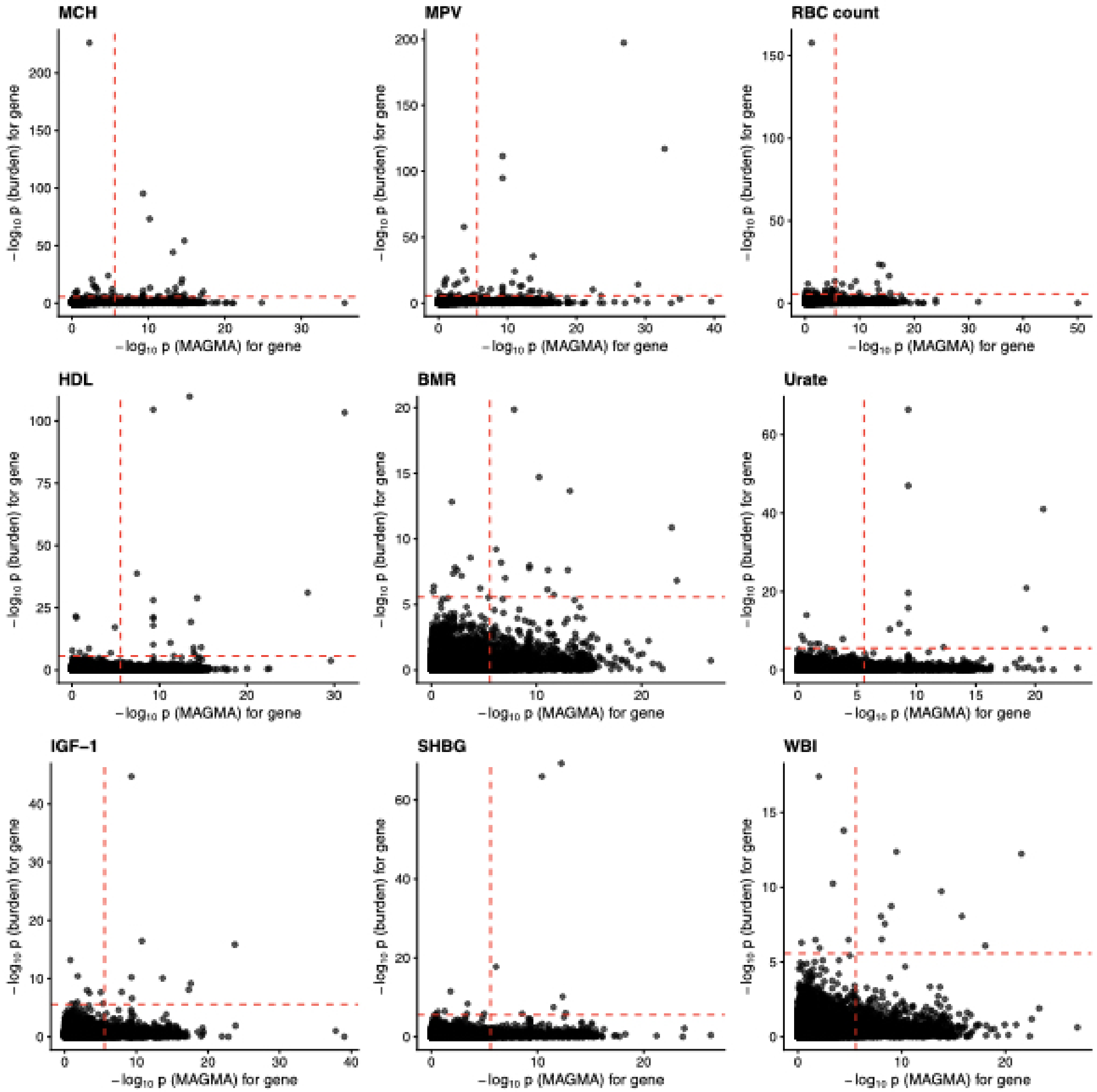

Supplementary Figure S16: **GWAS and LoF burden tests prioritize different genes when ranking genes in GWAS using MAGMA.**

Alternate version of Supplementary Figure S2 but ranking genes in GWAS with MAGMA.

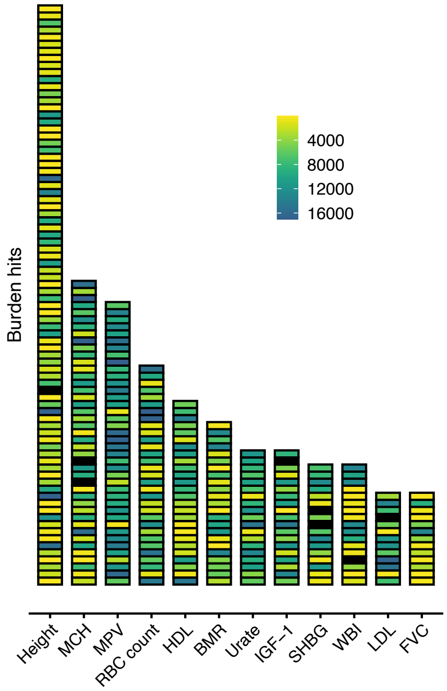

Supplementary Figure S17: **Top burden genes are ranked very differently by GWAS when ranking using PoPS.**

*Alternate version of Figure 1**C** but ranking genes in GWAS with PoPS. Vertical order indicates rank in burden tests, color indicates rank in GWAS. Every gene gets scored by PoPS so the color scale covers more genes than in other analyses.*

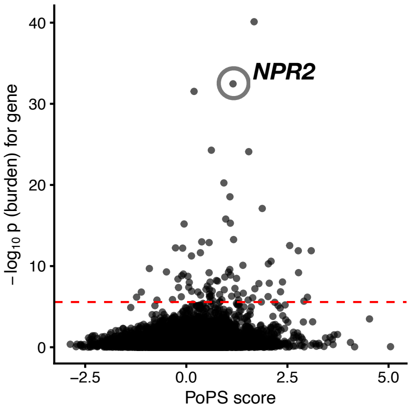

Supplementary Figure S18: **GWAS and LoF burden tests prioritize different genes for height when ranking genes in GWAS using PoPS.**
*Alternate version of Figure 1***D** *but ranking genes in GWAS with PoPS.*

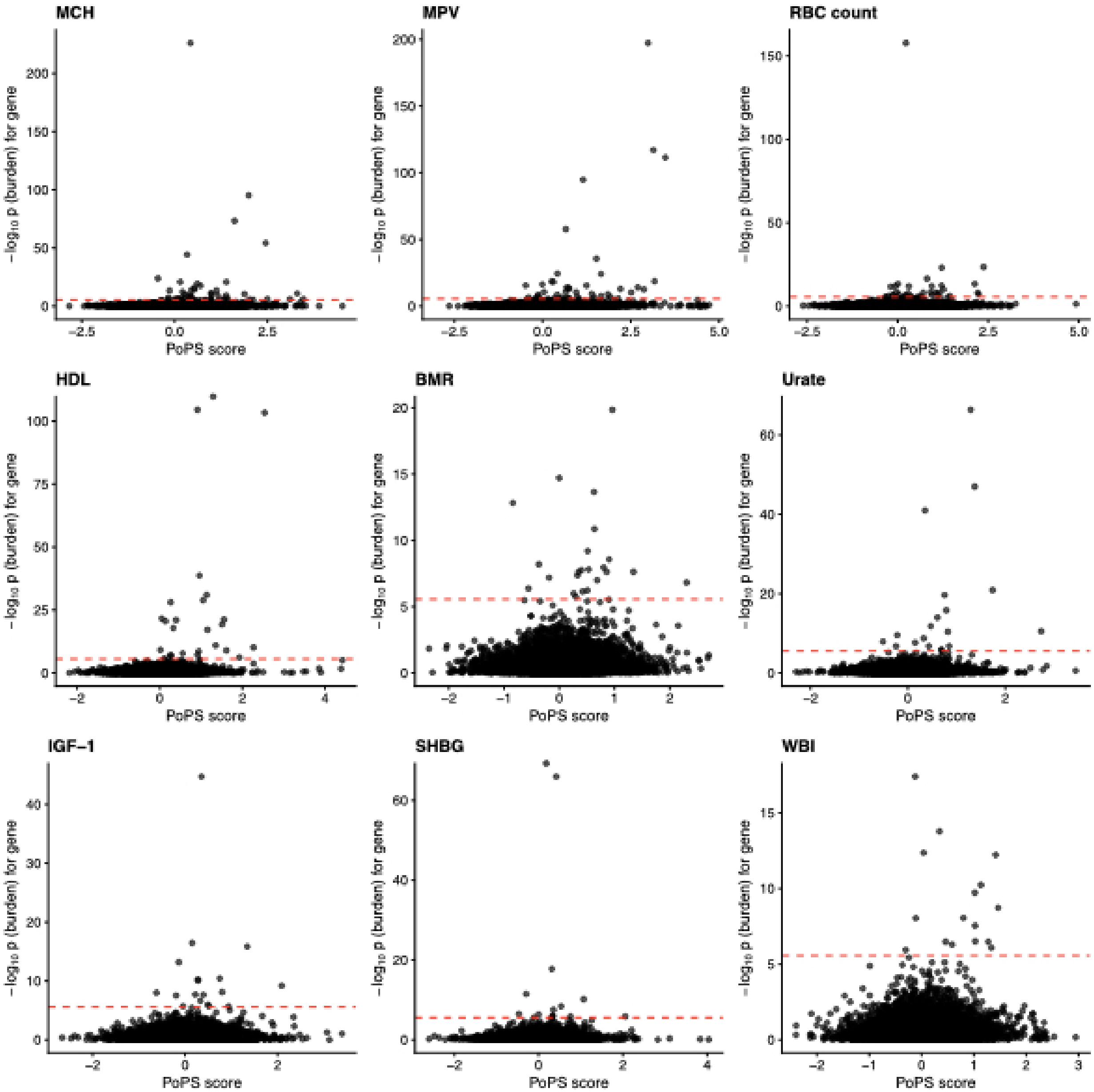

Supplementary Figure S19: **GWAS and LoF burden tests prioritize different genes when ranking genes in GWAS using PoPS.**
Alternate version of Supplementary Figure S2 but ranking genes in GWAS with PoPS.

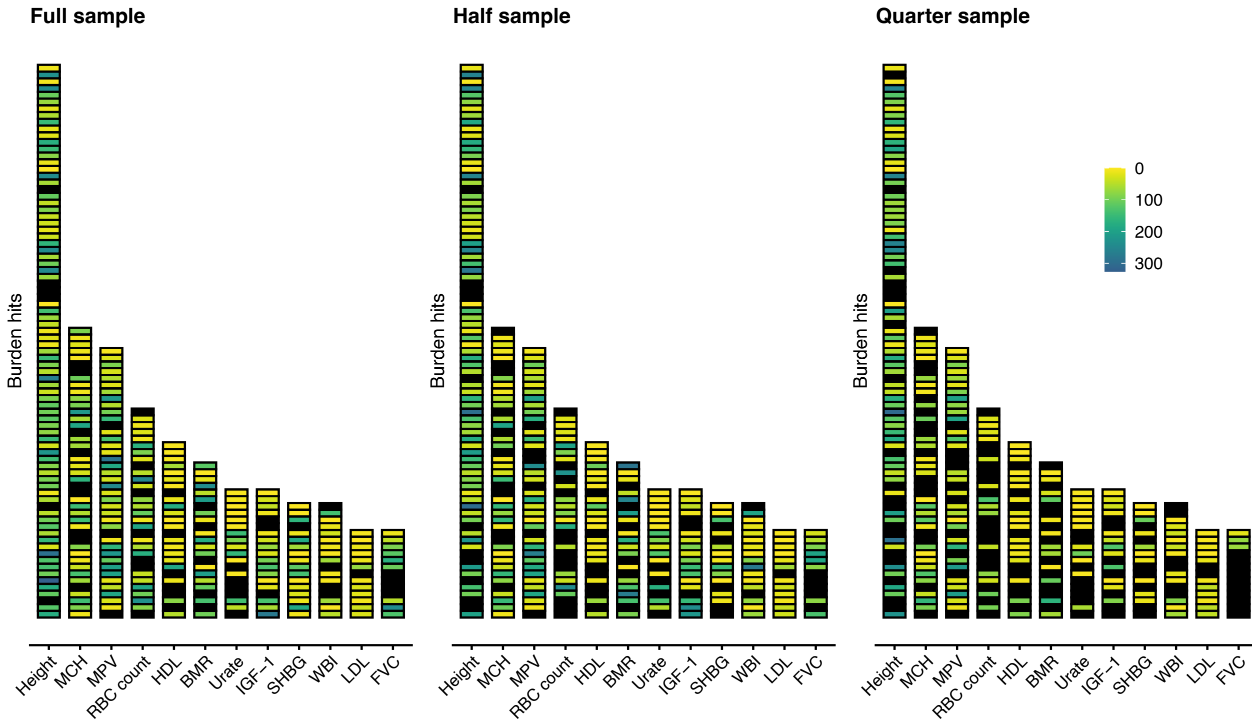

Supplementary Figure S20: **Top burden genes are ranked very differently by GWAS regardless of GWAS sample size.**

Alternate version of Figure **1C** *but simulating GWAS with either half or one quarter as many individuals (see Methods). Vertical order indicates rank in burden tests, color indicates rank in GWAS.*

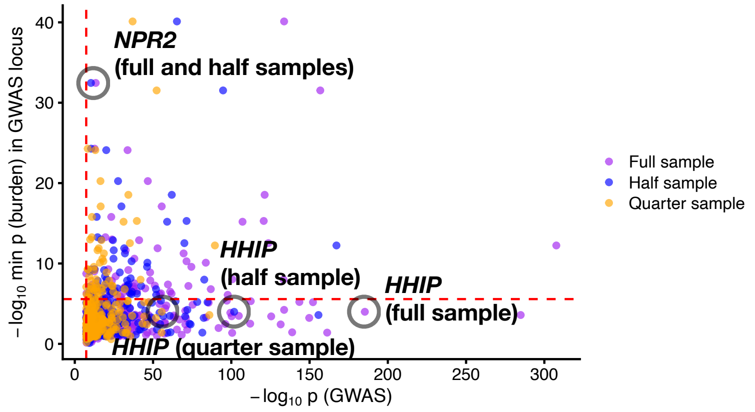

Supplementary Figure S21: **GWAS and LoF burden tests prioritize different genes for height regardless of GWAS sample size.**

*Alternate version of Figure 1*__D__ *but simulating GWAS with either half or one quarter as many individuals (see Methods). Note that NPR2 is not included in the plot for the quarter-sized GWAS as it was not contained in any genome-wide significant GWAS locus.*

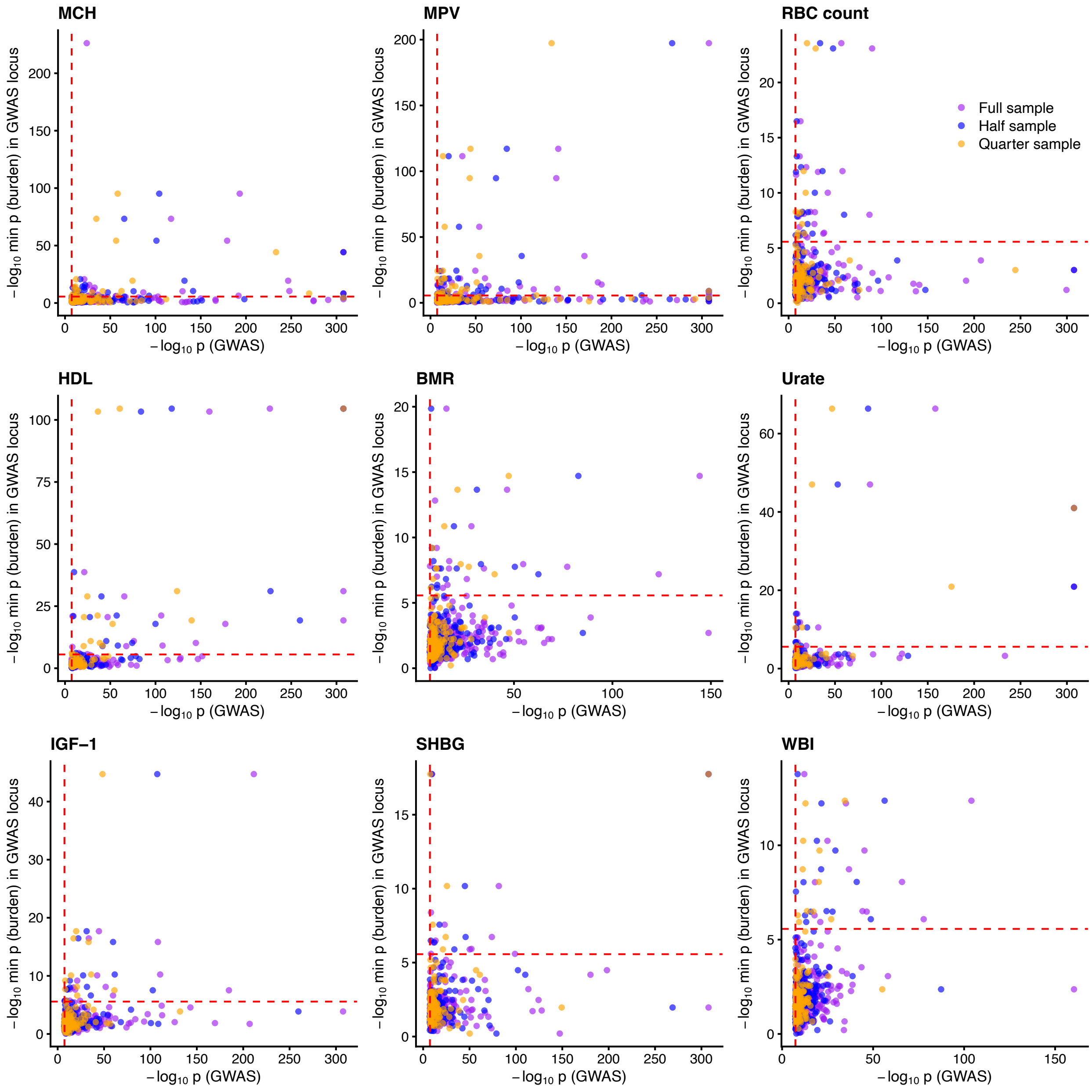

Supplementary Figure S22: **GWAS and LoF burden tests prioritize different genes regardless of GWAS sample size.**

Alternate version of Supplementary Figure S2 but simulating GWAS with either half or one quarter as many individuals (see Methods).

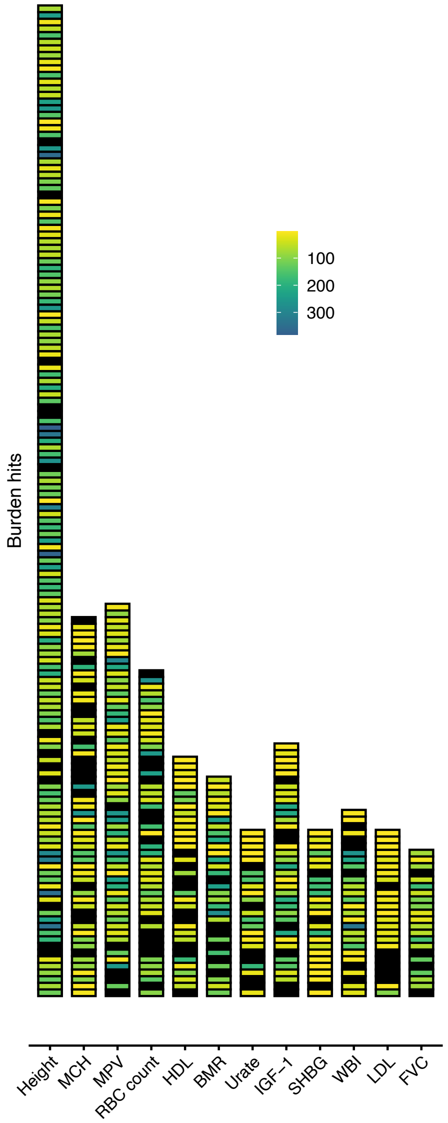

Supplementary Figure S23: **Top LoF plus damaging missense burden genes are ranked very differently by GWAS.**

*Alternate version of Figure 1**C** but using burden tests that use both LoFs and damaging missense variants. Vertical order indicates rank in burden tests, color indicates rank in GWAS.*

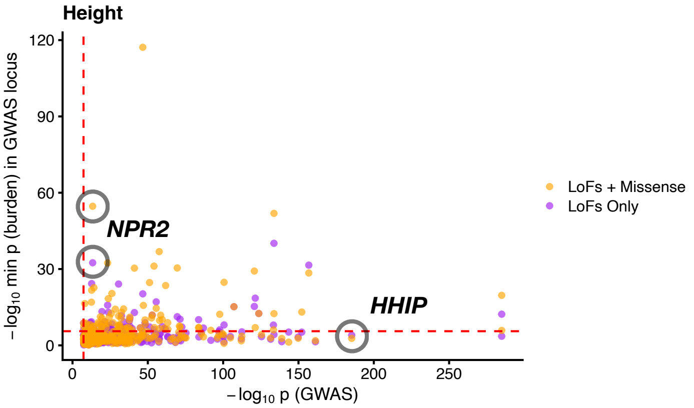

Supplementary Figure S24: **GWAS and LoF plus damaging missense burden tests prioritize different genes for height.**

*Alternate version of Figure 1**D** but using burden tests that use both LoFs and damaging missense variants.*

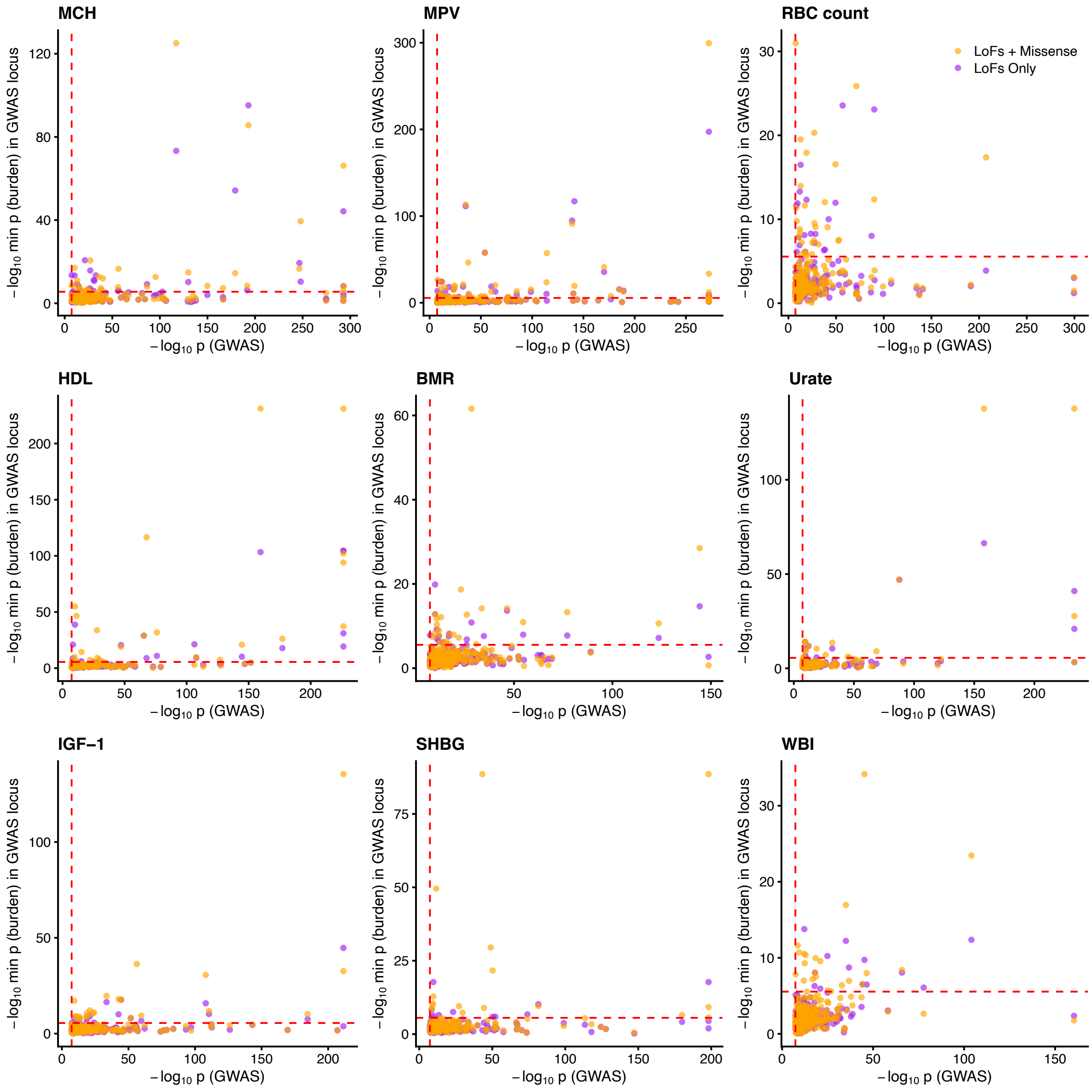

Supplementary Figure S25: **GWAS and LoF plus damaging missense burden tests prioritize different genes.**
Alternate version of Supplementary Figure S2 but using burden tests that use both LoFs and damaging missense variants.

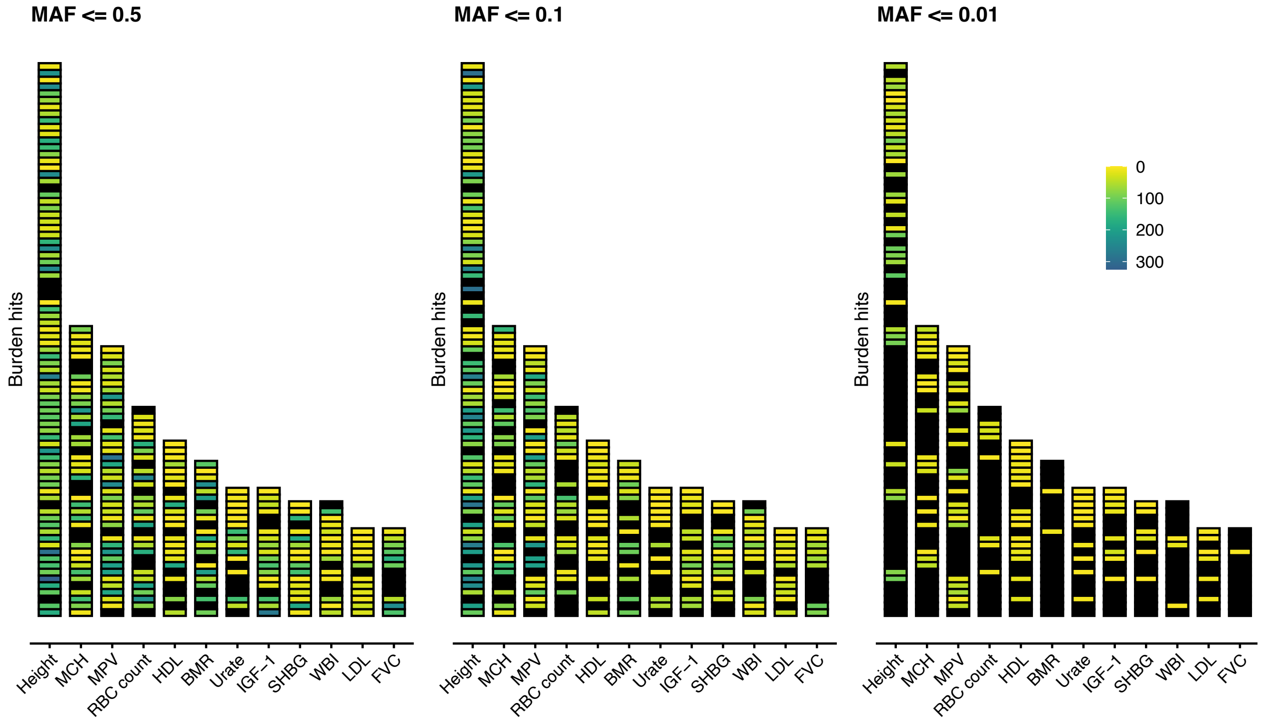

Supplementary Figure S26: **Top LoF burden genes are ranked very differently by GWAS even when upper bounding GWAS MAF.**

*Alternate version of Figure 1***C** *but using only GWAS variants below a given MAF threshold. Vertical order indicates rank in burden tests, color indicates rank in GWAS.*

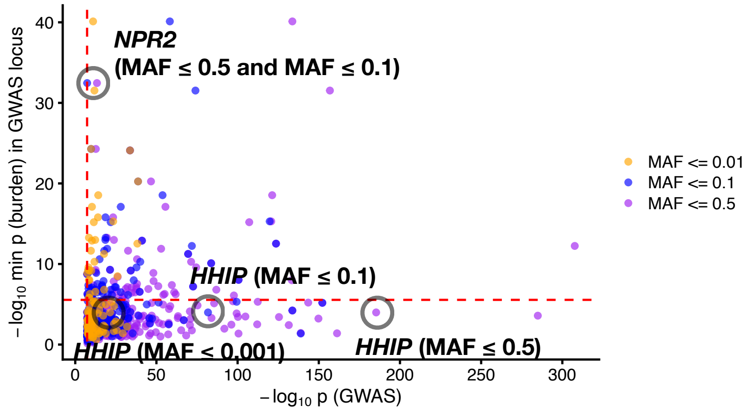

Supplementary Figure S27: **GWAS and LoF burden tests prioritize different genes for height even when upper bounding GWAS MAF.**

*Alternate version of Figure 1***D** *but using only GWAS variants below a given MAF threshold.*

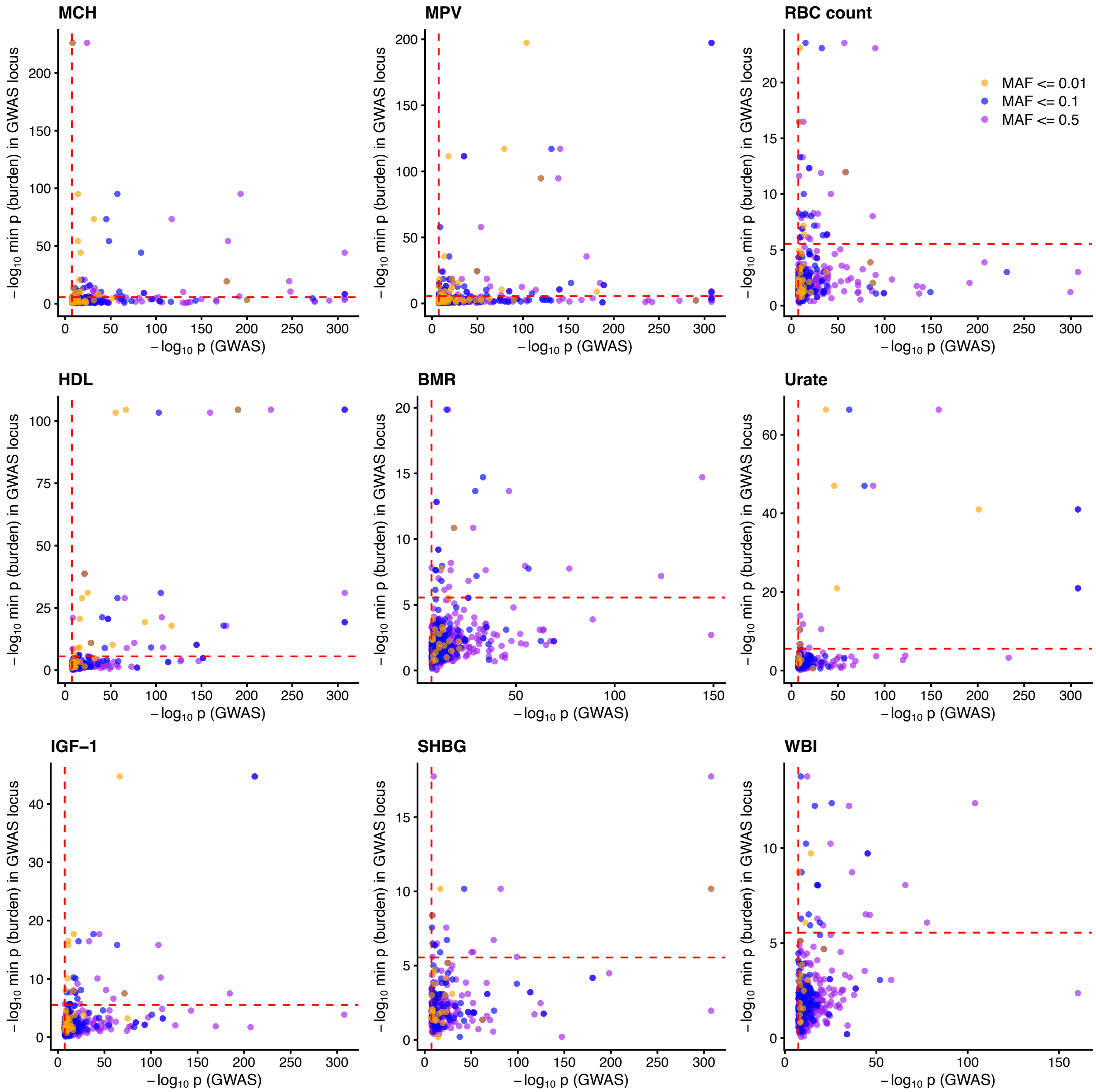

Supplementary Figure S28: **GWAS and LoF burden tests prioritize different genes even when upper bounding GWAS MAF.**

Alternate version of Supplementary Figure S2 but using only GWAS variants below a given MAF threshold.

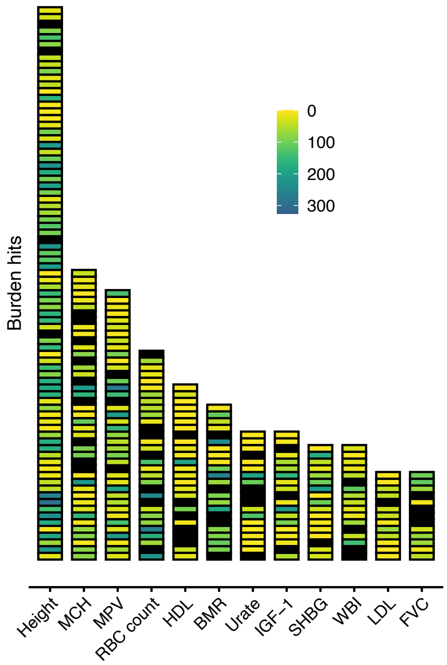

Supplementary Figure S29: **Top LoF burden genes are ranked very differently by GWAS when ranking by largest significant absolute effect size.**

*Alternate version of Figure 1**C** but ranking by largest significant absolute effect size in both GWAS and the burden test. Vertical order indicates rank in burden tests, color indicates rank in GWAS.*

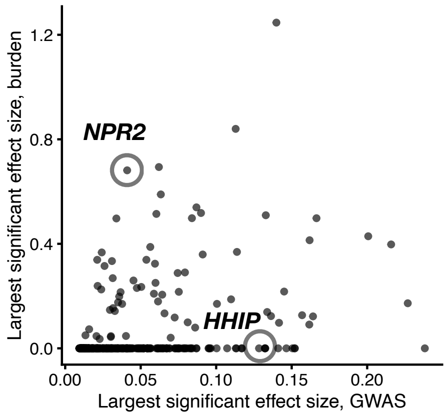

Supplementary Figure S30: **GWAS and LoF burden tests prioritize different genes for height when ranking by largest significant absolute effect size.**

*Alternate version of Figure 1**D** but ranking by largest significant absolute effect size in both GWAS and the burden test.*

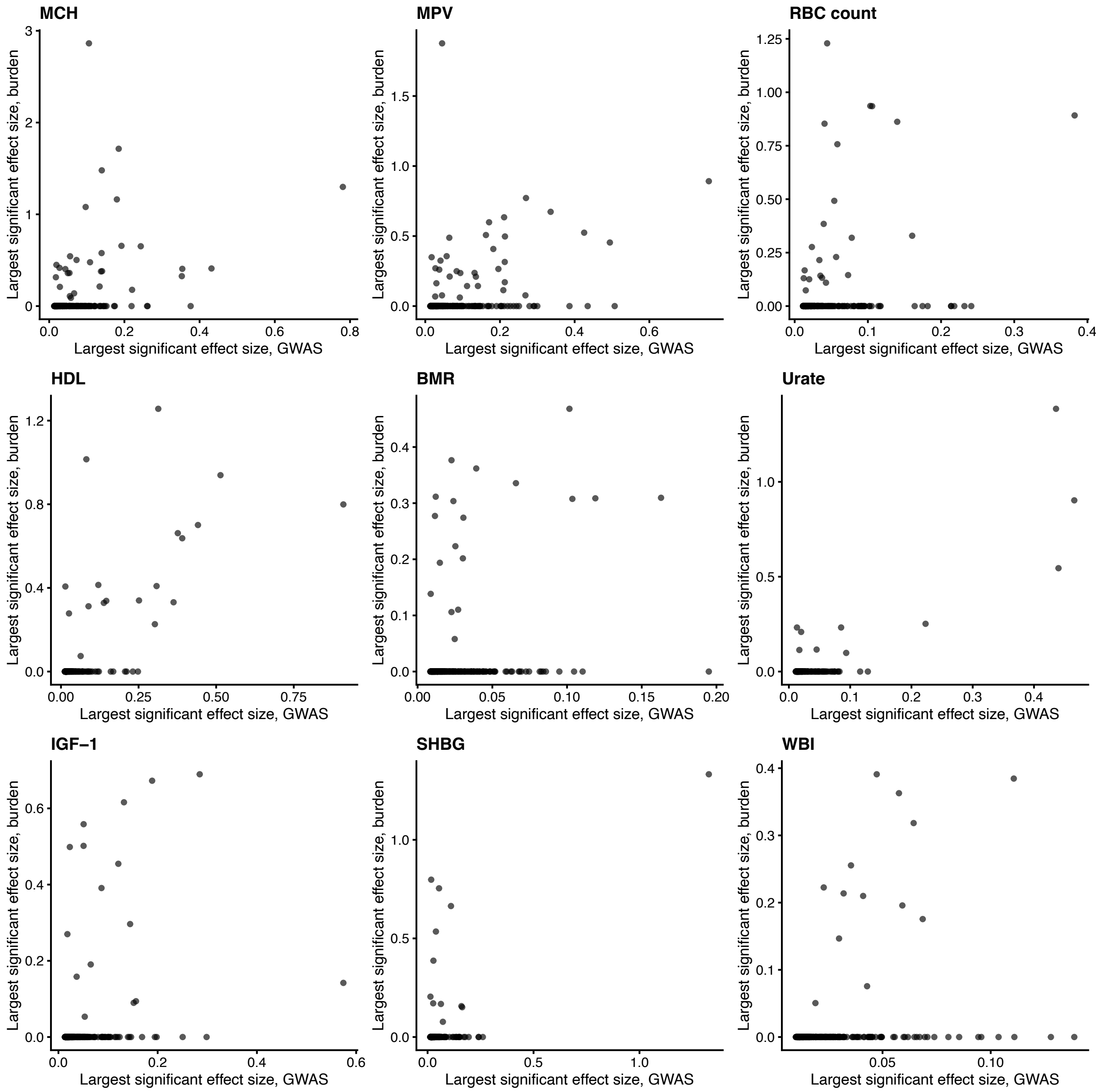

Supplementary Figure S31: **GWAS and LoF burden tests prioritize different genes when ranking by largest significant absolute effect size.**
Alternate version of Supplementary Figure S2 but ranking by largest significant absolute effect size in both GWAS and the burden test.

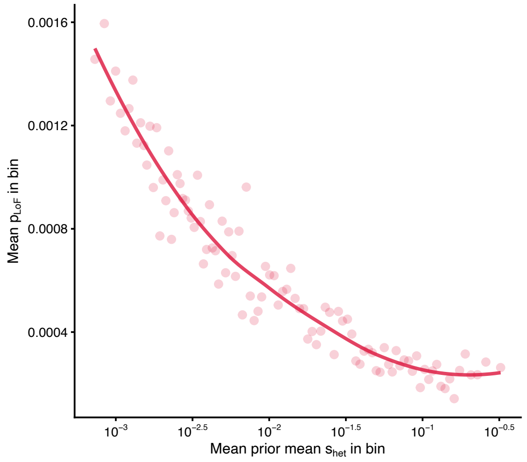

Supplementary Figure S32: p_{LoF} **and** $s_{\mathbf{het}}$ **are negatively correlated.**
*Alternate version of Figure 3**B** but binning genes by the prior mean s_{het} as reported by [84]. Spearman's ρ between prior mean s_{het} and $p_{LoF} = -0.502$; p-value $< 10^{-15}$; $N = 18{,}154$ genes. These estimates of s_{het} are learned using GeneBayes [84], which uses frequency data across genes to learn a function mapping gene features (e.g., expression patterns across tissues) to a prior on s_{het}. In the main text, we used GeneBayes posterior mean estimates, which use this learned prior for each gene along with that gene's p_{LoF} to estimate s_{het}. Here we use the prior mean, which uses the p_{LoF} data across genes to learn per-gene priors, but does not use a gene's p_{LoF} when estimating its s_{het}.*

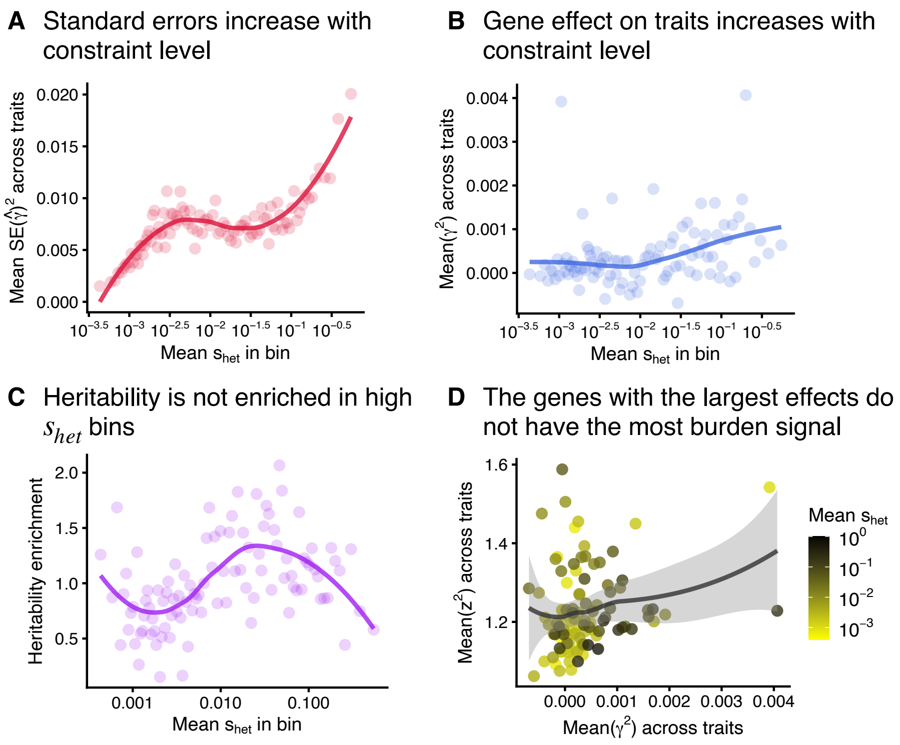

Supplementary Figure S33: **LoF and likely deleterious missense burden tests do not prioritize the most important genes.**

A) *Genes were binned by an estimate of* s_{het} *[84] with approximately 184 genes per bin. Squared standard errors for* $\widehat{\gamma}$ *were then averaged across across 27 genetically uncorrelated traits (Methods) and across genes within each bin. The trend line was fit using LOESS.* **B**) *Similar to* **A**, *but averaging over an unbiased estimate of the mean of* γ_t^2 *across traits.* **C**) *Similar to* **A** *but with a normalized inverse variance-weighted average of heritability enrichments across traits.* **D**) *Genes were binned as in* **A**, *and the mean of squared z-scores,* z^2, *across traits was plotted against the average of an unbiased estimate of the mean of* γ_t^2 *across traits. Points are colored by the mean* s_{het} *within the bin and the trend line was fit using LOESS.*

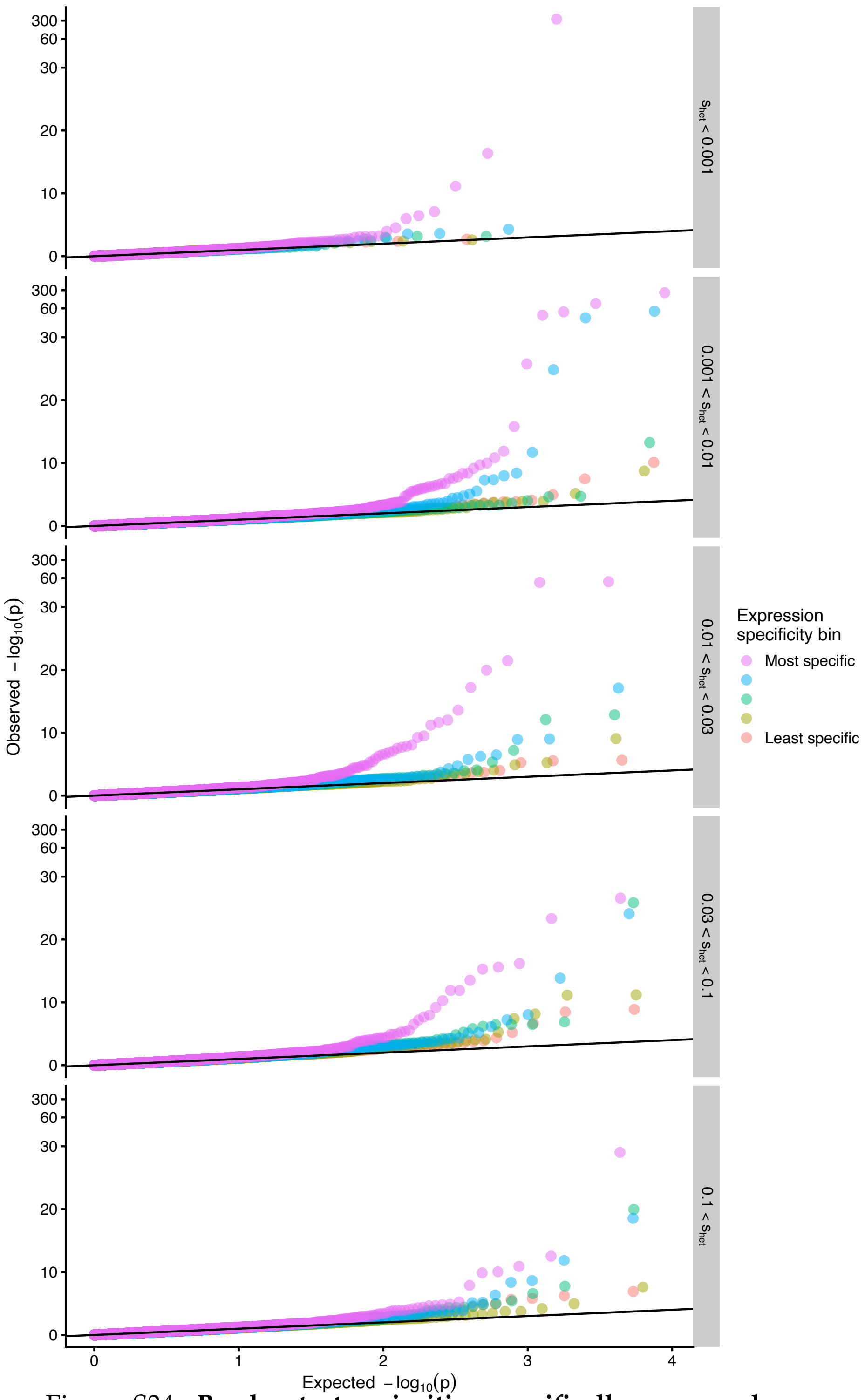

Supplementary Figure S34: **Burden tests prioritize specifically-expressed genes regardless of s_{het}.**

Quantile-quantile plots of LoF burden test p-values across 9 trait-tissue pairs, faceted by s_{het} value of the gene. Genes were stratified for each trait-tissue pair based on the specificity of their expression to the trait-relevant tissue. The y-axes have all been non-linearly transformed in the same way.

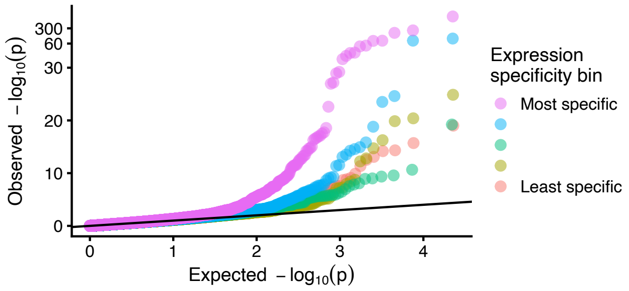

Supplementary Figure S35: **LoF plus damaging missense burden tests prioritize specifically-expressed genes.**

Quantile-quantile plot of LoF plus likely damaging missense burden test p-values across 9 trait-tissue pairs. Genes were stratified for each trait-tissue pair based on the specificity of their expression to the trait-relevant tissue. The y-axis has been non-linearly transformed.

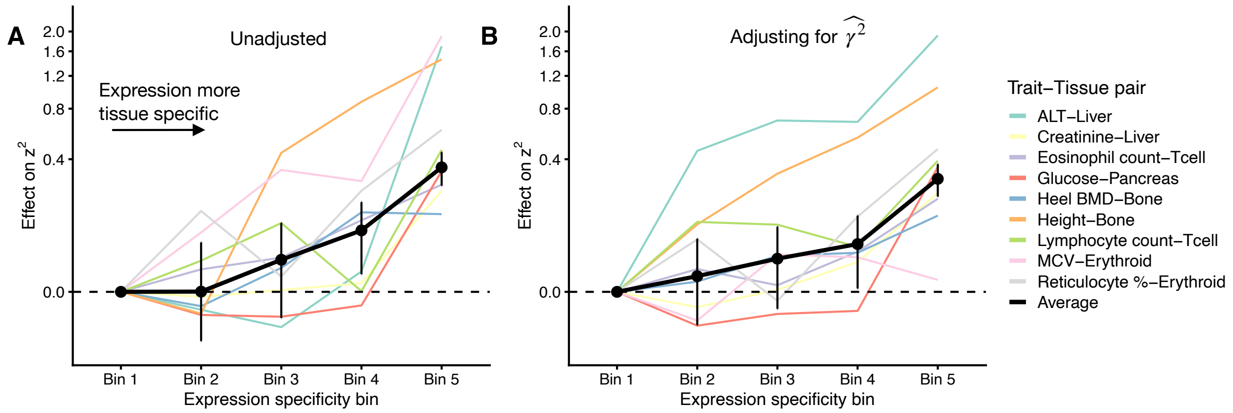

Supplementary Figure S36: **Expression specificity increases LoF burden test z-scores.** *For 9 trait-tissue pairs we regressed LoF burden test z^2 for each gene on either* **A**) *expression specificity bin or* **B**) *expression specificity bin and an unbiased estimate of γ^2, $\widehat{\gamma^2}$. Since the 5 bins are co-linear, we report all regression coefficients relative to the effect in expression specificity bin 1. Colored lines are regression coefficients for individual trait-tissue pairs. The black line is the inverse variance-weighted average across trait-tissue pairs. The y-axes have been non-linearly transformed.*

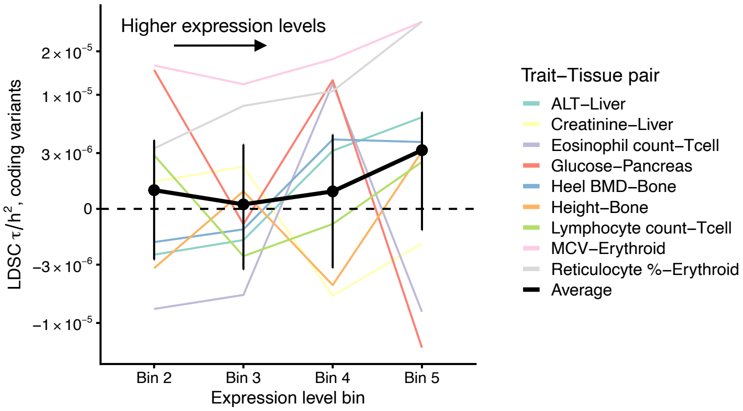

Supplementary Figure S37: **Expression levels do not play a large role in GWAS coding heritability.**

S-LDSC analysis results for 9 trait-tissue pairs. Results are reported in terms of τ/h^2, a measure of an annotation's effect on proportion of heritability explained. Variants are binned by the expression level (as measured by TPM) of the corresponding gene. Since the 5 bins are co-linear, we drop the bin 1 annotation and only report results for the remaining bins. These results are from a joint analysis including both expression specificity and expression level bins as covariates. Colored lines are τ estimates for individual trait-tissue pairs. The black line is the inverse variance-weighted average across trait-tissue pairs. The y-axis has been non-linearly transformed.

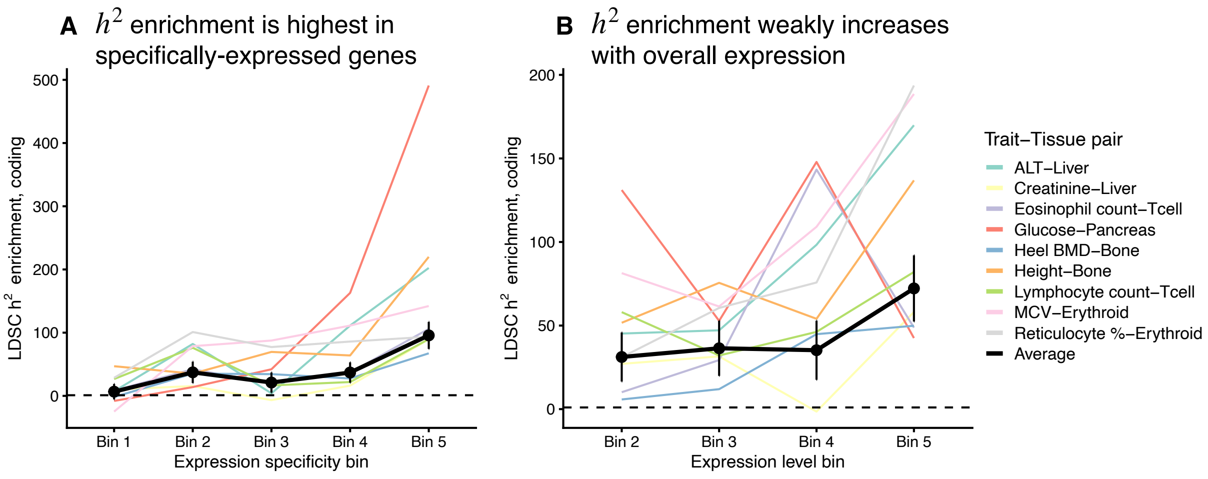

Supplementary Figure S38: **Heritability enrichment increases with expression specificity; heritability enrichment weakly increases with overall expression levels.**
S-LDSC analysis results for 9 trait-tissue pairs. Results are reported in terms of h^2 enrichment. Variants are binned by the expression level (as measured by TPM) of the corresponding gene as well as its tissue specificity (Methods). Since the 5 TPM bins are co-linear, we drop the bin 1 annotation and only report results for the remaining bins. These results are from a joint analysis including both expression specificity and expression level bins as covariates. Colored lines are h^2 enrichment estimates for individual trait-tissue pairs. The black line is the inverse variance-weighted average across trait-tissue pairs. Note the different y-axes on the two figures.

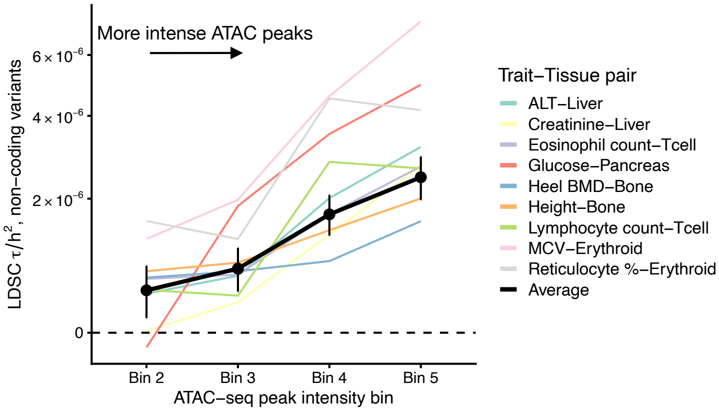

Supplementary Figure S39: **ATAC peak intensity increases heritability explained.**
S-LDSC analysis results for 9 trait-tissue pairs. Results are reported in terms of τ/h^2*, a measure of an annotation's effect on proportion of heritability explained. Variants are binned by the intensity of their ATAC-seq peaks (Methods). Since the 5 bins are co-linear, we drop the bin 1 annotation and only report results for the remaining bins. These results are from a joint analysis including both ATAC specificity and ATAC intensity bins as covariates. Colored lines are the* τ *estimates for individual trait-tissue pairs. The black line is the inverse variance-weighted average across trait-tissue pairs. Note that the y-axis has been non-linearly transformed.*

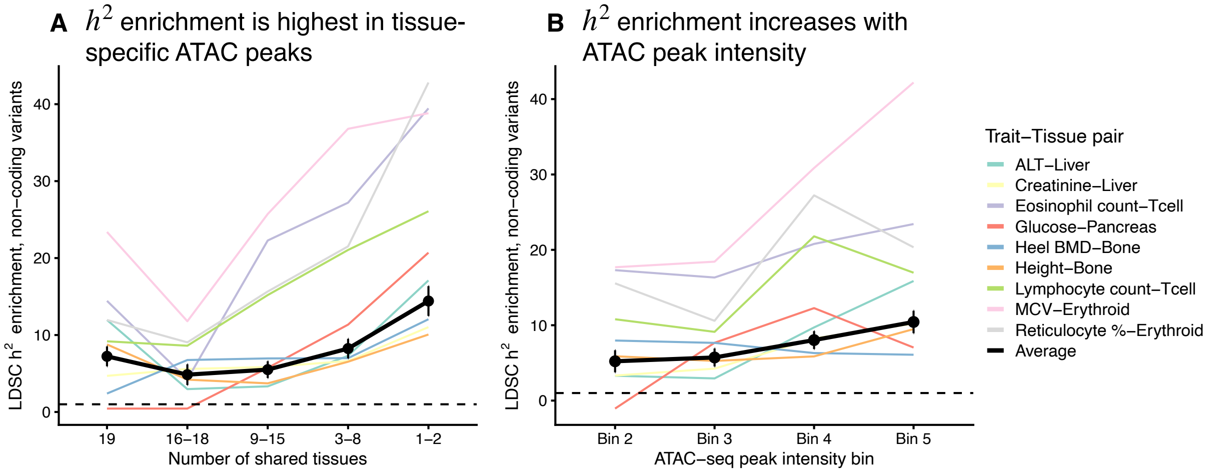

Supplementary Figure S40: **Heritability enrichment increases with ATAC peak tissue specificity and intensity.**

S-LDSC analysis results for 9 trait-tissue pairs. Results are reported in terms of h^2 *enrichment. Variants are binned by the intensity of their ATAC-seq peaks and in how many tissues the ATAC-seq peak is present (Methods). Since the 5 intensity bins are co-linear, we drop the bin 1 annotation and only report results for the remaining bins. These results are from a joint analysis including both ATAC specificity and ATAC intensity bins as covariates. Colored lines are the* h^2 *enrichment estimates for individual trait-tissue pairs. The black line is the inverse variance-weighted average across trait-tissue pairs.*

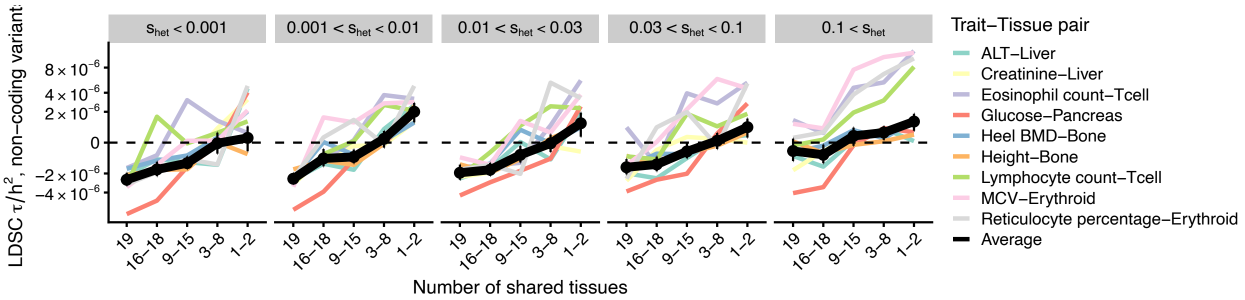

Supplementary Figure S41: **Specificity of ATAC peaks increases heritability explained across constraint bins.**

S-LDSC analysis results for 9 trait-tissue pairs. Result are reported in terms of τ/h^2, a measure of an annotation's effect on proportion of heritability explained. Variants in ATAC-seq peaks are annotated by the combination of the s_{het} value of their closest gene and in how many tissues the ATAC-seq peak is present. In particular, we have 5 s_{het} ranges and 5 ATAC-seq-peak-specificity categories, resulting in 25 distinct annotations, and we estimate a τ for each annotation. The intensity of the ATAC-seq peaks is also included as a covariate (Methods). Colored lines are the τ enrichment estimates for individual trait-tissue pairs. The black line is the inverse variance-weighted average across trait-tissue pairs. Here we facet the plots by s_{het} category. One-sided Z−test p-value $< 7 \times 10^{-5}$ for all comparisons between τ in the most specifically-expressed bin and the last specifically-expressed bin, within each of 5 s_{het} bins.

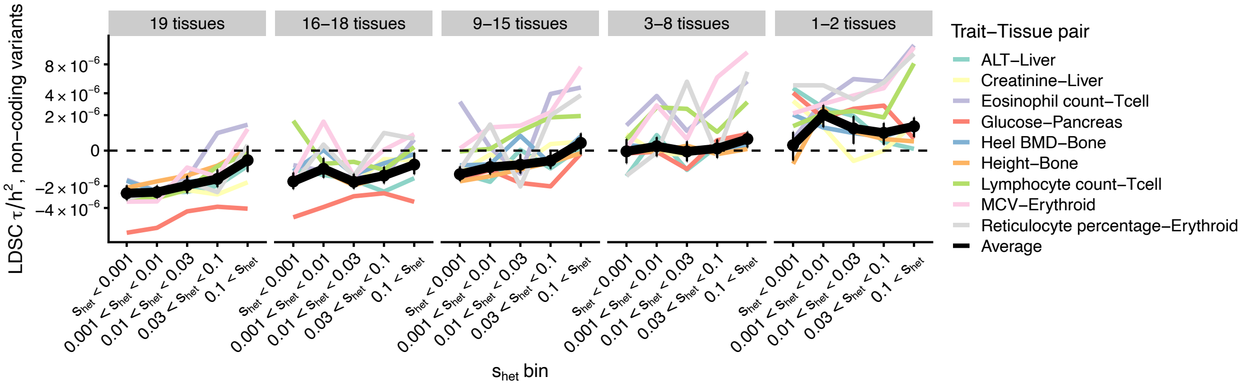

Supplementary Figure S42: **Relationship between constraint and heritability explained across specificity bins.**

S-LDSC analysis results for 9 trait-tissue pairs. Result are reported in terms of τ/h^2, a measure of an annotation's effect on proportion of heritability explained. Variants in ATAC-seq peaks are annotated by the combination of the s_{het} value of their closest gene and in how many tissues the ATAC-seq peak is present. In particular, we have 5 s_{het} ranges and 5 ATAC-seq-peak-specificity categories, resulting in 25 distinct annotations, and we estimate a τ for each annotation. The intensity of the ATAC-seq peaks is also included as a covariate (Methods). Colored lines are the τ enrichment estimates for individual trait-tissue pairs. The black line is the inverse variance-weighted average across trait-tissue pairs. Here we facet the plots by ATAC-seq-peak-specificity category.

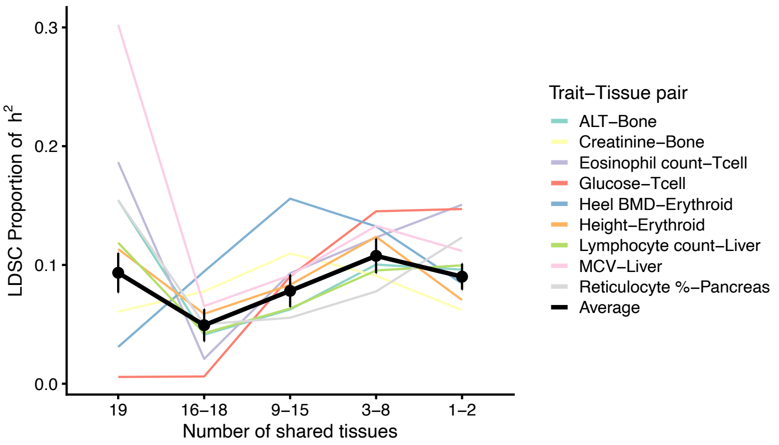

Supplementary Figure S43: **Total proportion of heritability contributed by ATAC peaks of differing tissue specificities.**

S-LDSC analysis results for 9 trait-tissue pairs. Results are reported in terms of the proportion of h^2 *contributed by each bin. Note that this measure depends on both the number of variants within each bin and their per-variant contributions to heritability. As a result, even though variants in the most tissue-specific ATAC peaks contribute more heritability per SNP, and hence are prioritized higher on average, there are fewer such SNPs (0.5% of all SNPs on average across these 9 traits). These SNPs collectively contribute 10.5% of heritability on average across these 9 traits, somewhat less than that contributed by SNPs in ATAC peaks that are shared across all tissues (12.5% averaged across 9 traits), but this is driven by the much larger number of SNPs in such peaks (1.3% of all SNPs on average across these 9 traits. These results are from a joint analysis including both ATAC specificity and ATAC intensity bins as covariates. Colored lines are the* h^2 *enrichment estimates for individual trait-tissue pairs. The black line is the inverse variance-weighted average across trait-tissue pairs.*

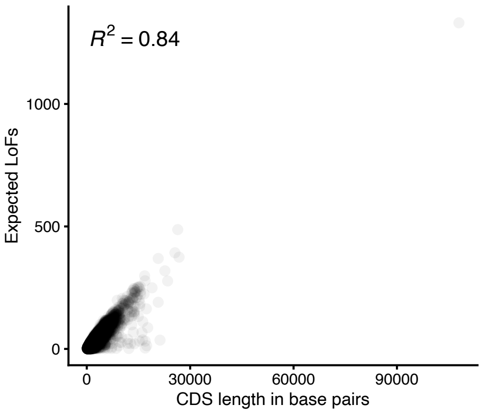

Supplementary Figure S44: **CDS length and expected number of unique LoFs are highly correlated.**

Scatter plot of the expected length in base pairs for the canonical CDS for each gene (Methods) and the expected number of unique LoFs as computed by gnomAD [85]. The overall correlation is high (Pearson's $r = 0.916$*, p-value* $< 10^{-15}$*,* $N = 18{,}067$ *genes).*

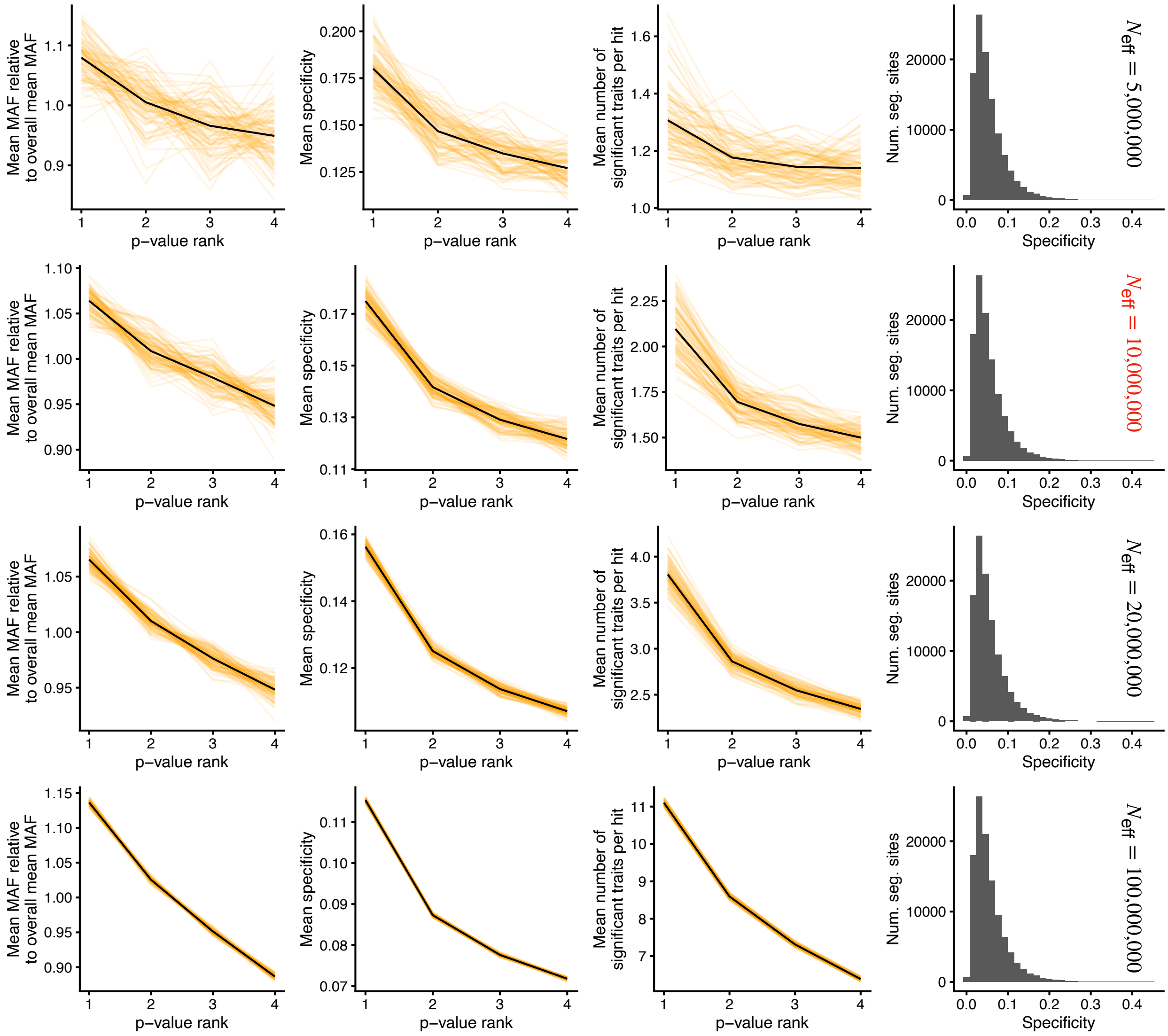

Supplementary Figure S45: **Robustness of apparent pleiotropy to simulation parameter $N_{\mathbf{eff}}$.** *Analogous to Extended Data Figure 3**B-D**, but with varying N_{eff} (see Methods for definition), while holding all other simulation parameters fixed to the values used in the main text. Results from individual population genetic simulations are in orange, and the mean across simulations is in black. The histograms show the distribution of trait specificity, Ψ_V, across segregating sites for a single simulation. N_{eff} does not affect the distribution of effect sizes, and so these are the same across values of N_{eff}. The value of N_{eff} used in the main text is in red.*

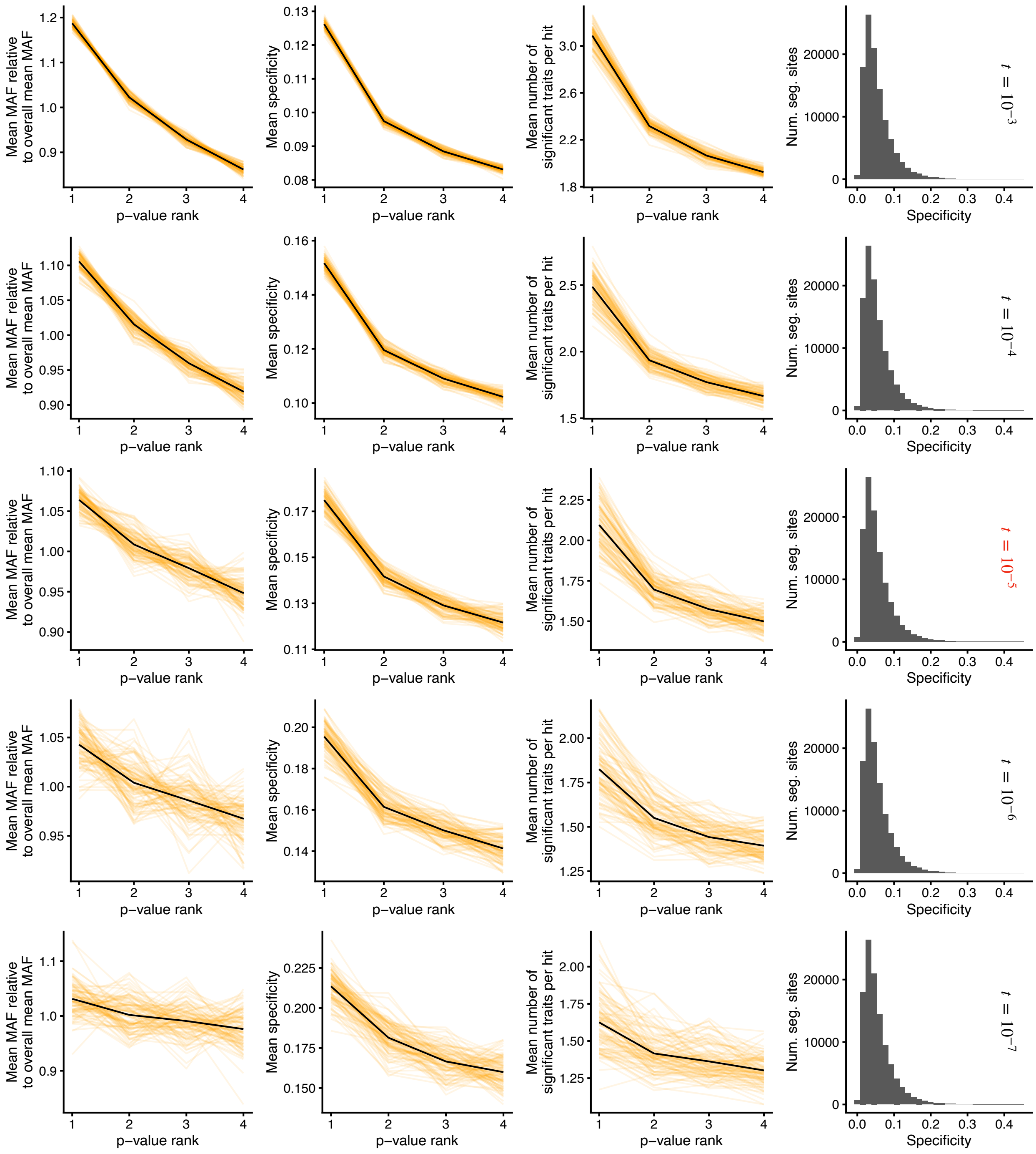

Supplementary Figure S46: **Robustness of apparent pleiotropy to simulation parameter t.** *Analogous to Extended Data Figure 3**B-D**, but with varying t (see Methods for definition), while holding all other simulation parameters fixed to the values used in the main text. Results from individual population genetic simulations are in orange, and the mean across simulations is in black. The histograms show the distribution of trait specificity, Ψ_V, across segregating sites for a single simulation. t does not affect the distribution of effect sizes, and so these are the same across values of t. The value of t used in the main text is in red.*

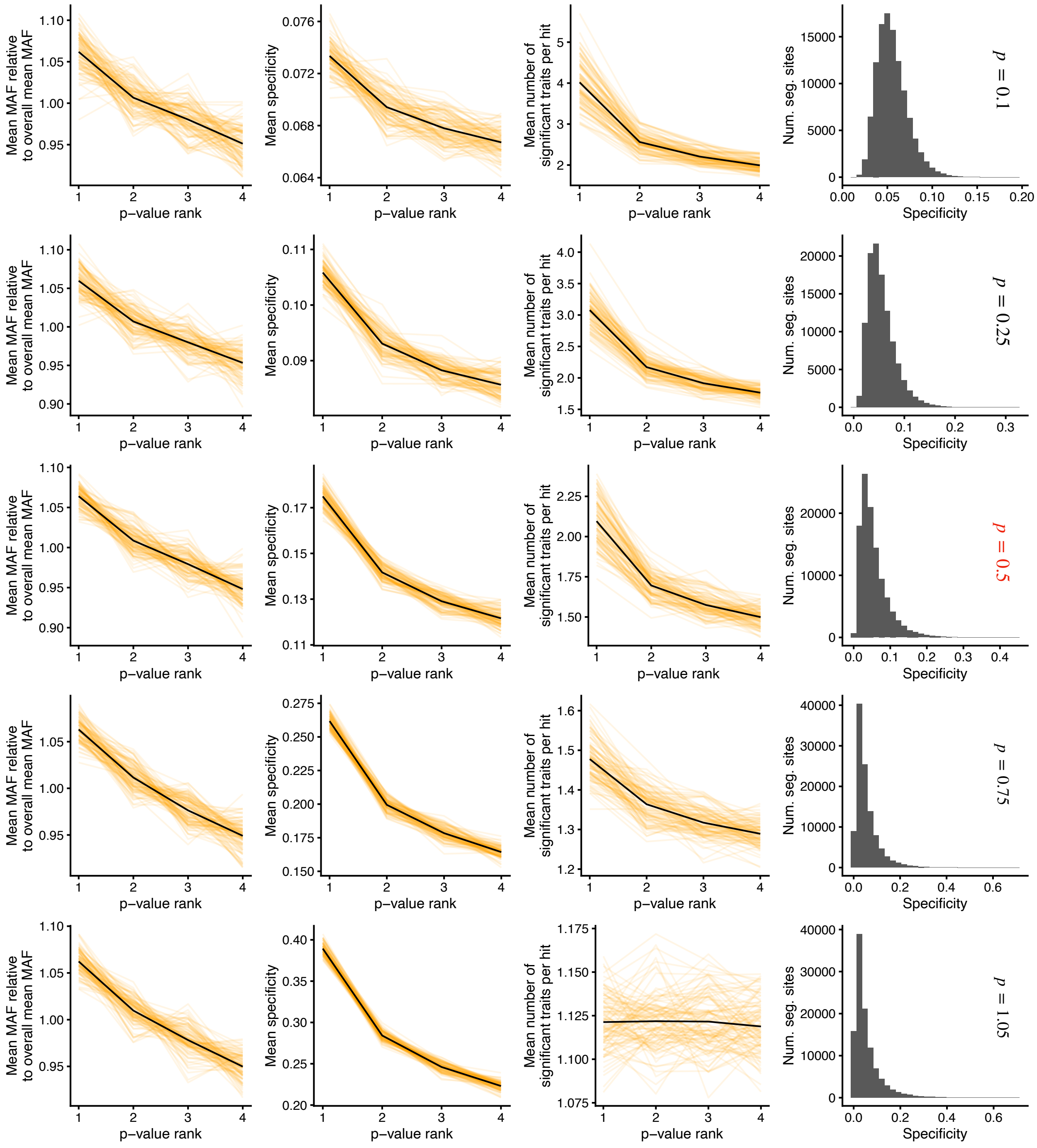

Supplementary Figure S47: **Robustness of apparent pleiotropy to simulation parameter** p. *Analogous to Extended Data Figure 3**B**-**D**, but with varying p (see Methods for definition), while holding all other simulation parameters fixed to the values used in the main text. Results from individual population genetic simulations are in orange, and the mean across simulations is in black. The histograms show the distribution of trait specificity,* Ψ_V*, across segregating sites for a single simulation. The value of p used in the main text is in red.*

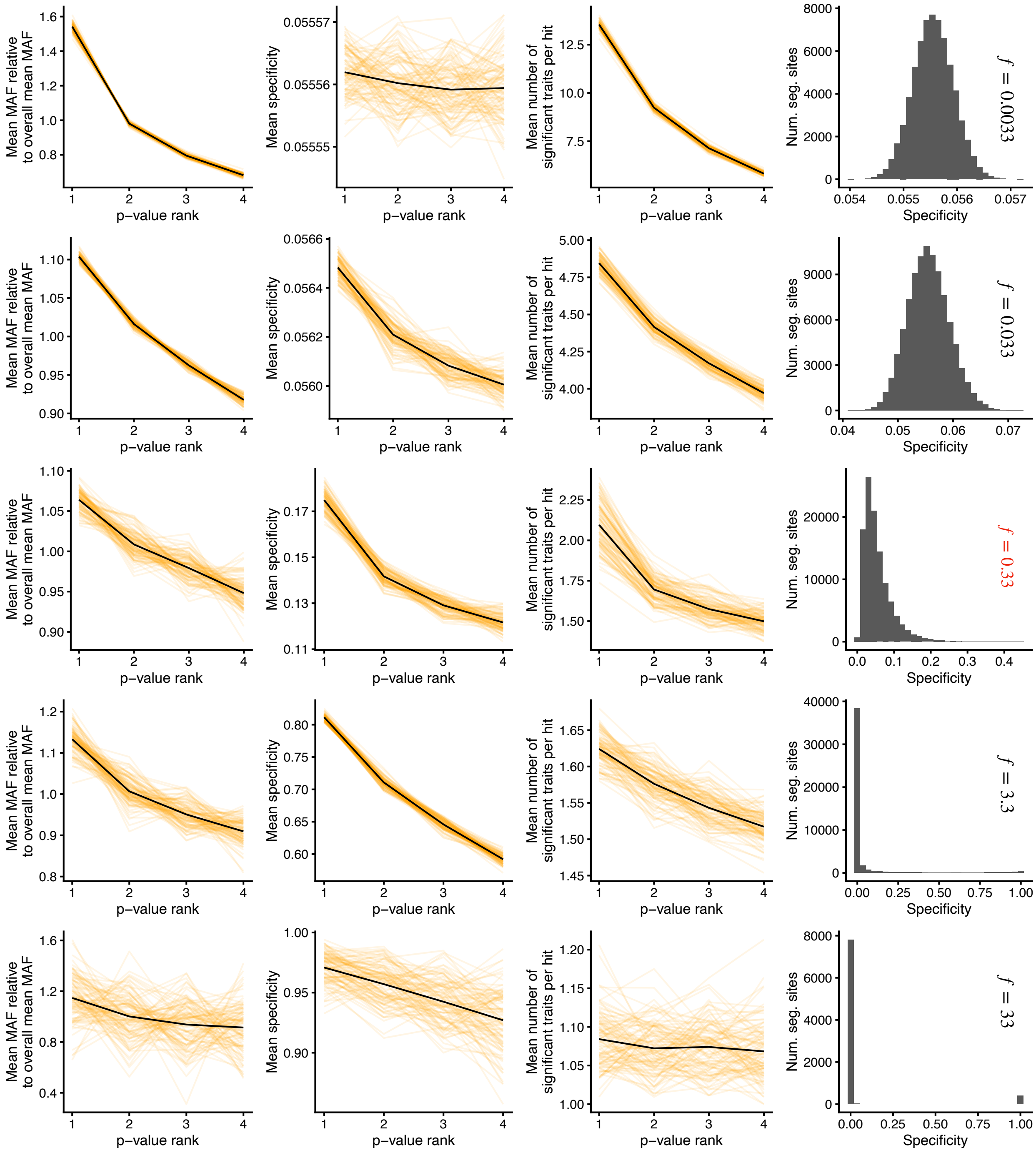

Supplementary Figure S48: **Robustness of apparent pleiotropy to simulation parameter f.** *Analogous to Extended Data Figure 3**B-D**, but with varying f (see Methods for definition), while holding all other simulation parameters fixed to the values used in the main text. Results from individual population genetic simulations are in orange, and the mean across simulations is in black. The histograms show the distribution of trait specificity, Ψ_V, across segregating sites for a single simulation. The value of f used in the main text is in red.*

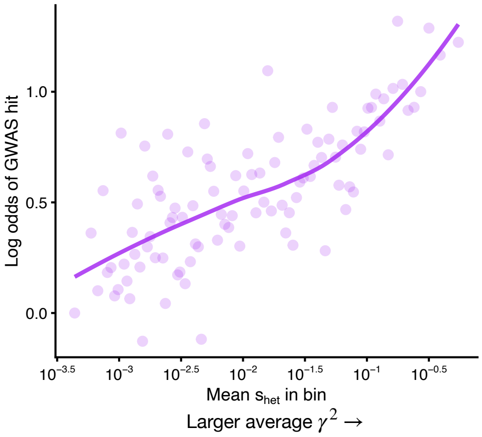

Supplementary Figure S49: **Probability of a variant being a GWAS hit is correlated with s_{het}.** *Logistic regression coefficients for s_{het} percentile categories in a model that predicts whether a variant is a GWAS hit or not including various covariates such as distance to transcription start site (Methods). Each bin contains approximately 184 genes. The trend line is fit using LOESS.*

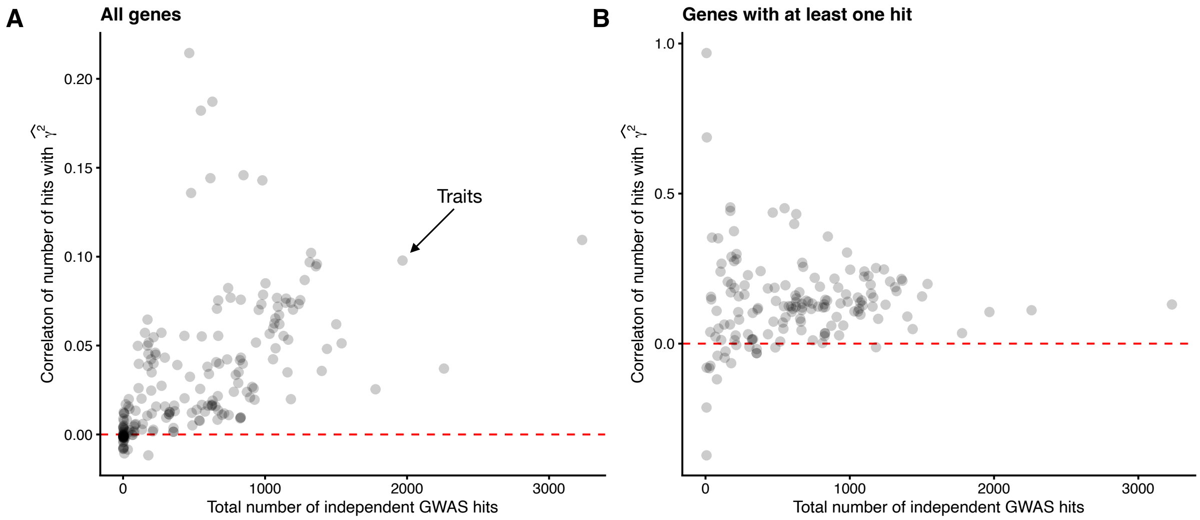

Supplementary Figure S50: **Number of GWAS hits is predictive of γ^2.**

Scatter plots of the correlation across genes between the number of independent GWAS hits and an unbiased estimate of γ^2, $\widehat{\gamma^2}$, against the total number of independent GWAS hits. **A**) *Correlation across all genes. Traits with more independent hits tend to have a higher correlation between number of hits and $\widehat{\gamma^2}$.* **B**) *To make sure that the correlations in panel* **A** *were not driven just by presence or absence of any GWAS hits, we computed correlations between number of GWAS hits and $\widehat{\gamma^2}$ for only those genes with at least one GWAS hit. In both panels, it should be noted that $\widehat{\gamma^2}$ is generally a very noisy estimate of γ^2. This will drive the plotted correlations to be much lower than the true correlation between the number of GWAS hits and the unobserved true values of γ^2.*

Description of Supplementary Tables

Supplementary Table 1: **List of traits and abbreviations used in the study.** *Table of the 209 traits used in this study with the UKB trait IDs, trait names, abbreviations used, tissue to which each trait was linked (if applicable), and an indication of whether or not the trait was included in our subset of 27 genetically uncorrelated traits (Methods).*

Supplementary Table 2: **Genetic and phenotypic correlations between 27 "genetically uncorrelated traits".** *Table of the genetic correlation r_g and phenotypic correlation between each pair of traits among the 27 genetically uncorrelated traits used throughout the paper. Phenotype IDs match those listed in Supplementary Table 1. Estimates were obtained from the Neale Lab (Methods), and phenotypic correlations are missing for 8 of 351 trait pairs.*

A Sensitivity analyses for comparing the genes prioritized by GWAS and burden tests

To ensure that the results presented in Figure 1 did not depend on the particular details of our analysis pipeline, we performed a number of additional analyses. All of the results are qualitatively consistent with those presented in the main text.

To make sure that our results were not driven by the way that we matched GWAS p-values to burden p-values, we tried comparing the minimum GWAS p-value within each approximately independent LD block [86] to the minimum LoF burden test p-value for any gene overlapping that block (Supplementary Figures S4–S7). Similarly, we repeated the analysis in the main text but instead of using LD clumped variants, we used conditionally independent GWAS hits as determined by COJO when defining GWAS loci [87] (Supplementary Figures S8–S10). We also repeated our analysis using a more aggressive threshold when LD clumping prior to constructing GWAS loc (Supplementary Figures S11–S13).

Instead of directly comparing the results of GWAS to LoF burden tests, we also considered first aggregating the GWAS results into gene-level summaries, and used these summaries to prioritize genes. We tried prioritizing genes using MAGMA [88] (Supplementary Figures S14–S16) as well as PoPS [89] (Supplementary Figures S17–S19).

To show that the discrepancy is not due to differences in power between GWAS and LoF burden tests, we repeated our analyses using simulated GWAS of smaller sample sizes (Supplementary Figures S20–S22; see Methods).

To show that the discrepancy is not particular to burden tests based on LoFs, we also considered burden tests based on LoFs and likely damaging missense variants (Supplementary Figures S23–S25).

To show that the discrepancy is not due to burden tests relying on rare variants and GWAS on common variants, we considered only GWAS hits with minor allele frequencies below different thresholds (Supplementary Figures S26–S28).

Finally, perhaps ranking genes based on p-values or statistics derived from p-values, burden tests and GWAS might be more concordant if we ranked genes based on estimated effect size. To test this hypothesis, we ranked loci by the largest magnitude significant effect size instead of the smallest p-value (Supplementary Figures S29–S31) again finding that GWAS and burden tests result in highly discordant rankings.

B A mathematical model of association studies

In this appendix we describe our mathematical model of association studies and clarify the relationship between population, quantitative, and statistical genetics concepts. Our results rely primarily on the work of [90] and [91].

Main theoretical results

We begin with an additive model of phenotypes:

$$\vec{y} = \mathbf{G}\vec{\alpha} + \varepsilon \tag{1}$$

where $y \in \mathbb{R}^n$ is the vector of phenotypes of the n individuals included in the study, $\mathbf{G} \in \mathbb{R}^{n \times p}$ is a matrix containing the centered, but not scaled genotypes of the n individuals at the p causal loci (i.e., $\mathbf{G} = \widetilde{\mathbf{G}} - \frac{1}{n}\mathbf{1}_n\mathbf{1}_n^\top\widetilde{\mathbf{G}}$, where $\widetilde{\mathbf{G}}$ is the matrix of raw genotypes coded as $0, 1, 2$, and $\mathbf{1}_n$ is an n-dimensional vector of 1s), $\vec{\alpha} \in \mathbb{R}^p$ is the vector of the effects of the "1" alleles at each causal locus on the phenotype (usually denoted by β in the literature, but we reserve β for the effect of variants on genes), and $\varepsilon \in \mathbb{R}^n$ is a random vector representing unobserved noise. This additive model is rather simple, but is generally well-supported as a good approximation for many complex traits [92] and is a common assumption across statistical genetics [93–98]. We assume that the causal variants are unlinked in the population, and that p is large enough such that the amount of heritability contributed by any single site is $\ll 1$. We also assume that the phenotype is measured in units so that its variance across individuals is 1.

Before proceeding further, we note that there are several distinct sources of randomness in Equation 1. First, the environmental noise affecting each individual is random, which is made explicit by ε being a random variable. Second, the choice of which individuals are included in the GWAS is random: one could imagine sampling a separate cohort from the same population and obtaining individuals with different genotypes and phenotypes. Third, the genotypes and phenotypes in the population itself are the result of the fundamentally random process of evolution. Note that this is distinct from just obtaining another GWAS cohort. Taking two large random samples from the same population will result in the two cohorts having almost perfectly correlated allele frequencies, but replaying the tape of evolution would result in alleles having completely different frequencies. Throughout, we will try to be clear about which sources of randomness we are averaging over or point out when a particular source of randomness is negligible.

Given our assumptions, it has been shown [90] that conditioned on the GWAS sample, $\widehat{\alpha}_j$, the estimate of the jth marginal effect, α_j, is asymptotically Normally distributed:

$$\widehat{\alpha}_j \sim \mathcal{N}\left(\alpha_j, \|G_j\|_2^{-2}\right), \tag{2}$$

where G_j is the jth column of $\mathbf{G}$ (i.e., the vector of genotypes at the jth causal variant). We emphasize that this approximation relies upon an asymptotic argument, but is highly accurate for traits that are approximately Normally distributed, even for extremely rare variants [99]. While it should be possible to derive a similar approximation to the results of logistic regression applied to case-control data (i.e., based on the asymptotic Normality of maximum likelihood estimators), the point at which those asymptotics provide an accurate approximation is unclear. For binary phenotypes, rare variants can result in the problem of quasi-separability, which is well-known

and causes strong deviations from Normality (see [100] for a thorough discussion). In any case, we only consider inverse rank normal transformed phenotypes, and for these Equation 2 is a good approximation.

Next, note that the standard error of $\widehat{\alpha}_j$ estimated by GWAS is $\|G_j\|_2^{-1}$. Therefore, the z-score, for variant j, $z_j := \widehat{\alpha}_j/\mathrm{SE}(\widehat{\alpha}_j)$, reported by GWAS is also asymptotically Normally distributed:

$$z_j \sim \mathcal{N}\left(\alpha_j\|G_j\|_2, 1\right).$$

Equivalently, we can write z_j as the sum of a constant and a standard Normal random variable, say u_j:

$$z_j \overset{d}{=} \alpha_j\|G_j\|_2 + u_j.$$

We therefore have that the GWAS test statistic, z_j^2 (sometimes referred to as the chi-squared statistic as it is asymptotically chi-squared distributed under a null hypothesis of no effect of the variant on the trait), is

$$z_j^2 \overset{d}{=} \alpha_j^2\|G_j\|_2^2 + 2\alpha_j\|G_j\|_2 u_j + u_j^2. \tag{3}$$

Note that u_j is determined by the environment randomness in ε. Averaging over the randomness in ε, which we denote by $\mathbb{E}_\varepsilon$ we obtain that the expected z_j^2 statistic is

$$\mathbb{E}_\varepsilon z_j^2 = \alpha_j^2\|G_j\|_2^2 + 1. \tag{4}$$

Note that the right-hand side of equation 4 depends on the genotypes of the individuals included in the GWAS. To relate this to population quantities, we assume that the individuals included were sampled randomly from the population (and hence independently from the phenotype), and that the population is at Hardy-Weinberg equilibrium. In such a case, the variance of a randomly sampled genotype with population frequency f_j is $2f_j(1-f_j)$, which can be seen by considering the genotype as the sum of two randomly chosen haploids, which are each independent Bernoulli(f_j) random variables. Then, we see that averaging over this sampling process, which we denote by $\mathbb{E}_{\mathbf{G}}$, results in

$$\begin{aligned}\mathbb{E}_{\mathbf{G}}\alpha_j^2\|G_j\|_2^2 &= \alpha_j^2\mathbb{E}_{\mathbf{G}}\|G_j\|_2^2 \\ &= \alpha_j^2\sum_{i=1}^{n}\mathbb{E}_{\mathbf{G}}\mathbf{G}_{ij}^2 \\ &= 2n\alpha_j^2 f_j(1-f_j),\end{aligned} \tag{5}$$

where the first line follows from our assumption that individuals were chosen independently of the phenotype, and the final follows from the fact that we are considering centered genotypes, so $\mathbb{E}_{\mathbf{G}}\mathbf{G}_{ij}^2 = \mathrm{Var}(\mathbf{G}_{ij})$.

Plugging equation 5 into equation 4 we obtain that averaging over environmental randomness and the individuals included in the GWAS, we obtain

$$\mathbb{E}_{\mathbf{G},\varepsilon} z_j^2 = 2n\alpha_j^2 f_j(1-f_j) + 1. \tag{6}$$

As a result, under our assumptions the average strength of association for the jth variant in GWAS is determined by $2n\alpha_j^2 f_j(1-f_j)$.

By a similar argument, as $n \to \infty$,

$$\operatorname*{plim}_{n\to\infty} \frac{z_j^2}{n} = 2\alpha_j^2 f_j(1-f_j), \tag{7}$$

where plim denotes convergence in probability, and so an infinitely powered GWAS would prioritize variants exactly by $2\alpha_j^2 f_j(1-f_j)$. To make this argument rigorous, note that in equation 3, $\mathbf{G}_{ij}$ is bounded so $\|G_j\|_2$ is $O(\sqrt{n})$ and u_j is $O_p(1)$, so only the $\alpha_j^2\|G_j\|_2^2$ is $O(n)$. Then, again because $\mathbf{G}_{ij}^2$ is bounded, $\|G_j\|_2^2/n$ converges in probability to its expected value, $2f_j(1-f_j)$, by the weak law of large numbers.

From the perspective of population genetics, the frequencies appearing in equations 6 and 7 are the result of evolution, which is itself a random process. As such, we might consider how such sites typically behave under an evolutionary model. In this section, we assume that selection against new mutations is strong enough that we are in the mutation-selection balance regime. See Appendix F for the general case. For the subsequent discussion, we will assume without loss of generality that the "1" allele at each locus is the minor allele.

In this regime, selection is strong enough that the minor allele will be at a low enough frequency such that f_j^2 is essentially negligible, so $f_j(1-f_j) \approx f_j$. Then, $\mathbb{E}f_j = \mu/s_{\text{het}}$, where μ is the mutation rate, and s_{het} is the strength of selection against heterozygotes [101, equation (3.9)]. Intuitively, alleles enter the population at a rate of $2N\mu$, where N is the population size, and are removed from the population at a rate of s_{het} in the roughly $2Nf_j$ copies of the 1 allele that reside in heterozygous individuals. At equilibrium, these forces must cancel, resulting in $f_j \approx \mu/s_{\text{het}}$. Plugging this result into equation 6, we obtain

$$\mathbb{E}z_j^2 = \frac{2n\alpha_j^2\mu}{s_{\text{het}}} + 1, \tag{8}$$

where now the expectation is taken over the environmental randomness, the randomness in the composition of the GWAS cohort, and the evolutionary process.

Finally, selection must be acting upon a variant due to its effects on some phenotypes. In Appendix G we show that under a model of stabilizing selection on T traits measured in appropriate units, $s_{\text{het}} \approx \sum_{t=1}^{T} \alpha_{t,j}^2$, where $\alpha_{t,j}$ is the effect of the jth variant on the tth fitness-relevant trait. Substituting this result in equation 8, we obtain our main result

$$\begin{aligned}\mathbb{E}z_j^2 &= 2n\mu\frac{\alpha_j^2}{\sum_{t=1}^{T}\alpha_{t,j}^2} + 1 \\ &= 2n\mu\Psi_V + 1,\end{aligned} \tag{9}$$

where Ψ_V is the trait specificity of the variant as defined in the main text.

LoF burden tests

To extend these results to LoF burden tests, we note that it has previously been shown that Equation 2 is a good approximation to burden tests for continuous phenotypes that have been inverse

rank normal transformed (like all traits considered here) [99]. The only part that needs modification is our analysis of $\|G_j\|_2^2$, which in burden tests is a "burden genotype" instead of a standard genotype. Suppose there are L potential LoF positions in a gene. An individual's (uncentered) burden genotype for that gene is then 2 if they are homozygous for the LoF allele at any of those L positions, 0 if they are homozygous for the non-LoF allel at all L positions, and 1 otherwise. Let f_ℓ be the population frequency of the LoF allele at position ℓ within the gene. Considering a haplotype chosen randomly from the population, the probability that it does not contain an LoF allele is

$$\mathbb{P}\left\{\text{no LoF at pos. } 1, \text{no LoF at pos. } 2, \ldots, \text{no LoF at pos. } L\right\} \approx \prod_{\ell=1}^{L}(1-f_\ell),$$

where the approximation is reasonable because LoFs tend to be extremely rare [85] and rare variants tend to be essentially independent [102]. We then continue this line of approximation, by noting that if LoFs are rare then any quadratic or higher order terms in the LoF frequencies at one or more positions are negligible. As a result,

$$\prod_{\ell=1}^{L}(1-f_\ell) \approx 1 - \sum_{\ell=1}^{L} f_\ell,$$

and $\sum_{\ell=1}^{L} f_\ell$ can be interpreted as the aggregate frequency of LoF variants. Assuming that this aggregate frequency is also small, we can then assume that it is unlikely to observe an individual with an LoF allele on both of their haplotypes. We can therefore approximate individual i's burden genotype for gene j, $\mathbf{G}_{ij}$, as the sum of two Bernoulli($\sum_{\ell=1}^{L} f_\ell$) random variables. The variance of this sum is then

$$\mathbb{E}_{\mathbf{G}}\mathbf{G}_{ij}^2 \approx 2\sum_{\ell=1}^{L} f_\ell \left(1 - \sum_{\ell=1}^{L} f_\ell\right) \approx 2\sum_{\ell=1}^{L} f_\ell.$$

Using this result in place of equation 5, and noting that we write γ_j for α_j in the case of LoF burden tests, results in

$$\mathbb{E}_{\mathbf{G},\varepsilon} z_j^2 \approx 1 + 2n\gamma_j^2 \sum_{\ell=1}^{L} f_\ell$$

and

$$\operatorname*{plim}_{n\to\infty} \frac{z_j^2}{n} \approx 2\gamma_j^2 \sum_{\ell=1}^{L} f_j.$$

Finally, if we assume each of these L positions are under mutation selection balance with mutation rates μ, then

$$\mathbb{E}z_j^2 \approx \frac{2n\gamma_j^2 \mu L}{s_{\text{het}}} + 1$$

giving our main result for burden tests,

$$\begin{aligned}\mathbb{E}z_j^2 &\approx 2n\mu L \frac{\gamma_j^2}{\sum_{t=1}^{T} \gamma_{t,j}^2} + 1 \\ &= 2n\mu L \Psi_G + 1,\end{aligned}$$

where Ψ_G is the trait specificity of the gene as defined in the main text.

Relationship between z^2, h^2, and $-\log p$-value

We end this section by noting that z_j^2 is closely related to both the heritability explained by that variant, h_j^2, as well as the the $-\log p$-value returned by an association test. We will focus on GWAS in this subsection for clarity, but the results also apply to LoF burden tests.

First, note that h_j^2 is defined (under our assumption of independent causal variants) as $2\alpha_j^2 f_j(1-f_j)$ [101, equation (6.20)]. This is exactly the expected value (averaging over the environmental noise and GWAS cohort composition) of $z_j^2 - 1$ as seen in equation 6. That is,

$$\mathbb{E}_{\mathbf{G},\varepsilon} z_j^2 - 1 = h_j^2,$$

and so ranking variants based on z_j^2 is, in expectation, equivalent to ranking based on contribution to heritability.

Next, note that under a null hypothesis where z has a standard Normal distribution (i.e., the null used in GWAS),

$$\mathbb{P}\left\{z > t\right\} = 1 - \Phi(t) = \Phi(-t),$$

where $\Phi(t)$ is the standard Normal cumulative distribution function. This implies that for $t \geq 0$,

$$\mathbb{P}\left\{z^2 > t^2\right\} = 2\Phi(-t).$$

This means that the reported p-value is related to z^2 by

$$p = 2\Phi\left(-\sqrt{z^2}\right),$$

which implies that

$$z^2 = \left(\Phi^{-1}\left(\frac{p}{2}\right)\right)^2. \tag{10}$$

For small p, Φ^{-1} is asymptotically [103]

$$\Phi^{-1}\left(\frac{p}{2}\right) \approx -\sqrt{\log\left(\frac{4}{2\pi p^2}\right)}. \tag{11}$$

Using the approximation in equation 11 in equation 10 we obtain that for small p-values, p is related to z^2 by

$$z^2 \approx \log\left(\frac{4}{2\pi}\right) - 2\log p \approx -2\log p. \tag{12}$$

Therefore for large values of z^2, it is essentially the same as twice the natural log p-value. In GWAS it is standard to visualize and discuss $-\log_{10} p$-values, but these are just a constant scaling times the natural log p-values:

$$-\log_{10} p = -\frac{\log p}{\log(10)} \approx -0.43\log p \approx 0.22 z^2. \tag{13}$$

Because of these results, we freely switch between discussing average z^2 statistics, contributions to heritability, and $-\log p$-values in the main text.

C A model of context specificity to explain variant specificity

The main goal of this appendix is to build a model of how variants affect phenotypes that incorporates both the context dependence of variants and gene-level pleiotropy. Specifically, we want to provide an analytical expression for *variant specificity*, Ψ_V, in terms of *gene specificity*, Ψ_G, and a term that can be interpreted as "context specificity" under such a model.

Throughout, we will consider a biologically-inspired model of how a variant affects a trait. We assume that a variant affects traits by changing the "activity" of a gene in one or more "contexts", and this gene affects traits by its activity in these "contexts". We consider the "activity" of a gene as the ability of the gene to perform its physiological task in a given context. Similarly, the notion of "context" is completely abstract, but one could think of a context as being a specific cell type or tissue or perhaps an even more specific condition, such as a certain cell type at certain point of development when exposed to a certain stimulus. As such, we would say that a missense variant that totally disrupts protein folding would have a large negative effect on activity across all contexts, while a regulatory variant in a tissue-specific regulatory region may have a positive or negative effect on activity by respectively increasing or decreasing the expression of the gene in that context. Finally, we consider that traits have some impact on fitness, and thus affect the frequency of the variant dependent on that variant's effects on the gene in different contexts and the effects of the gene on different traits in each context.

Our full model is summarized in Figure S51. We assume some number of contexts, C, and a number of traits T. We denote the effect of a variant on the activity of the gene in context c as β_c, and the effect of the gene in context c on trait t as γ_{ct}. That is, β is a property of the variant and γ is a property of the gene. We then define the effect of the variant on trait t as $\alpha_t = \sum_{c=1}^{C} \beta_c \gamma_{ct}$. In Appendix G, we show that under some sensible assumptions the strength of selection in heterozygotes is equal to the sum of squared effects across all traits,

$$s_{\text{het}} = \sum_{t=1}^{T} \left(\sum_{c=1}^{C} \beta_c \gamma_{ct} \right)^2 ,$$

and hence under mutation selection balance, the relevant quantity for the average strength of association under this model is the trait specificity of the variant,

$$\Psi_V = \frac{\left(\sum_{c=1}^{C} \beta_c \gamma_{c1} \right)^2}{\sum_{t=1}^{T} \left(\sum_{c=1}^{C} \beta_c \gamma_{ct} \right)^2}, \tag{14}$$

as we argued in Appendix B.

Analogously, we can consider the trait specificity of the gene, Ψ_G, which under our model is

$$\Psi_G = \frac{\left(\sum_{c=1}^{C} \gamma_{c1} \right)^2}{\sum_{t=1}^{T} \left(\sum_{c=1}^{C} \gamma_{ct} \right)^2}. \tag{15}$$

The crux of this appendix is defining a *context specificity factor*, F_C, that captures how much more specific a variant is than the gene through which it acts. We can implicitly define F_C by

$$\Psi_V = \Psi_G F_C.$$

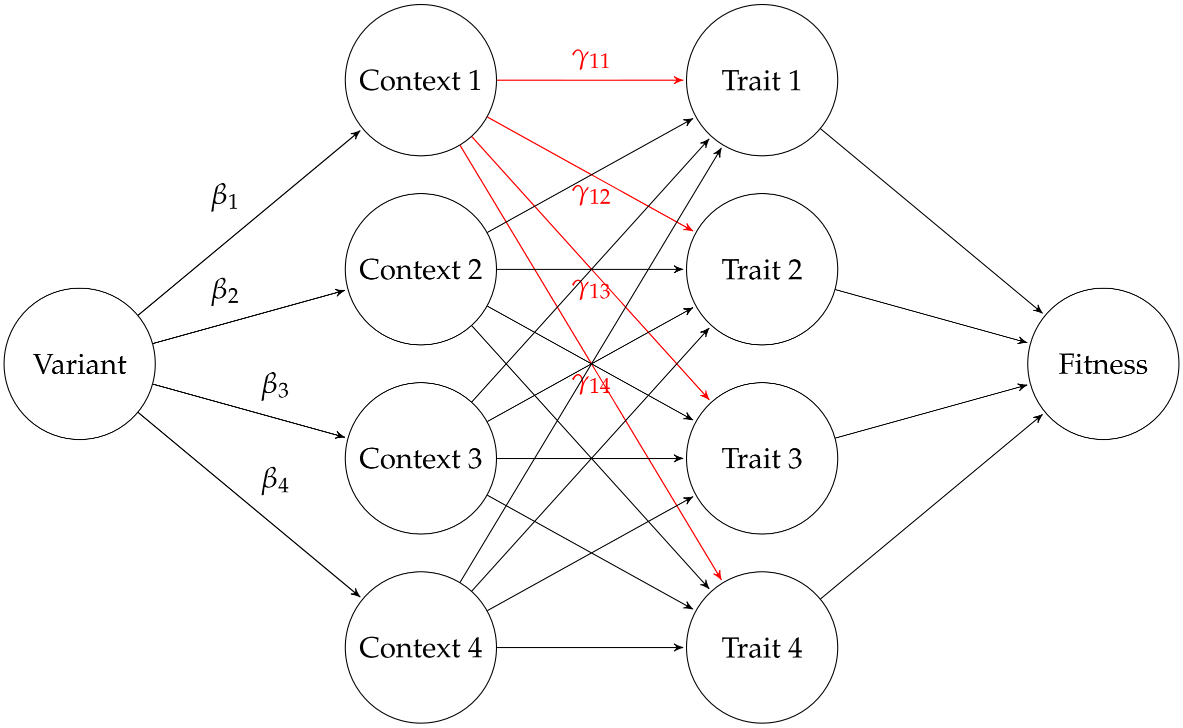

Supplementary Figure S51: The most general model we will consider. A variant has effects in different contexts, and a gene determines how each context affects each trait.

That is,

$$F_C = \frac{\Psi_V}{\Psi_G} = \frac{\left(\sum_{c=1}^{C} \beta_c \gamma_{c1}\right)^2 \sum_{t=1}^{T} \left(\sum_{c=1}^{C} \gamma_{ct}\right)^2}{\left(\sum_{c=1}^{C} \gamma_{c1}\right)^2 \sum_{t=1}^{T} \left(\sum_{c=1}^{C} \beta_c \gamma_{ct}\right)^2}. \tag{16}$$

In this fully general model, the expression for F_C is difficult to interpret. As such, we seek a simpler model to gain some intuition. In contrast to the full model, we will now assume a simpler model where contexts and traits have a one-to-one mapping. That is, each trait is affected exclusively by a single context. We further assume that the gene either has no effect on a given trait, or has an effect of γ, independent of the trait. Similarly, we assume that a variant either has no effect on the activity of a gene in a context, or has an effect of size β, with β being independent of the context. This model is summarized in Figure S52.

Under our toy model the equations for Ψ_V, Ψ_G, and F_C simplify considerably. Let $\mathcal{C}_V$ be the set of contexts for which the variant has non-zero effects on the gene, and let $\mathcal{C}_G$ be the set of contexts in which the gene has non-zero effects on the corresponding traits. Assuming that the variant affects the focal context and the gene has an effect in the focal context on the focal trait, we have

$$\Psi_V = \frac{1}{|\mathcal{C}_V \bigcap \mathcal{C}_G|}.$$

In words, Ψ_V is 1 over the number of contexts where both the variant and gene have an effect. Similarly,

$$\Psi_G = \frac{1}{|\mathcal{C}_G|}.$$

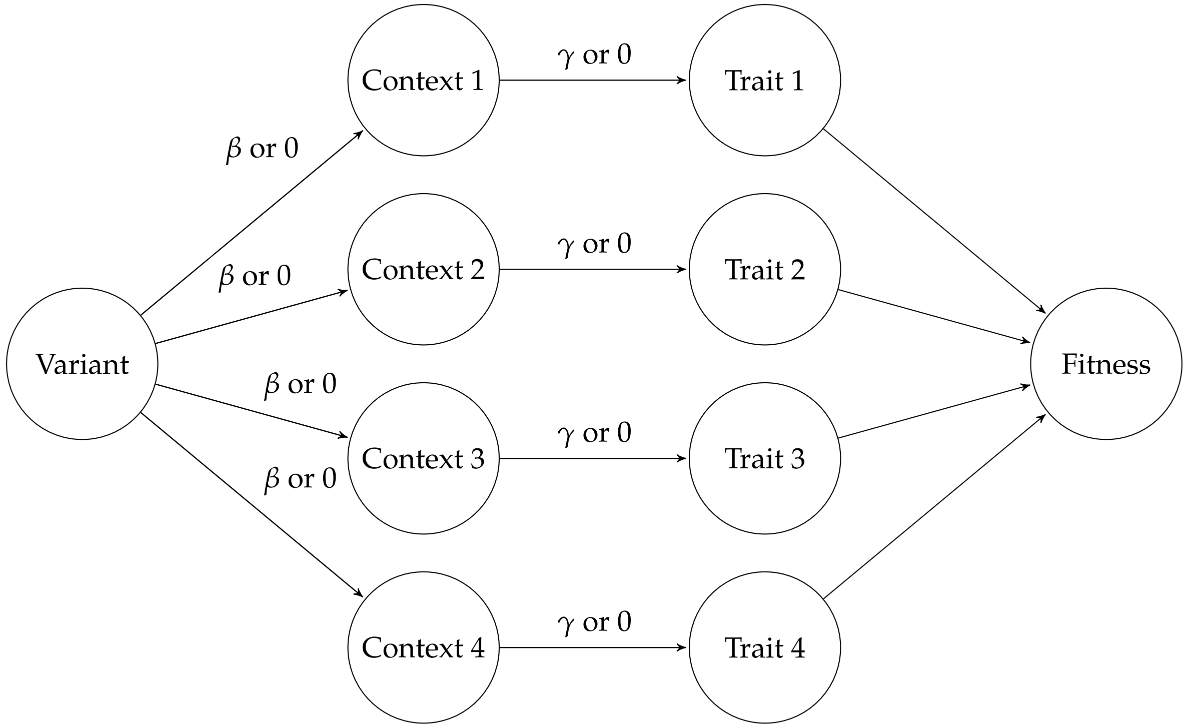

Supplementary Figure S52: The simplest model we will consider. There is a one-to-one correspondence between contexts and traits. Variants either do or do not affect each context, and for a given gene each context may or may not affect its corresponding trait.

Again, Ψ_G is 1 over the number of contexts where the gene has an effect. Equivalently, Ψ_G is 1 over the number of traits that the gene affects.

Finally, we can compute the context specificity factor in this simple model:

$$F_C = \frac{|\mathcal{C}_G|}{|\mathcal{C}_V \bigcap \mathcal{C}_G|},$$

which has a particularly nice interpretation. The inverse, $1/F_C$, is the proportion of trait-relevant contexts that are affected by the variant. That is, we do not care how many contexts the variant affects, we only care how many contexts are affected *where the gene is relevant to traits*. In this sense F_C captures an intuitive measure of context-specificity where we measure context-specificity weighted by how important each context is to any trait.

This simple model makes a number of strong assumptions. We can relax the assumption that all of the effects of the variant on the contexts are either β or 0 and the assumption that the effects of the gene in each context on the corresponding trait is either γ or 0. In particular, we will now consider a model where there is still a one-to-one context-to-trait mapping, but the effect of the variant on context c is β_c and the effect of the gene in context c on trait c is γ_c (note that we only need a single index on γ now because determining the context determines the trait and *vice versa*). We show this model in Figure S53.

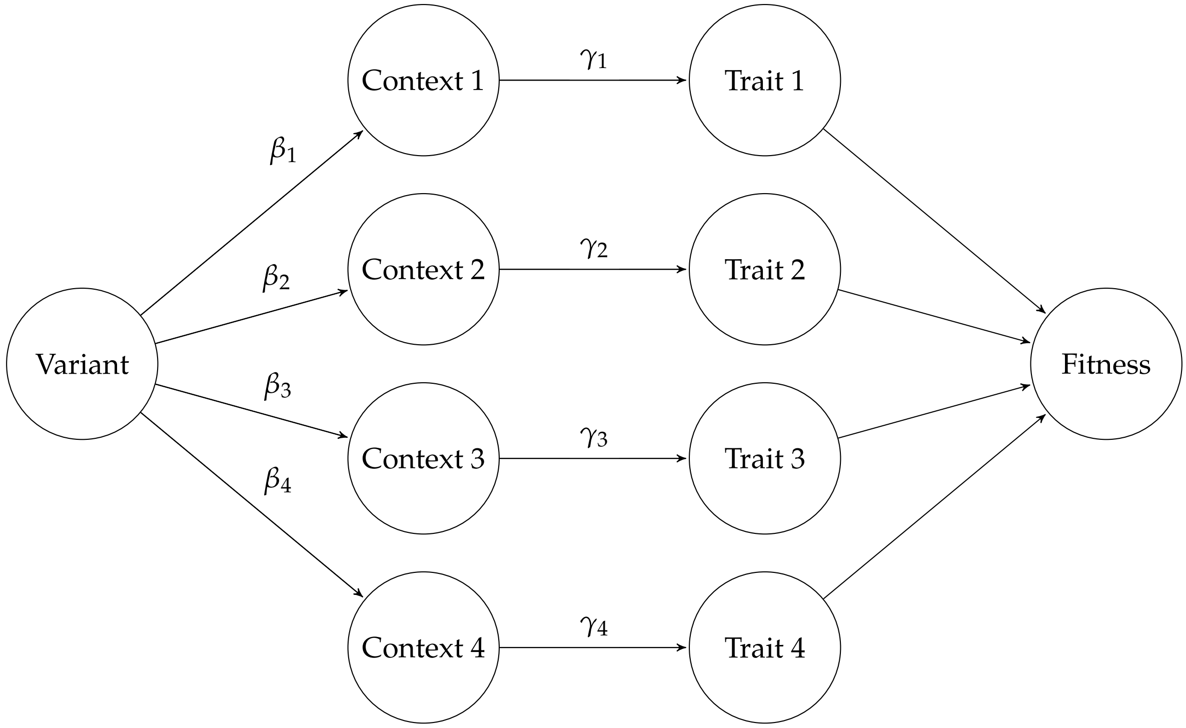

Supplementary Figure S53: A slight relaxation of the simplest model. There is still a one-to-one correspondence between contexts and traits, but now variants can have arbitrary effects on contexts, and genes can have arbitrary effects on the corresponding trait in each context.

Under this model, we then have

$$\Psi_V = \frac{\beta_1^2 \gamma_1^2}{\sum_{c=1}^{C} \beta_c^2 \gamma_c^2}, \tag{17}$$

and

$$\Psi_G = \frac{\gamma_1^2}{\sum_{c=1}^{C} \gamma_c^2}. \tag{18}$$

Substituting these into equation 16, we obtain

$$F_C = \frac{\beta_1^2}{\sum_{c=1}^{C} \left(\frac{\gamma_c^2}{\sum_{c'=1}^{C} \gamma_{c'}^2} \right) \beta_c^2}.$$

This equation is interpretable — it is the effect of the variant on the focal context relative to a weighted average affect across all contexts where the weights for each context are proportional to how much the gene affects a trait in each context.

In both simplified models, the context specificity factor tracks how much a variant affects the context relevant for the focal trait relative to how much it affects all contexts (weighted by their trait relevance). In either case, these models formalize the intuition that by only affecting a subset of contexts, a variant can be substantially more trait specific than the gene through which it acts.

Variants can be less trait specific than the genes through which they act

Our full model (Figure S51) can result in counterintuitive cases where a variant is *less* trait specific than the gene through which it acts. Indeed, even the simplified model shown in Figure S53 can result in cases where $\Psi_V < \Psi_G$. Since this simplified model is a sub-model of the full model, it implies that such examples are feasible in the full model as well. As a numerical example, assume there are two contexts and take $\beta = (1/2, 2)$ and $\gamma = (2, 1/2)$. Plugging these values into Equations 17 and 18 we obtain

$$\Psi_V = \frac{1}{2}$$
$$\Psi_G = \frac{4}{4 + \frac{1}{4}} = \frac{16}{17}.$$

Here we see that the variant evenly affects both traits (and hence is quite non-specific) while the gene very heavily favors the focal trait (and hence is quite specific).

An intuitive explanation for what has happened is that the variant has strong effects on relatively unimportant contexts and weak effects on important contexts. These balance out, and so suddenly contexts that were at very different scales of impact at the gene level become more similar in impact at the variant level. For example, core genes for one trait may have weak effects on other traits when expressed in the wrong context [104]. A variant that hardly affects the important context, but massively perturbs an unimportant context might bring these effects more in line with each other and hence be less specific than the gene.

D LoF burden tests prioritize long genes

For Extended Data Figure 1, we binned genes based on their expected number of unique LoFs, a measure related to μL [85] (but see [105] for theoretical caveats). Empirically, this measure is strongly correlated with coding sequence (CDS) length (Supplementary Figure S44), so we refer to this as "gene length" in the main text.

An interesting consequence of the results presented in Extended Data Figure 1, is that longer genes *appear* more pleiotropic. Longer genes tend to be hits for more traits (Spearman's ρ between mean number of hits across 27 genetically uncorrelated traits and expected number of LoFs = 0.083, p-value $< 10^{-15}$, $N = 18{,}344$ genes). This effect is likely caused by their length increasing their power and not because they actually have larger effects across more traits (Extended Data Figure 1**A**).

We also note that the effect of gene length on burden z^2 is straightforward to correct for in principle. In particular, one could rank genes based on z^2 divided by some measure of μL, such as CDS length or the expected number of unique LoFs. Some caution is warranted, however, as the resulting measure would be noisier for short genes than long genes, and hence ranking by genes by this measure would enrich for the shortest, noisiest genes.

E Genetic drift makes the strongest GWAS hits appear more pleiotropic

Genetic drift drives randomness in minor allele frequencies (MAF), and this randomness in MAF explains an apparent contradiction between our finding that GWAS prioritize trait-specific variants and previous studies that report GWAS hits appearing to be surprisingly pleiotropic [106–108]. Consider performing GWAS on two traits (Extended Data Figure 3**A**). If a variant is trait specific for one trait, then by definition it cannot be trait specific for the other trait. In the absence of other forces, this results in a negative relationship between the strength of association for the two traits. In contrast, if a variant has high MAF, then the GWAS for both traits will be well-powered. All else being equal, randomness in MAF results in a positive relationship between the strength of association for the two traits. Therefore, variants that are highly ranked in one GWAS will be enriched for variants that are trait specific (and hence less likely to be hits for the other trait) but also enriched for variants that have high MAF (and hence are more likely to be hits for the other trait). This explains the supposed contradiction: the top hits for one trait are not actually more pleiotropic on average than other variants, they are simply at higher MAFs and hence better powered across all traits. This is a similar effect to what we saw with gene length for burden tests, where length increases power and causes longer genes to *appear* to be more pleiotropic.

To see if this prediction of our model is corroborated by the UKB GWAS, we compared properties of GWAS hits taken from 27 genetically uncorrelated traits to properties of GWAS hits simulated under our model (Methods). In both cases, we considered all variants that passed the genome-wide significant threshold as hits, and then partitioned hits into four bins based on their p-values, with the strongest hits being in bin 1 and the weakest (but still genome-wide significant) hits being in bin 4.

Our model recapitulates the behavior of the UKB GWAS hits. As predicted by Extended Data Figure 3**A**, the strongest GWAS hits are at higher than average frequencies in both our model and the UKB GWAS hits (Extended Data Figure 3**B**, one-sided Z-test p-value $< 5.62 \times 10^{-9}$ between the mean MAF in the most significant bin and each other bin). As mentioned above, trait specificity is difficult to measure directly, so we cannot assess the trait specificity of the real GWAS hits, but in our simulations, GWAS prioritize trait-specific variants (Extended Data Figure 3**C**). Finally, consistent with previous studies [106–108], we find that the top GWAS hits for one trait are hits for other traits more often than weaker GWAS hits (Extended Data Figure 3**D**, one-sided Z-test p-value $< 1.27 \times 10^{-30}$ between the mean number of significant traits per hit in the most significant bin and each other bin). We reiterate that on average these hits are in fact *more* trait-specific despite being GWAS hits for more traits, and this discrepancy is caused by their higher than expected MAF. The precise details of our simulation model have a quantitative, but not qualitative effect on these results (Methods; Supplementary Figures S45–S48).

F The impact of stabilizing selection on allele frequency in the context of trait specificity and genetic drift

In Appendix B, we derived our results under an assumption of mutation-selection balance, assuming that the strength of selection acting on a variant was proportional to the sum of its squared effects across all traits, $\sum_t \alpha_t^2$. In this Appendix, we extend these results to the case of mutation-selection-drift balance, obtaining the results of Appendix B as a limiting case.

Before beginning, we note that under our model, the effective strength of selection against heterozygotes is $s_{\text{het}} = \sum_t \alpha_t^2$, with the fitness in homozygotes being 1. Meanwhile, we defined the trait specificity of the variant to be $\Psi_V := \alpha_1^2 / \sum_t \alpha_t^2 = \alpha_1^2 / s_{\text{het}}$, where we assume α_1 is the effect on the focal trait. As such, specifying two of s_{het}, Ψ_V, or α_1^2 determines the third. In particular, $s_{\text{het}} = \alpha_1^2 / \Psi_V$.

As discussed in Appendix B, the expected strength of association in GWAS is closely related to the heritability contributed by the variant,

$$h^2 = 2\alpha_1^2 f(1-f).$$

To understand the typical contribution of a variant, we need to compute the expected value of $2f(1-f)$, which depends on s_{het}.

It has long been well-understood [91, 109, 110] that under our stabilizing selection setup, the evolutionary dynamics of an allele are approximately equivalent to those of an underdominant model, where individuals homozygous for either allele have fitness 1, but heterozygous individuals have fitness $1 - s_{\text{het}}$. We note that under a model of stabilizing selection, heterozygous individuals are not actually less fit than homozygotes on average — it is something of a mathematical coincidence that the evolutionary dynamics under stabilizing selection are equivalent to such a model.

In an underdominant model, the allelic dynamics are described by the stochastic differential equation (SDE):

$$df_t = [2Ns_{\text{het}} f_t(1-f_t)(2f_t - 1) + 2N\mu(1-2f_t)]\, dt + \sqrt{f_t(1-f_t)} dW_t,$$

where N is the effective population size, f_t is the frequency at time t, W_t is a Wiener process (Brownian motion), and time is measured in units of $2N$ generations. The properties of the stationary distribution of this SDE are well-understood [91, 109, 110]. In particular, it has been shown that under the stationary distribution of this model,

$$\mathbb{E}[2f(1-f)] = \frac{4N\mu M\left(\frac{1}{2}, 4N\mu + \frac{3}{2}, Ns_{\text{het}}\right)}{2\left(4N\mu + \frac{1}{2}\right) M\left(\frac{1}{2}, 4N\mu + \frac{1}{2}, Ns_{\text{het}}\right)}, \tag{19}$$

where $M(\cdot, \cdot, \cdot)$ is the confluent hypergeometric function. In practice, this can be accurately computed numerically for small to moderate values of Ns_{het} using `scipy.special.hyp1f1` [111] in Python or `BAS::hypergeometric1F1` [112] in R. For large values of Ns_{het} we found these functions to be numerically unstable. For large values of Ns_{het}, we rely on the asymptotic approximation of the confluent hypergeometric function [113, (13.7.1)],

$$M(a, b, z) \sim \frac{\Gamma(b)}{\Gamma(a)} \left(e^z z^{a-b}\right)$$

where Γ is the Gamma function. After some algebra and noting that $\Gamma(x+1)/\Gamma(x) = x$, this results in

$$\frac{4N\mu M\left(\frac{1}{2}, 4N\mu + \frac{3}{2}, Ns_{\text{het}}\right)}{2\left(4N\mu + \frac{1}{2}\right) M\left(\frac{1}{2}, 4N\mu + \frac{1}{2}, Ns_{\text{het}}\right)} \sim \frac{4N\mu}{2\left(4N\mu + \frac{1}{2}\right)} \frac{\Gamma(4N\mu + \frac{3}{2})}{\Gamma(4N\mu + \frac{1}{2})} (Ns_{\text{het}})^{-1} = \frac{2\mu}{s_{\text{het}}},$$

exactly recovering the expectation of $2f(1-f)$ under mutation-selection balance.

We can also consider the behavior for small values of s_{het}, where [113, (13.2.2)]

$$M(a, b, z) = 1 + O(z).$$

In this case we obtain

$$\mathbb{E}[2f(1-f)] = \frac{4N\mu}{2\left(4N\mu + \frac{1}{2}\right)} \left(1 + O(Ns_{\text{het}})\right),$$

which indicates that the expected value of $2f(1-f)$ is approximately independent of Ns_{het} for values of $Ns_{\text{het}} \ll 1$, and is (up to terms of order $O(Ns_{\text{het}})$) equal to the equilibrium expected heterozygosity under mutation-drift balance in the biallelic case with symmetric mutation (e.g., as can be derived from [114, Equation 6.6]). This is consistent with population genetics intuition of these sites being effectively neutral, and hence behaving approximately the same as neutral variants regardless of the precise values of their selection coefficients.

We end with a discussion of the behavior of the expected value of h^2 as a function of α_1^2 and Ψ_V. To emphasize this reliance of $\mathbb{E}h^2$ on these quantities, we will write $\mathbb{E}h^2(\alpha_1^2, \Psi_V)$. Writing $\mathbb{E}\pi(s_{\text{het}})$ for $\mathbb{E}[2f(1-f)]$ to emphasize that the expected value of the heterozygosity (π) depends on s_{het}, and recalling that $s_{\text{het}} = \alpha_1^2/\Psi_V$, we have

$$\mathbb{E}h^2(\alpha_1^2, \Psi_V) = \alpha_1^2 \mathbb{E}\pi\left(\frac{\alpha_1^2}{\Psi_V}\right). \tag{20}$$

Our above results show that for small α_1^2/Ψ_V, regardless of Ψ_V,

$$\mathbb{E}h^2(\alpha_1^2, \Psi_V) \approx \frac{4n\mu\alpha_1^2}{2(4N\mu + \frac{1}{2})} \qquad \text{(for } \alpha_1^2/\Psi_V \ll 1\text{)},$$

and so expected heritability in this regime is driven essentially solely by the effect size. In contrast, when α_1^2/Ψ_V is large, our results imply

$$\mathbb{E}h^2(\alpha_1^2, \Psi_V) \approx 2\mu\Psi_V, \qquad \text{(for } \alpha_1^2/\Psi_V \gg 1\text{)},$$

implying that the expected strength of association is determined only by Ψ_V in this regime. This regime corresponds to strong selection, and a variant can be in this regime in multiple ways. For example, it could have small effects but affect many traits, in which case ($\Psi_V \ll \alpha_1^2$), or it could have an extremely large effect on the focal trait ($\alpha_1^2 \gg 1$).

Finally, using a technical lemma, Lemma .1, that we prove below, we can show that for any $\mu > 0$, $\pi(s_{\text{het}})$ is a strictly decreasing function of s_{het}. This result is extremely intuitive as it says that as the effective strength of selection against heterozygotes increases, the expected heterozygosity decreases. This in turn implies that for fixed α_1^2, $\pi(\alpha_1^2/\Psi_V)$ is a strictly *increasing* function of Ψ_V.

Finally, if we hold α_1^2 fixed, we see that the only dependence on Ψ_V in equation 20 is via $\pi(\alpha_1^2/\Psi_V)$. This implies that for fixed α_1^2, $\mathbb{E}h^2(\alpha_1^2, \Psi_V)$ is a strictly increasing function of Ψ_V.

While this result may seem technical, it has a simple interpretation: among all variants with the same trait importance, GWAS will, on average, rank the most trait-specific variants the most highly. Thus, while we focused on the role of trait specificity in the mutation-selection balance regime in the main text, trait specificity plays a key role in association study power across the entire range of effect sizes.

Technical lemma

Lemma .1. *Suppose $b \geq a > 0$. Then,*

$$\frac{M(a, b+1, z)}{M(a, b, z)}$$

is a strictly decreasing function of z on $z > 0$.

Proof. Our proof relies on a technical lemma [115, Lemma 2.1], that states that if all of the following hold:

- $f(x)$ and $g(x)$ can be represented as the series $\sum_{k=0}^{\infty} f_k x^k$ and $\sum_{k=0}^{\infty} g_k x^k$ respectively
- Both series converge on $-R < x < R$
- $g_k > 0$ for all k
- $\left\{\frac{f_k}{g_k}\right\}_{k=0}^{\infty}$ is a decreasing sequence

then $f(x)/g(x)$ is a strictly decreasing function of x on the interval $(0, R)$.

To use this lemma, we use the series representation [113, (13.2.2)],

$$M(a, b, z) = \sum_{k=0}^{\infty} \frac{(a)_k}{(b)_k k!} z^k,$$

where $(c)_k := c(c+1)\cdots(c+k-1)$ is the Pochammer symbol, with the convention that $(c)_0 := 1$. It can be easily shown that this series is convergent for all z by noting that $(a)_k/(b)_k \leq 1$ by assumption that $a \leq b$ and comparing terms to the everywhere convergent series for $\exp z$. Similar considerations show that $M(a, b+1, z)$ is everywhere convergent.

We now write

$$f_k = \frac{(a)_k}{(b+1)_k k!},$$

and

$$g_k = \frac{(a)_k}{(b)_k k!}$$

for the coefficients of z^k in the series representations of $M(a, b+1, z)$ and $M(a, b, z)$ respectively. To use the lemma, we need to prove that $g_k > 0$ for all k and that the ratios f_k/g_k are decreasing

in k. That $g_k > 0$ for all k is trivial, as each g_k is a ratio of positive integers. Now, note that

$$\frac{f_k}{g_k} = \frac{(b)_k}{(b+1)_k} = \frac{b}{b+k},$$

which is strictly decreasing in k. Applying [115, Lemma 2.1] completes the proof. □

G Connection between effect sizes and fitness under stabilizing selection

In this Appendix, we discuss the assumptions under which we may assume that the effective strength of selection acting against a variant, s_{het}, is the sum of that variant's trait importances, $\sum_t \alpha_t^2$.

Our starting point is an arbitrary fitness function, w, which maps a vector of trait values, $\mathbf{t}$, to a fitness. That is, $w(\mathbf{t})$ specifies the average fitness of an individual with trait values $\mathbf{t}$. We need some mild, technical assumptions on w — for our purposes it is sufficient that w have an isolated local maximum, and that w be twice differentiable at that maximum. Without loss of generality, we may assume that $w(\mathbf{t})$ has a local maximum at $\mathbf{t} = 0$ by redefining $\mathbf{t}$ as $\mathbf{t} - \mathbf{t}^*$ where $\mathbf{t}^*$ is a local optimum of w. We then assume that natural selection is strong enough so that most individuals have traits that are close to the optimum. More precisely, we will assume that the trait values we are interested in are close enough to zero so that terms smaller than $\|\mathbf{t}\|_2^2$ are negligible. As such, we may perform a multivariate Taylor expansion of our fitness function around 0:

$$\begin{aligned} w(\mathbf{t}) &= w(0) + \langle \nabla w(\mathbf{s})|_{\mathbf{s}=0}, \mathbf{t}\rangle + \mathbf{t}^\top \left(\nabla^2 w(\mathbf{s})|_{\mathbf{s}=0}\right) \mathbf{t} + o\left(\|\mathbf{t}\|_2^2\right) \\ &= w(0) + \mathbf{t}^\top \left(\nabla^2 w(\mathbf{s})|_{\mathbf{s}=0}\right) \mathbf{t} + o\left(\|\mathbf{t}\|_2^2\right) \\ &\approx w(0) + \mathbf{t}^\top \left(\nabla^2 w(\mathbf{s})|_{\mathbf{s}=0}\right) \mathbf{t}, \end{aligned}$$

where the second line follows from the fact that 0 is an optimum so the gradient of w evaluated at 0 is 0.

Defining $\mathbf{H} := \nabla^2 w(\mathbf{s})|_{\mathbf{s}=0}$ as the Hessian (i.e., matrix of second derivatives) of w at 0, we obtain that the fitness consequence of having a set of traits $\mathbf{t}$ is $-\mathbf{t}^\top \mathbf{H}\mathbf{t}$. Note that since we are at an isolated local maximum, $\mathbf{H}$ must be negative definite, which just means that $\mathbf{v}^\top \mathbf{H}\mathbf{v} < 0$ for any nonzero $\mathbf{v}$.

This is exactly the setting of [91] (under certain assumptions discussed below), so we obtain that if a variant causes a change in phenotypes of $\alpha = (\alpha_1, \ldots, \alpha_T)$ then that results in evolutionary dynamics equivalent to underdominance with a selection coefficient proportional to

$$s_{\text{het}}(\alpha) \propto -\alpha^\top \mathbf{H}\alpha. \tag{21}$$

Recently [116], it has been shown that the assumption of independent sites used in [91] to derive equation 21 is incompatible with the Bulmer Effect [117,118] where variants tend to be in slightly negative LD with other variants that affect the trait in the same direction even if the variants are physically unlinked. Accounting for these subtleties can be well-approximated by changing the constant of proportionality in equation 21, and does not affect our results here [116, equation (43)].

Here we make our first transformation — we absorb the constant of proportionality into α. That is, we may scale all of the traits by some constant to make the above proportionality an equality:

$$s_{\text{het}}(\alpha) = -\alpha^\top \mathbf{H}\alpha.$$

So far we have subtracted a constant from all of our traits and then scaled them by a constant. This does not fundamentally change any of the traits we have measured.

Now, expanding the right-hand side of equation 21 we obtain

$$\alpha^\top \mathbf{H}\alpha = \sum_{i=1}^{T}\sum_{j=1}^{T} \alpha_i \alpha_j \mathbf{H}_{ij}.$$

The cross terms make downstream analysis difficult. Furthermore, the constraint that $\mathbf{H}$ be negative definite implies nontrivial constraints on the values that the entries of $\mathbf{H}$ may take. To simplify further analysis, we would like to get rid of these cross terms without changing our interpretation of the focal trait (say the first dimension of $\mathbf{t}$). The rest of this Appendix will be devoted to accomplishing that task.

Since $\mathbf{H}$ is negative definite, we may define an inner product by

$$\langle \mathbf{u}, \mathbf{v} \rangle_{\mathbf{H}} := -\mathbf{u}^\top \mathbf{H} \mathbf{v}.$$

Note that this is different from the usual Euclidean inner product, but we may prove that it is inner product by noting that

$$\langle \mathbf{u}, \mathbf{v} \rangle_{\mathbf{H}} = -\mathbf{u}^\top \mathbf{H} \mathbf{v} = -\left(\mathbf{u}^\top \mathbf{H} \mathbf{v}\right)^\top = -\mathbf{v}^\top \mathbf{H}^\top \mathbf{u} = -\mathbf{v}^\top \mathbf{H} \mathbf{u} = \langle \mathbf{v}, \mathbf{u} \rangle_{\mathbf{H}}$$

by the symmetry of the Hessian. This shows that our putative inner product is symmetric. Next, note that for scalars a and b,

$$\langle a\mathbf{u} + b\mathbf{w}, \mathbf{v} \rangle_{\mathbf{H}} = -\left(a\mathbf{u} + b\mathbf{w}\right)^T \mathbf{H}\mathbf{v} = a\left(-\mathbf{u}^\top \mathbf{H}\mathbf{v}\right) + b\left(-\mathbf{w}^\top \mathbf{H}\mathbf{v}\right) = a\langle \mathbf{u}, \mathbf{v} \rangle_{\mathbf{H}} + b\langle \mathbf{w}, \mathbf{v} \rangle_{\mathbf{H}}$$

which shows that our putative inner product is linear. Finally for $\mathbf{v} \neq 0$

$$\langle \mathbf{v}, \mathbf{v} \rangle_{\mathbf{H}} = -\mathbf{v}^\top \mathbf{H} \mathbf{v} > 0$$

by the negative definiteness of $\mathbf{H}$ showing that our inner product is positive definite. These three properties define an inner product, so $\langle \cdot, \cdot \rangle_{\mathbf{H}}$ is a bonafide inner product.

Now, since this is an inner product, we may perform the Gram-Schmidt process with respect to this inner product starting with the vector $(1, 0, 0, \ldots)$ which corresponds exactly to the focal trait in the original coordinate system. The output of the Gram-Schmidt process is a new basis, $\mathbf{w}_1, \ldots, \mathbf{w}_T$ such that

$$\langle \mathbf{w}_i, \mathbf{w}_j \rangle_{\mathbf{H}} = \begin{cases} 1 & \text{if } i = j \\ 0 & \text{if } i \neq j \end{cases}$$

and $\mathbf{w}_1 \propto (1, 0, 0, \ldots)$. These new vectors describe how to change the coordinates of a vector from the original coordinate system to a new coordinate system that is orthonormal with respect to our inner product. In our case, we can think of coordinates as traits. The first coordinate remains unchanged (up to a rescaling that is independent of the other coordinates) from the original coordinate system to the new coordinate system, but the remaining coordinates will be linear combinations of more than one coordinate. This means that in our new coordinate system, we still have the focal trait, but then define new traits in terms of linear combinations of the original traits. By orthonormality, we see that if we write α in this new coordinate system,

$$\alpha^\top \mathbf{H} \alpha = -\sum_{t=1}^{T} \alpha_t^2,$$

and thus,

$$s_{\text{het}}(\alpha) = \sum_{t=1}^{T} \alpha_t^2.$$

Ultimately, this means that we may define our traits such that the first dimension is a centered and scaled version of the original first trait dimension (centered so its optimum is zero, scaled so that a unit change of the trait — *holding all other transformed traits constant* — has a unit selection coefficient). The remaining dimensions get scrambled (including with the first original trait dimension), meaning that they are each some linear combination of the original traits.

A subtle point here is that in this coordinate system, the non-focal traits may be in part determined by the focal trait, so we need to be careful in interpreting pleiotropy. For example if we had traits that were weight and $1/\text{height}^2$, we could change the coordinate system to instead be weight and BMI. When we talk about a variant that is specific to weight in this new coordinate system, that variant must not affect BMI. But in the original coordinate system, the variant is necessarily not specific to weight because to keep BMI fixed, it must affect both height and weight.

To derive our results, we relied on the argument presented in [91]. That argument requires the trait to be sufficiently polygenic that the genetic component of an individual's phenotype is approximately Gaussian distributed. It also requires that a new mutation has a random effect, and that that effect is isotropic in our final scaled and rotated trait space. That is, conditioned on its total squared effect, the effect of a new mutation is equally likely to point in any direction of trait space. There are also a number of additional assumptions that are generally met in practice or do not substantially affect the interpretation of the results. See [91, Supplementary Note Sections 4 and 5].

To summarize, if we choose our coordinate system carefully (i.e., choose the correct set of traits and how to measure them), then under fairly general models of stabilizing selection we may obtain $s_{\text{het}} \approx \sum_t \alpha_t^2$. In practice we do not know the fitness function, and so we cannot determine the proper rotation and scaling of trait space to obtain this result. As a result, in the main text, we assume that the traits we consider are such that this result holds. This is obviously a gross approximation, but seems to work surprisingly well in practice, for example in Figure 3**C**. It would be an interesting line of future work to try to estimate $\mathbf{H}$ (or equivalently learn the proper rotation and scaling of trait space).

H LoF burden tests prioritize long genes

In the main text we considered prioritizing genes by on p-value. This leaves open the possibility that other summaries of association studies (e.g., the unbiased estimates of trait importance $\widehat{\alpha^2}$ for GWAS or $\widehat{\gamma^2}$ for LoF burden tests) would be better at ranking genes by importance. Recalling our notation of variants effect on gene being β, we immediately see that ranking genes by importance using GWAS data is not possible without additional information: the relationship between any estimate of α and trait importance will depend on the unknown value of β. This may explain some of the discrepancy we observed between how loci are prioritized by GWAS and burden tests when using the largest significant absolute effect size from each association method. It may also explain why this discrepancy holds even when focusing on loci with both significant burden hits and GWAS hits (Supplementary Figures S29–S31).

In principle, if an LoF burden test is infinitely well powered, then ordering genes by $\widehat{\gamma^2}$ would prioritize genes based on trait importance. At current sample sizes, however, the estimated $\widehat{\gamma^2}$ are noisy enough that the top genes contain many false positives. For example, among the 10 genes with the largest $\widehat{\gamma^2}$ for standing height, 4 are consistent with having no effect on height (all Bonferroni adjusted p-values > 0.62). Restricting to only genome-wide significant genes would eliminate these false positives, but our arguments in the main text show that this would result in the exclusion of genes that are not sufficiently trait-specific, introducing false negatives.

Additional false negatives could come from genes where LoFs are extremely deleterious, where LoF burden tests may never be well powered no matter the sample size. Missense variants could be better tolerated in these genes, and hence association statistics from individual missense variants may provide an avenue for estimating γ, but this would require accurate estimates of β to disentangle the effect of the variant on the gene from the effect of the gene on the trait. In the future, improved estimates of the effects of missense variants on protein function (e.g., [119–121]) may make this an attractive approach for estimating γ.

I Violations of model assumptions when estimating trait importance from association studies

In this Appendix we discuss various caveats and extensions of the model and results presented in the main text around Figure 5.

In particular, in the main text we assumed that genes differ only in their importance. Trait specificity also plays a role, with more trait-specific variants flattening at a larger contribution to heritability. Furthermore, like in LoF burden tests, we expect the total contribution to heritability for a gene to also depend on its mutational target size, with longer genes and genes with more regulatory elements generally contributing more to heritability all else being equal. The precise relationship between γ^2 and various gene-wide measures of aggregate association signal will depend on the unknown relationship between the mutational target sizes for variants of different β and Ψ_V and a gene's γ and Ψ_G.

We also clarify the role of the assumption that the effect of a variant on a trait, α, is $\beta\gamma$ where β is the effect of the variant on the gene and γ is the effect of the gene on the trait. To be more concrete, we can imagine that for expression-altering variants, β is the expected reduction in expression in the relevant contexts measured in units so that $\beta = 1$ is equivalent to the effect of an LoF (note that this sign convention is the opposite of that in [99] because we define γ in terms of the effect of an LoF). For other types of variants (e.g., splice-altering or coding variants), we can measure their effect in terms of how expression of the gene would need to be changed to have a comparable effect. For example, a coding variant that abolishes the function of a protein but does not affect expression would also have a $\beta = 1$ as it is effectively equivalent to an LoF.

In the main text, we assumed that $\alpha = \beta\gamma$, but we have found that for many genes, α appears to depend non-linearly on β [99, 122]. As such, in this appendix we will instead consider γ to be an arbitrary function of β (called the Gene Dosage Response Curve — GDRC — in [99]), with $\alpha = \gamma(\beta)$.

In this context it becomes less obvious how to define gene importance. For example, small perturbations of a gene's expression may have essentially no effect on the trait (i.e., $\gamma(\beta) \approx 0$ for small β) while larger perturbations may have an extreme effect on the trait. Is such a gene more important than a gene where a perturbation of any size results in a moderate impact on the trait?

Yet, there is a case where it is easy to compare the importance of two genes. Consider two genes with GDRCs $\gamma_1(\cdot)$ and $\gamma_2(\cdot)$. If for all β,

$$\gamma_1(\beta)^2 \geq \gamma_2(\beta)^2$$

then it is clear that gene 1 is at least as important as gene 2: when both genes are perturbed by the same amount, the perturbation of gene 1 has at least as large a phenotypic effect as the perturbation of gene 2, regardless of the size of the perturbation.

In this case, the results presented in the "Approaches for estimating trait importance" section in the main text hold under our approximation that variants either affect the trait enough to contribute to heritability or have a negligible contribution to heritability. In particular, any variant with a β such that $\gamma_2(\beta)^2$ is large enough to contribute to heritability through its action on gene 2, would have $\gamma_1(\beta)^2$ large enough to contribute to heritability through gene 1, and hence the total heritability contributed by variants through gene 1 should be at least as large as that contributed

by variants through gene 2, all else being equal. As such, we see again that total heritability should roughly correlate with this notion of importance for genes.

References

[84] Zeng, T., Spence, J. P., Mostafavi, H., and Pritchard, J. K. Bayesian estimation of gene constraint from an evolutionary model with gene features. *Nature Genetics*, 56(8):1632–1643, 2024.

[85] Karczewski, K. J., Francioli, L. C., Tiao, G., Cummings, B. B., Alföldi, J., Wang, Q., Collins, R. L., Laricchia, K. M., Ganna, A., Birnbaum, D. P., and others,. The mutational constraint spectrum quantified from variation in 141,456 humans. *Nature*, 581(7809):434–443, 2020.

[86] Berisa, T. and Pickrell, J. K. Approximately independent linkage disequilibrium blocks in human populations. *Bioinformatics*, 32(2):283, 2016.

[87] Yang, J., Ferreira, T., Morris, A. P., Medland, S. E., of ANthropometric Traits (GIANT) Consortium, G. I., Replication, D. G., analysis (DIAGRAM) Consortium, M., Madden, P. A., Heath, A. C., Martin, N. G., et al. Conditional and joint multiple-snp analysis of gwas summary statistics identifies additional variants influencing complex traits. *Nature genetics*, 44(4):369–375, 2012.

[88] de Leeuw, C. A., Mooij, J. M., Heskes, T., and Posthuma, D. MAGMA: generalized gene-set analysis of GWAS data. *PLoS computational biology*, 11(4):e1004219, 2015.

[89] Weeks, E. M., Ulirsch, J. C., Cheng, N. Y., Trippe, B. L., Fine, R. S., Miao, J., Patwardhan, T. A., Kanai, M., Nasser, J., Fulco, C. P., and others,. Leveraging polygenic enrichments of gene features to predict genes underlying complex traits and diseases. *Nature Genetics*, 55(8):1267–1276, 2023.

[90] Zhu, X. and Stephens, M. Bayesian large-scale multiple regression with summary statistics from genome-wide association studies. *The annals of applied statistics*, 11(3):1561, 2017.

[91] Simons, Y. B., Bullaughey, K., Hudson, R. R., and Sella, G. A population genetic interpretation of GWAS findings for human quantitative traits. *PLoS biology*, 16(3):e2002985, 2018.

[92] Sella, G. and Barton, N. H. Thinking about the evolution of complex traits in the era of genome-wide association studies. *Annual Review of Genomics and Human Genetics*, 20(Volume 20, 2019):461–493, 2019.

[93] Bulik-Sullivan, B. K., Loh, P.-R., Finucane, H. K., Ripke, S., Yang, J., of the Psychiatric Genomics Consortium, S. W. G., Patterson, N., Daly, M. J., Price, A. L., and Neale, B. M. LD Score regression distinguishes confounding from polygenicity in genome-wide association studies. *Nature genetics*, 47(3):291–295, 2015.

[94] Gamazon, E. R., Wheeler, H. E., Shah, K. P., Mozaffari, S. V., Aquino-Michaels, K., Carroll, R. J., Eyler, A. E., Denny, J. C., Consortium, G., Nicolae, D. L., and others,. A gene-based association method for mapping traits using reference transcriptome data. *Nature genetics*, 47(9):1091–1098, 2015.

[95] Gusev, A., Ko, A., Shi, H., Bhatia, G., Chung, W., Penninx, B. W., Jansen, R., De Geus, E. J., Boomsma, D. I., Wright, F. A., and others,. Integrative approaches for large-scale transcriptome-wide association studies. *Nature genetics*, 48(3):245–252, 2016.

[96] Privé, F., Arbel, J., and Vilhjálmsson, B. J. LDpred2: better, faster, stronger. *Bioinformatics*, 36(22-23):5424–5431, 2020.

[97] Wang, G., Sarkar, A., Carbonetto, P., and Stephens, M. A simple new approach to variable selection in regression, with application to genetic fine mapping. *Journal of the Royal Statistical Society Series B: Statistical Methodology*, 82(5):1273–1300, 2020.

[98] Spence, J. P., Sinnott-Armstrong, N., Assimes, T. L., and Pritchard, J. K. A flexible modeling and inference framework for estimating variant effect sizes from GWAS summary statistics. *BioRxiv*, pages 2022–04, 2022.

[99] Milind, N., Smith, C. J., Zhu, H., Spence, J. P., and Pritchard, J. K. Buffering and non-monotonic behavior of gene dosage response curves for human complex traits. *medRxiv*, pages 2024–11, 2024.

[100] Heinze, G. and Schemper, M. A solution to the problem of separation in logistic regression. *Statistics in medicine*, 21(16):2409–2419, 2002.

[101] Gillespie, J. H. *Population genetics: a concise guide*. JHU press, 2004.

[102] Good, B. H. Linkage disequilibrium between rare mutations. *Genetics*, 220(4):iyac004, 2022.

[103] Dominici, D. E. The inverse of the cumulative standard normal probability function. *Integral Transforms and Special Functions*, 14(4):281–292, 2003.

[104] Boyle, E. A., Li, Y. I., and Pritchard, J. K. An expanded view of complex traits: from polygenic to omnigenic. *Cell*, 169(7):1177–1186, 2017.

[105] Schraiber, J. G., Spence, J. P., and Edge, M. D. Estimation of demography and mutation rates from one million haploid genomes. *The American Journal of Human Genetics*, 112(9):2152–2166, 2025.

[106] Pickrell, J. K., Berisa, T., Liu, J. Z., Ségurel, L., Tung, J. Y., and Hinds, D. A. Detection and interpretation of shared genetic influences on 42 human traits. *Nature genetics*, 48(7):709–717, 2016.

[107] Watanabe, K., Stringer, S., Frei, O., Umićević Mirkov, M., de Leeuw, C., Polderman, T. J., van der Sluis, S., Andreassen, O. A., Neale, B. M., and Posthuma, D. A global overview of pleiotropy and genetic architecture in complex traits. *Nature genetics*, 51(9):1339–1348, 2019.

[108] Qi, G., Chhetri, S. B., Ray, D., Dutta, D., Battle, A., Bhattacharjee, S., and Chatterjee, N. Genome-wide large-scale multi-trait analysis characterizes global patterns of pleiotropy and unique trait-specific variants. *Nature Communications*, 15(1):6985, 2024.

[109] Bulmer, M. G. The genetic variability of polygenic characters under optimizing selection, mutation and drift. *Genetics Research*, 19(1):17–25, 1972.

[110] Keightley, P. D. and Hill, W. G. Quantitative genetic variability maintained by mutation-stabilizing selection balance in finite populations. *Genetics Research*, 52(1):33–43, 1988.

[111] Virtanen, P., Gommers, R., Oliphant, T. E., Haberland, M., Reddy, T., Cournapeau, D., Burovski, E., Peterson, P., Weckesser, W., Bright, J., and others,. Scipy 1.0: fundamental algorithms for scientific computing in python. *Nature methods*, 17(3):261–272, 2020.

[112] Clyde, M. *BAS: Bayesian Variable Selection and Model Averaging using Bayesian Adaptive Sampling*, 2024. R package version 1.7.5.

[113] *NIST Digital Library of Mathematical Functions*. `https://dlmf.nist.gov/`, Release 1.2.2 of 2024-09-15. F. W. J. Olver, A. B. Olde Daalhuis, D. W. Lozier, B. I. Schneider, R. F. Boisvert, C. W. Clark, B. R. Miller, B. V. Saunders, H. S. Cohl, and M. A. McClain, eds.

[114] Song, Y. S. Lecture notes on computational and mathematical population genetics. `https://people.eecs.berkeley.edu/~yss/Pub/CMPG_lecture_notes.pdf`, 2021.

[115] Ponnusamy, S. and Vuorinen, M. Asymptotic expansions and inequalities for hypergeometric function. *Mathematika*, 44(2):278–301, 1997.

[116] Negm, S. and Veller, C. The effect of long-range linkage disequilibrium on allele-frequency dynamics under stabilizing selection. *bioRxiv*, pages 2024–06, 2024.

[117] Bulmer, M. G. The effect of selection on genetic variability. *The American Naturalist*, 105(943):201–211, 1971.

[118] Bulmer, M. G. Linkage disequilibrium and genetic variability. *Genetics Research*, 23(3):281–289, 1974.

[119] Brandes, N., Goldman, G., Wang, C. H., Ye, C. J., and Ntranos, V. Genome-wide prediction of disease variant effects with a deep protein language model. *Nature Genetics*, 55(9):1512–1522, 2023.

[120] Cheng, J., Novati, G., Pan, J., Bycroft, C., Žemgulytė, A., Applebaum, T., Pritzel, A., Wong, L. H., Zielinski, M., Sargeant, T., and others,. Accurate proteome-wide missense variant effect prediction with alphamissense. *Science*, 381(6664):eadg7492, 2023.

[121] Beltran, A., Jiang, X., Shen, Y., and Lehner, B. Site saturation mutagenesis of 500 human protein domains reveals the contribution of protein destabilization to genetic disease. *bioRxiv*, pages 2024–04, 2024.

[122] Naqvi, S., Kim, S., Hoskens, H., Matthews, H. S., Spritz, R. A., Klein, O. D., Hallgrímsson, B., Swigut, T., Claes, P., Pritchard, J. K., and others,. Precise modulation of transcription factor levels identifies features underlying dosage sensitivity. *Nature Genetics*, 55(5):841–851, 2023.
