# Supplementary material for: Specificity, length and luck drive gene rankings in association studies
Source: Nature. 2025 Nov 5;649(8098):918–25. doi: 10.1038/s41586-025-09703-7 (PMC12823407; doi:10.1038/s41586-025-09703-7)
Supplement: Supplementary file 1 [file 41586_2025_9703_MOESM2_ESM.pdf]

nature portfolio

Corresponding author(s): Jeffrey P. Spence, Hakhamanesh Mostafavi, Mineto Ota, Jonathan K. Pritchard

Last updated by author(s): 2025/07/02

Reporting Summary

Nature Portfolio wishes to improve the reproducibility of the work that we publish. This form provides structure for consistency and transparency in reporting. For further information on Nature Portfolio policies, see our Editorial Policies and the Editorial Policy Checklist.

Statistics

For all statistical analyses, confirm that the following items are present in the figure legend, table legend, main text, or Methods section.

n/a	Confirmed	
☐	☒	The exact sample size (*n*) for each experimental group/condition, given as a discrete number and unit of measurement
☐	☒	A statement on whether measurements were taken from distinct samples or whether the same sample was measured repeatedly
☐	☒	The statistical test(s) used AND whether they are one- or two-sided<br>*Only common tests should be described solely by name; describe more complex techniques in the Methods section.*
☐	☒	A description of all covariates tested
☐	☒	A description of any assumptions or corrections, such as tests of normality and adjustment for multiple comparisons
☐	☒	A full description of the statistical parameters including central tendency (e.g. means) or other basic estimates (e.g. regression coefficient) AND variation (e.g. standard deviation) or associated estimates of uncertainty (e.g. confidence intervals)
☐	☒	For null hypothesis testing, the test statistic (e.g. *F*, *t*, *r*) with confidence intervals, effect sizes, degrees of freedom and *P* value noted<br>*Give P values as exact values whenever suitable.*
☒	☐	For Bayesian analysis, information on the choice of priors and Markov chain Monte Carlo settings
☒	☐	For hierarchical and complex designs, identification of the appropriate level for tests and full reporting of outcomes
☐	☒	Estimates of effect sizes (e.g. Cohen's *d*, Pearson's *r*), indicating how they were calculated

Our web collection on statistics for biologists contains articles on many of the points above.

Software and code

Policy information about availability of computer code

Data collection	No software was used for data collection
Data analysis	Scripts for analyzing and plotting the data are provided on GitHub (https://github.com/jeffspence/specificity_length_luck). All statistical analyses were performed using R v. 4.4.0 with packages data.table v1.15.4, ggplot2 v3.5.2, tidyverse v2.0.0, dplyr v1.1.4, cowplot v1.1.3, scales v1.3.0, viridis v0.6.5, gridExtra v2.3, patchwork v1.2.0, RColorBrewer v1.1-3, ggpmisc v0.6.0, ggrastr v1.0.2, BAS v1.7.1, and R.utils v2.12.3; and python v. 3.9.6 with packages fastDTWF v0.0.6, numpy v1.22.4, scipy v1.12.0, statsmodels v0.14.0, and pandas v2.2.2. We performed additional analyses using S-LDSC v. 1.0.1, AMM (commit 524c620), MAMGA (v1.10), and PoPS (v0.2).

For manuscripts utilizing custom algorithms or software that are central to the research but not yet described in published literature, software must be made available to editors and reviewers. We strongly encourage code deposition in a community repository (e.g. GitHub). See the Nature Portfolio guidelines for submitting code & software for further information.

Data

Policy information about availability of data

All manuscripts must include a data availability statement. This statement should provide the following information, where applicable:

- Accession codes, unique identifiers, or web links for publicly available datasets
- A description of any restrictions on data availability
- For clinical datasets or third party data, please ensure that the statement adheres to our policy

All data and scripts for reproducing the figures are provided at https://github.com/jeffspence/specificity_length_luck

Research involving human participants, their data, or biological material

Policy information about studies with human participants or human data. See also policy information about sex, gender (identity/presentation), and sexual orientation and race, ethnicity and racism.

Reporting on sex and gender	N/A
Reporting on race, ethnicity, or other socially relevant groupings	N/A
Population characteristics	N/A
Recruitment	N/A
Ethics oversight	N/A

Note that full information on the approval of the study protocol must also be provided in the manuscript.

Field-specific reporting

Please select the one below that is the best fit for your research. If you are not sure, read the appropriate sections before making your selection.

☒ Life sciences ☐ Behavioural & social sciences ☐ Ecological, evolutionary & environmental sciences

For a reference copy of the document with all sections, see nature.com/documents/nr-reporting-summary-flat.pdf

Life sciences study design

All studies must disclose on these points even when the disclosure is negative.

Sample size	All analyses were performed on previously collected data from the UK Biobank. In all analyses we used all data that passed filtering criteria as described in the methods.
Data exclusions	Some analyses used a subset of genetically uncorrelated traits to ensure that the traits were relatively non-redundant. For some analyses we used LD-pruned variants to ensure relatively independent signals for ease of statistical analysis. We also performed some analyses on a subset of traits that we could confidently match to a single tissue, as described in the methods. The rationale for these exclusions and the criteria used are described in the methods.
Replication	Most of our results are supported by two or more complementary analyses to ensure that they are not artifacts of specific analyses.
Randomization	We used publicly available data and were not involved in data collection. As result, randomization is not relevant for our study.
Blinding	We used publicly available data and were not involved in data collection. As result, blinding is not relevant for our study.

Reporting for specific materials, systems and methods

We require information from authors about some types of materials, experimental systems and methods used in many studies. Here, indicate whether each material, system or method listed is relevant to your study. If you are not sure if a list item applies to your research, read the appropriate section before selecting a response.

Materials & experimental systems

n/a	Involved in the study
☒	☐ Antibodies
☒	☐ Eukaryotic cell lines
☒	☐ Palaeontology and archaeology
☒	☐ Animals and other organisms
☒	☐ Clinical data
☒	☐ Dual use research of concern
☒	☐ Plants

Methods

n/a	Involved in the study
☒	☐ ChIP-seq
☒	☐ Flow cytometry
☒	☐ MRI-based neuroimaging

Plants

Seed stocks

Report on the source of all seed stocks or other plant material used. If applicable, state the seed stock centre and catalogue number. If plant specimens were collected from the field, describe the collection location, date and sampling procedures.

Novel plant genotypes

Describe the methods by which all novel plant genotypes were produced. This includes those generated by transgenic approaches, gene editing, chemical/radiation-based mutagenesis and hybridization. For transgenic lines, describe the transformation method, the number of independent lines analyzed and the generation upon which experiments were performed. For gene-edited lines, describe the editor used, the endogenous sequence targeted for editing, the targeting guide RNA sequence (if applicable) and how the editor was applied.

Authentication

Describe any authentication procedures for each seed stock used or novel genotype generated. Describe any experiments used to assess the effect of a mutation and, where applicable, how potential secondary effects (e.g. second site T-DNA insertions, mosiacism, off-target gene editing) were examined.
